# Supplementary material for: Association between vitamin D deficiency and benign paroxysmal positional vertigo (BPPV) incidence and recurrence: a systematic review and meta-analysis
Source: BMJ Open. 2024 Apr 22;14(4):e077986. doi: 10.1136/bmjopen-2023-077986 (PMC11043747; doi:10.1136/bmjopen-2023-077986)
Supplement: Supplementary data [file bmjopen-2023-077986supp001.pdf]

NIHR | National Institute for Health Research

PROSPERO
International prospective register of systematic reviews

Association between vitamin D deficiency and benign paroxysmal positional vertigo: a systematic review and meta-analysis

Citation

Heather Wood, Ghada BinKhamis, Karolina Kluk De-Kort. Association between vitamin D deficiency and benign paroxysmal positional vertigo: a systematic review and meta-analysis. PROSPERO 2021 CRD42021271840 Available from: https://www.crd.york.ac.uk/prospero/display_record.php?ID=CRD42021271840

Review question

How do serum vitamin D levels affect the incidence and recurrence rates of benign paroxysmal positional vertigo (BPPV) in adult patients in the Northern Hemisphere?

Searches

We will search the following electronic bibliographic databases: PubMed, NICE, Scopus, Web of Science using the following search terms: "Vitamin D* OR 25-hydroxyvitamin D OR ergocalciferol OR cholecalciferol AND benign paroxysmal positional vertigo", "Vitamin D* OR 25-hydroxyvitamin D OR ergocalciferol OR cholecalciferol AND BPPV" "Vitamin D* OR 25-hydroxyvitamin D OR ergocalciferol OR cholecalciferol AND vertigo"

Research papers published between 2000 and 2021 will be reviewed and language is restricted with English.

- Searches will be re-run prior to the final analysis to identify any further studies.

- Unpublished studies will not be sought

Types of study to be included

Randomised controlled trial, cohort study or case-controlled study

Condition or domain being studied

Benign Paroxysmal positional vertigo (BPPV) and serum vitamin D levels

Participants/population [2 changes]

Inclusion criteria:

- Participants over the age of 18 with at least one episode of diagnosed benign paroxysmal positional vertigo (BPPV).

- Serum 25-hydroxyvitamin D levels measured and reported.

- Geographical location of study participants in Northern hemisphere.

- Participants not taking vitamin D supplements,

- No other comorbid diagnoses.

Exclusion criteria:

- Participants below the age of 18 with at least one episode of BPPV.

- Serum 25-hydroxyvitamin D levels not measured or reported.

- Geographical location of study participants not in Northern hemisphere.

- Participants taking vitamin D supplements,

- Participants with other comorbid diagnoses.

Intervention(s), exposure(s)

Serum 25-hydroxyvitamin D levels

Comparator(s)/control

Normal controls over the age of 18 with no past medical history of benign paroxysmal positional vertigo

Context

Studies in hospital ENT outpatient departments. Research including participants in southern hemisphere countries will be excluded

Main outcome(s)

Incidence of benign paroxysmal positional vertigo. Recurrence of benign paroxysmal positional vertigo

Measures of effect

Difference in average serum vitamin D levels between patients with new or recurrent benign paroxysmal positional vertigo compared to controls.

Additional outcome(s)

Not applicable

Data extraction (selection and coding)

Study selection: one author will select and this selection will be reviewed by two authors.

The final decision will be recorded in an excel spreadsheet.

Any disagreements will be resolved by discussion between the authors.

Extracted data will include:

Study design; country; author and published year; mean age of participants; range of participant age; sample size; disease diagnosis; percentage male participants and serum vitamin D level for cases and controls.

Risk of bias (quality) assessment

Any randomised controlled trials will be assessed using the Cochrane Collaboration's tool for assessing risk of bias in randomised trials.

Case-control studies will be assessed using the Risk of Bias Assessment Tool for Nonrandomized Studies (RoBANS) tool.

Cohort studies will be assessed according to the Newcastle Ottawa Scale (NOS).

Records of risk of bias will be recorded in Microsoft Word and Microsoft Excel files.

Strategy for data synthesis

Standardised mean difference (SMD) with a 95% confidence interval will be used to calculate the level of vitamin D in case and control groups. Plasma vitamin D levels of greater than 20 ng/ml will be considered to be sufficient. The Q statistic will be used to examine heterogeneity between studies, with P<0.10 regarded as significant. The random-effects model will be employed for the meta-analyses if P<0.10, otherwise the fixed-effect model will be used instead.

Statistical analysis will be completed using RevMan software version 5.4.1

Analysis of subgroups or subsets

Vitamin D status will be categorised into "sufficient" if serum levels are >20ng/l; or "insufficient" if serum levels <20ng/L

There may be differences between patients experiencing recurrent BPPV and those experiencing single episodes. Recurrent BPPV will be classified as symptom recurrence within 3 years of initial diagnosis.

Contact details for further information

Heather Wood

hmw998@student.bham.ac.uk

Organisational affiliation of the review

University of Birmingham

Review team members and their organisational affiliations [1 change]

Ms Heather Wood. University of Birmingham, Birmingham, UK

Dr Ghada BinKhamis. University of Manchester, Manchester, UK. King Fahad Medical City, Riyadh, Saudi Arabia

Dr Karolina Kluk De-Kort. University of Manchester, Manchester, UK

Type and method of review

Meta-analysis, Systematic review

Anticipated or actual start date

09 August 2021

Anticipated completion date [1 change]

01 September 2023

Funding sources/sponsors

None

Conflicts of interest

Language

English

Country

England

Stage of review

Review Ongoing

Subject index terms status

Subject indexing assigned by CRD

Subject index terms

Benign Paroxysmal Positional Vertigo; Humans; Vitamin D Deficiency

Date of registration in PROSPERO

06 August 2021

Date of first submission

04 August 2021

Stage of review at time of this submission [3 changes]

Stage	Started	Completed
Preliminary searches	Yes	Yes
Piloting of the study selection process	Yes	Yes
Formal screening of search results against eligibility criteria	Yes	Yes
Data extraction	Yes	No
Risk of bias (quality) assessment	Yes	Yes
Data analysis	Yes	No

Revision note

Updating progress to date - checked boxes to indicate that screening and assessment of bias are complete and that data extraction and analysis have been started.

The record owner confirms that the information they have supplied for this submission is accurate and complete and they understand that deliberate provision of inaccurate information or omission of data may be construed as scientific misconduct.

The record owner confirms that they will update the status of the review when it is completed and will add publication details in due course.

Versions

06 August 2021
09 August 2021
14 September 2021
27 October 2021
21 February 2023
01 April 2023

Wood H, *et al. BMJ Open* 2024; 14:e077986. doi: 10.1136/bmjopen-2023-077986
