# Supplementary material for: Association between vitamin D deficiency and benign paroxysmal positional vertigo (BPPV) incidence and recurrence: a systematic review and meta-analysis
Source: BMJ Open. 2024 Apr 22;14(4):e077986. doi: 10.1136/bmjopen-2023-077986 (PMC11043747; doi:10.1136/bmjopen-2023-077986)
Supplement: Supplementary data [file bmjopen-2023-077986supp002.pdf]

Supplementary 2

Supplementary 2, Table 1. Systematic review searches performed and results.

Search	*Search terms*	*Database*	*Results*	*Filters*
**1**	(Vitamin D OR 25-hydroxyvitamin D OR ergocalciferol OR cholecalciferol) AND BPPV	PUBMED	86	Years 2000 - 2023, English Language
**2**	(Vitamin D OR 25-hydroxyvitamin D OR ergocalciferol OR cholecalciferol) AND "Benign paroxysmal positional vertigo"	PUBMED	86	Years 2000 - 2023, English Language
**3**	(Vitamin D OR 25-hydroxyvitamin D OR ergocalciferol OR cholecalciferol) AND vertigo	PUBMED	102	Years 2000 - 2023, English Language
**4**	(Vitamin D* OR 25-hydroxyvitamin D OR ergocalciferol OR cholecalciferol) AND BPPV	SCOPUS	85	Year >1999, English Language
**5**	(Vitamin D* OR 25-hydroxyvitamin D OR ergocalciferol OR cholecalciferol) AND "Benign paroxysmal positional vertigo"	SCOPUS	104	Year >1999, English Language
**6**	(Vitamin D* OR 25-hydroxyvitamin D OR ergocalciferol OR cholecalciferol) AND "vertigo"	SCOPUS	300	Year >1999, English Language
**7**	(ALL=(Vitamin D OR 25-hydroxyvitamin D OR ergocalciferol OR cholecalciferol)) AND ALL=(BPPV)	Web of Science	101	Timespan: 2000-01-01 to2023-02-18, English Language
**8**	(ALL=(Vitamin D OR 25-hydroxyvitamin D OR ergocalciferol OR cholecalciferol)) AND ALL=(Benign Paroxysmal Positional Vertigo)	Web of Science	108	Timespan: 2000-01-01 to 2023-02-18, English Language
**9**	(ALL=(Vitamin D OR 25-hydroxyvitamin D OR ergocalciferol OR cholecalciferol)) AND ALL=(Vertigo)	Web of Science	141	Timespan: 2000-01-01 to2023-02-18, English Language

Supplementary 2, Table 2. Data extracted from included studies for the Meta-analysis exploring the relationship between Vitamin D deficiency and BPPV occurrence.

	Participants with BPPV			Control participants (without BPPV)		
Study	Number of Participants	Vitamin D (Mean: ng/mL)	Vitamin D (SD: ng/mL)	Number of Participants	Vitamin D (Mean: ng/mL)	Vitamin D (SD: ng/mL)
Bi et al. 2021	27	14.64	6.94	25	19.56	6.55
Califano et al. 2019	127	21.08	11	100	25.6	10.5
Çelik et al. 2021	190	15.64	8.4	149	16.29	6.7
Ceylan and Kanmaz 2020	97	15.87	8.61	100	15.82	10.23
Cheng et al. 2021	320	23.2	4.09	320	25.8	3.43
Çıkrıkçı Işık et al. 2017	64	9.51	5.49	63	11.02	9.62
Goldschagg et al. 2021	158	23.4	9.4	301	21.5	10.6
Han et al. 2018	85	19.1	5.2	80	22.5	5.8
Inan et al. 2021	52	15.3	9.8	52	20.2	14.3
Karataş et al. 2017	78	23	14.4	78	17	12.3
Sarsitthithim et al. 2021	69	21.5	5.3	68	26.3	6.8
Sen, Padiyar and Arora 2018	100	20.3	12.2	100	18	10.1
Song et al. 2020	380	14.24	6.6	3125	15.45	7.7
Talaat et al. 2015	80	14.19	9.3	100	19.53	8.45
Thomas et al. 2021	49	21.3	9.567	49	17.6	8.061
Wang et al. 2020	103	17.15	2.03	80	23.85	3.13
Wu et al. 2018	60	20.99	6.76	92	23.17	6.49
Yang et al. 2017	130	18.21	10.3	130	20	8.1
Zhang et al. 2022	156	18.8	2.5	50	24	2.2

Supplementary 2, Table 3. Data extracted from included studies for the Meta-analysis exploring the relationship between Vitamin D deficiency and BPPV recurrence.

	Participants with BPPV Recurrence			Participants without BPPV Recurrence		
**Study**	**Number of Participants**	**Vitamin D (Mean: ng/mL)**	**Vitamin D (SD: ng/mL)**	**Number of Participants**	**Vitamin D (Mean: ng/mL)**	**Vitamin D (SD: ng/mL)**
Çıkrıkçı Işık et al. 2017	23	8.81	4.9	41	9.91	5.81
Melis et al. 2020	43	19.53	15.33	30	25.85	14.1
Sarsitthithim et al. 2021	41	21.9	4.9	28	21	5.9
Shin *et al.* 2023	19	12.9	8	31	19.2	8.2
Talaat et al. 2015	36	11.93	7.57	44	16.04	10.26
Yang et al. 2017	63	19.3	11.1	67	17.2	9.4
Zhang *et al.* 2022	30	16.7	2.1	126	19.3	2.3

	Selection of Participants	Confounding Variables	Intervention measurement	Blinding of outcome assessment	Incomplete outcome data	Selective outcome reporting
**Bi *et al.* 2021**	-	?	-	+	-	-
**Califano *et al.* 2019**	?	+	-	+	-	-
**Carneiro de Sousa *et al.* 2019**	-	+	-	+	-	-
**Çelik *et al*. 2021**	-	-	-	+	-	-
**Ceylan 2020**	-	+	-	+	-	-
**Cheng *et al*. 2021**	-	?	-	+	-	-
**Çıkrıkçı Işık *et al.* 2017**	-	-	-	+	-	-
**Ding *et al.* 2019**	-	-	-	+	-	-
**Elmoursey and Abbas 2021**	-	+	-	+	-	-
**Goldschlagg *et al.* 2021**	-	?	-	+	-	-
**Gu, Dong and Gu 2018**	?	-	-	-	-	-
**Han *et al.* 2018**	-	?	-	+	-	-
**Inan *et al.* 2021**	?	-	-	+	-	-
**Karatas *et al.* 2016**	?	-	-	+	-	-
**Parham *et al.* 2013**	?	-	-	-	-	-
**Pecci *et al.* 2022**	+	-	-	+	-	-
**Sarsitthithim *et al.* 2021**	?	?	-	+	-	-
**Sen, Padiyar and Arora 2018**	?	?	-	+	-	-
**Song *et al*. 2020**	-	-	-	+	-	-
**Talaat *et al*. 2015**	-	-	-	+	-	-
**Thomas *et al*. 2021**	-	?	-	+	-	-
**Wang *et al*. 2020**	?	-	-	+	-	-
**Wu *et al*. 2018**	-	-	-	+	-	-
**Wu *et al*. 2022**	-	-	-	+	-	-
**Yang *et al*. 2017**	?	-	-	+	-	-
**Zhang *et al*. 2022**	-	-	-	+	-	-

Supplementary 2, Figure 1. Assessment of bias for case-control studies according to RoBANs.
Green '-' symbols= low risk of bias, red '+' symbols= high risk of bias and purple '?' symbols = unclear risk of bias.

	Representativeness of exposed cohort	Selection of non-exposed cohort	Ascertainment of exposure	Outcome of interest not present at start	Comparability of cohorts/confounders	Assessment of outcome	Follow-up long enough?	Adequacy of follow-up of cohorts	Study Quality
Han *et al.* 2020	*	*	*	-	**	*	*	n/a	Good
Han *et al*. 2021	-	*	*	-	**	*	*	n/a	Fair
Kahraman *et al.* 2016	*	*	*	-	*	*	*	*	Good
Lee *et al.* 2017	-	*	*	-	**	*	*	n/a	Fair
Maslovara *et al.* 2018	*	*	*	*	**	*	*	*	Good
Melis *et al.* 2020	*	*	*	*	**	*	-	*	Good
Nakada *et al*. 2019	*	*	*	-	-	*	*	n/a	Poor
Shin *et al*. 2023	*	*	*	-	*	*	*	*	Good
Shu *et al*. 2019	*	*	*	-	**	*	*	n/a	Good

Supplementary 2, Figure 2. Assessment of bias of cohort studies using the Newcastle-Ottawa Quality Assessment form for cohort studies.
'*' symbols indicate low risk of bias for a given criteria, '-' symbols indicate the study does not meet criteria for low risk of bias and 'n/a' indicates that the criterion does not apply to a given study (for example retrospective studies do not have a follow-up period by design).

Supplementary 2, Table 4. PRISMA Checklist

Section and Topic	Item #	Checklist item	Location where item is reported
**TITLE**			
Title	1	Identify the report as a systematic review.	Title page (p1)
**ABSTRACT**			
Abstract	2	See the PRISMA 2020 for Abstracts checklist.	Abstract (p2)
**INTRODUCTION**			
Rationale	3	Describe the rationale for the review in the context of existing knowledge.	Introduction (p4)
Objectives	4	Provide an explicit statement of the objective(s) or question(s) the review addresses.	Introduction (p4)
**METHODS**			
Eligibility criteria	5	Specify the inclusion and exclusion criteria for the review and how studies were grouped for the syntheses.	Methods: Inclusion and Exclusion criteria (p5)
Information sources	6	Specify all databases, registers, websites, organisations, reference lists and other sources searched or consulted to identify studies. Specify the date when each source was last searched or consulted.	Methods: Data sources and search strategy (p5)
Search strategy	7	Present the full search strategies for all databases, registers and websites, including any filters and limits used.	Methods: Data sources and search strategy (p5)
Selection process	8	Specify the methods used to decide whether a study met the inclusion criteria of the review, including how many reviewers screened each record and each report retrieved, whether they worked independently, and if applicable, details of automation tools used in the process.	Methods: Inclusion and Exculsion criteria (p5)
Data collection process	9	Specify the methods used to collect data from reports, including how many reviewers collected data from each report, whether they worked independently, any processes for obtaining or confirming data from study investigators, and if applicable, details of automation tools used in the process.	Methods: Data Extraction (p6)
Data items	10a	List and define all outcomes for which data were sought. Specify whether all results that were compatible with each outcome domain in each study were sought (e.g. for all measures, time points, analyses), and if not, the methods used to decide which results to collect.	Methods: Data Extraction (p6)
	10b	List and define all other variables for which data were sought (e.g. participant and intervention characteristics, funding sources). Describe any assumptions made about any missing or unclear information.	Methods: Data Extraction (p6)
Study risk of bias assessment	11	Specify the methods used to assess risk of bias in the included studies, including details of the tool(s) used, how many reviewers assessed each study and whether they worked independently, and if applicable, details of automation tools used in the process.	Methods: Assessment of bias (p6)
Effect measures	12	Specify for each outcome the effect measure(s) (e.g. risk ratio, mean difference) used in the synthesis or presentation of results.	Methods: Synthesis methods (p6)
Synthesis methods	13a	Describe the processes used to decide which studies were eligible for each synthesis (e.g. tabulating the study intervention characteristics and comparing against the planned groups for each synthesis (item #5)).	Methods: Synthesis methods (p6)
	13b	Describe any methods required to prepare the data for presentation or synthesis, such as handling of missing summary statistics, or data conversions.	Methods: Synthesis methods (p6)

Wood H, *et al. BMJ Open* 2024; 14:e077986. doi: 10.1136/bmjopen-2023-077986

Section and Topic	Item #	Checklist item	Location where item is reported
	13c	Describe any methods used to tabulate or visually display results of individual studies and syntheses.	Methods: Synthesis methods (p6)
	13d	Describe any methods used to synthesize results and provide a rationale for the choice(s). If meta-analysis was performed, describe the model(s), method(s) to identify the presence and extent of statistical heterogeneity, and software package(s) used.	Methods: Synthesis methods (p6)
	13e	Describe any methods used to explore possible causes of heterogeneity among study results (e.g. subgroup analysis, meta-regression).	Not applicable
	13f	Describe any sensitivity analyses conducted to assess robustness of the synthesized results.	Not applicable
Reporting bias assessment	14	Describe any methods used to assess risk of bias due to missing results in a synthesis (arising from reporting biases).	Not applicable
Certainty assessment	15	Describe any methods used to assess certainty (or confidence) in the body of evidence for an outcome.	Not applicable
**RESULTS**			
Study selection	16a	Describe the results of the search and selection process, from the number of records identified in the search to the number of studies included in the review, ideally using a flow diagram.	Results: Study selection (p6-7); Figure 1. Flow diagram detailing results of searches and study selection process; Supplementary 2. Systematic review searches performed and results
	16b	Cite studies that might appear to meet the inclusion criteria, but which were excluded, and explain why they were excluded.	Results: Study selection (p6-7); Figure 1. Flow diagram detailing results of searches and study selection process; Supplementary 2. Systematic review searches performed and results
Study characteristics	17	Cite each included study and present its characteristics.	Results: Description of included studies (p7-8)
Risk of bias in studies	18	Present assessments of risk of bias for each included study.	Results: Quality assessment (p7); Supplementary 2 Assessment of bias for case-control studies according to RoBANs; Supplementary 2 Assessment of bias of cohort studies using the Newcastle-Ottawa Quality Assessment form for cohort studies

Section and Topic	Item #	Checklist item	Location where item is reported
Results of individual studies	19	For all outcomes, present, for each study: (a) summary statistics for each group (where appropriate) and (b) an effect estimate and its precision (e.g. confidence/credible interval), ideally using structured tables or plots.	Table 1. Characteristics of included studies
Results of syntheses	20a	For each synthesis, briefly summarise the characteristics and risk of bias among contributing studies.	Table 1. Characteristics of included studies<br>Figure 3. Relationship between serum 25-hydroxyvitamin D and Benign Paroxysmal Positional Vertigo recurrence.
	20b	Present results of all statistical syntheses conducted. If meta-analysis was done, present for each the summary estimate and its precision (e.g. confidence/credible interval) and measures of statistical heterogeneity. If comparing groups, describe the direction of the effect.	Results: Meta analysis (p8-9)<br>Figure 3. Relationship between serum 25-hydroxyvitamin D and Benign Paroxysmal Positional Vertigo recurrence.
	20c	Present results of all investigations of possible causes of heterogeneity among study results.	Not applicable
	20d	Present results of all sensitivity analyses conducted to assess the robustness of the synthesized results.	Not applicable
Reporting biases	21	Present assessments of risk of bias due to missing results (arising from reporting biases) for each synthesis assessed.	Not applicable
Certainty of evidence	22	Present assessments of certainty (or confidence) in the body of evidence for each outcome assessed.	Not applicable
**DISCUSSION**			
Discussion	23a	Provide a general interpretation of the results in the context of other evidence.	Discussion (p11-12)
	23b	Discuss any limitations of the evidence included in the review.	Strengths and limitations of the study (p3); Discussion (p11-12)
	23c	Discuss any limitations of the review processes used.	Strengths and limitations (p3)
	23d	Discuss implications of the results for practice, policy, and future research.	Discussion (p11-12)
**OTHER INFORMATION**			Prospero registration number (p.2)
Registration and protocol	24a	Provide registration information for the review, including register name and registration number, or state that the review was not registered.	Abstract: Trial registration (p2)
	24b	Indicate where the review protocol can be accessed, or state that a protocol was not prepared.	Prospero registration number (p.2)
	24c	Describe and explain any amendments to information provided at registration or in the protocol.	Prospero registration number (p.2)
Support	25	Describe sources of financial or non-financial support for the review, and the role of the funders or sponsors in the review.	Funding sources (p14)

Section and Topic	Item #	Checklist item	Location where item is reported
Competing interests	26	Declare any competing interests of review authors.	Competing interests statement (p14)
Availability of data, code and other materials	27	Report which of the following are publicly available and where they can be found: template data collection forms; data extracted from included studies; data used for all analyses; analytic code; any other materials used in the review.	Methods: Synthesis methods (p6) and Supplementary 2 tables 2 and 3

From: Page MJ, McKenzie JE, Bossuyt PM, Boutron I, Hoffmann TC, Mulrow CD, et al. The PRISMA 2020 statement: an updated guideline for reporting systematic reviews. BMJ 2021;372:n71. doi: 10.1136/bmj.n71

For more information, visit: http://www.prisma-statement.org/

Supplementary 2, Table 5. PRISMA Abstract Checklist

Section and Topic	Item #	Checklist item	Reported (Yes/No)
**TITLE**			
Title	1	Identify the report as a systematic review.	YES
**BACKGROUND**			
Objectives	2	Provide an explicit statement of the main objective(s) or question(s) the review addresses.	YES
**METHODS**			
Eligibility criteria	3	Specify the inclusion and exclusion criteria for the review.	YES
Information sources	4	Specify the information sources (e.g. databases, registers) used to identify studies and the date when each was last searched.	YES
Risk of bias	5	Specify the methods used to assess risk of bias in the included studies.	YES
Synthesis of results	6	Specify the methods used to present and synthesise results.	YES
**RESULTS**			
Included studies	7	Give the total number of included studies and participants and summarise relevant characteristics of studies.	YES
Synthesis of results	8	Present results for main outcomes, preferably indicating the number of included studies and participants for each. If meta-analysis was done, report the summary estimate and confidence/credible interval. If comparing groups, indicate the direction of the effect (i.e. which group is favoured).	YES
**DISCUSSION**			
Limitations of evidence	9	Provide a brief summary of the limitations of the evidence included in the review (e.g. study risk of bias, inconsistency and imprecision).	YES
Interpretation	10	Provide a general interpretation of the results and important implications.	YES
**OTHER**			
Funding	11	Specify the primary source of funding for the review.	YES
Registration	12	Provide the register name and registration number.	YES

From: Page MJ, McKenzie JE, Bossuyt PM, Boutron I, Hoffmann TC, Mulrow CD, et al. The PRISMA 2020 statement: an updated guideline for reporting systematic reviews. BMJ 2021;372:n71. doi: 10.1136/bmj.n71

For more information, visit: http://www.prisma-statement.org/
