# Supplementary material for: Association between vitamin D deficiency and benign paroxysmal positional vertigo (BPPV) incidence and recurrence: a systematic review and meta-analysis
Source: BMJ Open. 2024 Apr 22;14(4):e077986. doi: 10.1136/bmjopen-2023-077986 (PMC11043747; doi:10.1136/bmjopen-2023-077986)
Supplement: Author's manuscript [file bmjopen-2023-077986.draft_revisions.pdf]

BMJ Open

BMJ Open is committed to open peer review. As part of this commitment we make the peer review history of every article we publish publicly available.

When an article is published we post the peer reviewers' comments and the authors' responses online. We also post the versions of the paper that were used during peer review. These are the versions that the peer review comments apply to.

The versions of the paper that follow are the versions that were submitted during the peer review process. They are not the versions of record or the final published versions. They should not be cited or distributed as the published version of this manuscript.

BMJ Open is an open access journal and the full, final, typeset and author-corrected version of record of the manuscript is available on our site with no access controls, subscription charges or pay-per-view fees (http://bmjopen.bmj.com).

If you have any questions on BMJ Open's open peer review process please email info.bmjopen@bmj.com

BMJ Open

Association between vitamin D deficiency and Benign Paroxysmal Positional Vertigo (BPPV) incidence and recurrence: A systematic review and meta-analysis

Journal:	*BMJ Open*
Manuscript ID	bmjopen-2023-077986
Article Type:	Original research
Date Submitted by the Author:	21-Jul-2023
Complete List of Authors:	Wood, Heather; University of Birmingham, Institute of Inflammation and Ageing<br>Kluk, Karolina; The University of Manchester, Manchester Centre for Audiology and Deafness<br>Binkhamis, Ghada; The University of Manchester, Manchester Centre for Audiology and Deafness (ManCAD) ; King Fahad Medical City, Communication and Swallowing Disorders
Keywords:	Audiology < OTOLARYNGOLOGY, Adult otolaryngology < OTOLARYNGOLOGY, Systematic Review

SCHOLARONE™
Manuscripts

BMJ

I, the Submitting Author has the right to grant and does grant on behalf of all authors of the Work (as defined in the below author licence), an exclusive licence and/or a non-exclusive licence for contributions from authors who are: i) UK Crown employees; ii) where BMJ has agreed a CC-BY licence shall apply, and/or iii) in accordance with the terms applicable for US Federal Government officers or employees acting as part of their official duties; on a worldwide, perpetual, irrevocable, royalty-free basis to BMJ Publishing Group Ltd ("BMJ") its licensees and where the relevant Journal is co-owned by BMJ to the co-owners of the Journal, to publish the Work in this journal and any other BMJ products and to exploit all rights, as set out in our licence.

The Submitting Author accepts and understands that any supply made under these terms is made by BMJ to the Submitting Author unless you are acting as an employee on behalf of your employer or a postgraduate student of an affiliated institution which is paying any applicable article publishing charge ("APC") for Open Access articles. Where the Submitting Author wishes to make the Work available on an Open Access basis (and intends to pay the relevant APC), the terms of reuse of such Open Access shall be governed by a Creative Commons licence – details of these licences and which Creative Commons licence will apply to this Work are set out in our licence referred to above.

Other than as permitted in any relevant BMJ Author's Self Archiving Policies, I confirm this Work has not been accepted for publication elsewhere, is not being considered for publication elsewhere and does not duplicate material already published. I confirm all authors consent to publication of this Work and authorise the granting of this licence.

For peer review only

Association between vitamin D deficiency and Benign Paroxysmal Positional Vertigo (BPPV) incidence and recurrence: A systematic review and meta-analysis

Heather Wood[1], Karolina Kluk[2], Ghada BinKhamis[2,3]

[1]*Institute of Inflammation and Ageing, University of Birmingham, United Kingdom;* [2]*Manchester Centre for Audiology and Deafness, Manchester Academic Health Science Centre, University of Manchester, Manchester, UK;* [3]*Department of Communication and Swallowing Disorders, King Fahad Medical City, Riyadh, Saudi Arabia*

Address for correspondence:

Ghada BinKhamis: ghada.binkhamis@manchester.ac.uk / gbinkhamis@gmail.com

Karolina Kluk: Karolina.Kluk@manchester.ac.uk

Heather Wood: hmw998@student.bham.ac.uk

Manchester Centre for Audiology and Deafness (ManCAD), School of Health Sciences, Faculty of Biology Medicine and Health, Room B2.15, Ellen Wilkinson Building, University of Manchester, Oxford Road, Manchester, M13 9PL, United Kingdom.

ORCiD:

- Ghada BinKhamis: 0000-0002-7696-1073
- Karolina Kluk: 0000-0003-3638-2787

Word Count: 3753

For peer review only

Abstract

INTRODUCTION: A systematic review was performed to determine the relationship between serum vitamin D level and the risk of developing benign paroxysmal positional vertigo (BPPV) incidence and recurrence in countries in the Northern hemisphere.

METHODS: PUBMED, SCOPUS and Web of Science databases were searched for studies evaluating the association between serum vitamin D level and BPPV published between January 2000 and February 2023. Inclusion criteria: participants located in the Northern hemisphere aged 18 or over with at least one episode of BPPV, serum 25-hydroxyvitamin D levels measured and reported, no comorbidities or history of vitamin D supplementation. Risk of bias was assessed using the Newcastle Ottawa Tool for Cohort studies and RoBANS checklist for case-control studies. Standard mean difference with a 95% confidence interval was used to measure the relationship between vitamin D level and the risk of BPPV.

RESULTS: The 35 articles identified by the literature search reported data of 9843 individuals. 19 studies describing 7387 individuals were included in the meta-analysis of BPPV incidence whilst seven studies describing 615 individuals were included in the meta-analysis of BPPV recurrence. Lower levels of serum vitamin D were not associated with BPPV recurrence or incidence.

DISCUSSION: The results of this systematic review and meta-analysis did not demonstrate any relationship between BPPV and serum vitamin D. Risk of bias analysis revealed evidence was of variable quality. There was insufficient data available to evaluate seasonal relationships between serum vitamin D and BPPV. Given the potential for this as a confounding factor, future research should aim to investigate this further.

PROSPERO REGISTRATION: CRD42021271840

Strengths and limitations of the study

- Strengths:
  - This is a comprehensive systematic review of vitamin D and Benign Paroxysmal Positional Vertigo (BPPV) covering literature published between January 2000 and February 2023
  - The meta-analysis includes a large number of individuals (7387)
- Limitations:
  - It was not possible to account for the impact of seasonality on both serum vitamin D levels and incidence and recurrence rates of BPPV as the majority of studies did not report the dates or months when patients were seen.

Keywords:

BPPV, Benign Paroxysmal Positional Vertigo, Vitamin D, Vitamin D Deficiency

For peer review only - http://bmjopen.bmj.com/site/about/guidelines.xhtml

Introduction

The impact of vitamin D on audiovestibular health has been a research area of interest in recent years, with low serum levels of vitamin D associated with otological pathologies including benign paroxysmal positional vertigo (BPPV).[1] Due to vitamin D's role in skeletal mineral homeostasis, it has been suggested that audiovestibular pathology may be related to high bone turnover rates in the temporal bone and/or at a systemic level.[2] Another hypothesis suggests that inner ear pathology including BPPV may be secondary to an inflammatory or autoimmune response following viral infection, and that the immunomodulatory functions of vitamin D may be protective against this secondary response.[3]

There are two major bioavailable forms of vitamin D; vitamin D2 (also known as ergocalciferol) and vitamin D3 (cholecalciferol). Ergocalciferol can be ingested via diet or supplementation, whilst vitamin D3 is largely synthesised in human skin following exposure to UVB light (and may also be ingested via dietary supplements or animal-based foods).[4] The bioactive form of vitamin D3 is known to play an important role in calcium homeostasis and bone health; increasing the absorption of dietary calcium and phosphorus which in turn affects the regulation of bone mineral density.[5] Vitamin D3 is hydroxylated in the liver into 25-hydroxyvitamin D (calcifediol), before being processed further by the kidneys, converting it to its bioactive form (calcitriol).[5] Levels of the calcitriol precursor 25-hydroxyvitamin D (calcidiol) can be measured from blood samples and used to indicate the overall abundance of vitamin D within the body.

BPPV is a common vestibular disorder, characterised by positional vertigo and nystagmus elicited by head movements. It is caused by the displacement of small crystals of calcium carbonate known as otoconia into one of the semi-circular canals (most commonly a posterior canal). Displacement of otoconia can occur following head trauma, however most cases are idiopathic and the exact pathophysiology of BPPV currently not known.[6]

The relationship between vitamin D and BPPV is currently a popular topic of research, and a number of systematic reviews have been published in recent years indicating that BPPV incidence is associated with vitamin D deficiency [7 8], although this relationship was not supported by an earlier review [9]. There is mixed evidence regarding the relationship between vitamin D levels and BPPV recurrence, with some reviews finding lower levels of the vitamin in patients with recurrent BPPV,[9 10] whilst others have observed no significant difference in vitamin D levels and BPPV recurrence.[11] Supplementation in BPPV patients with low vitamin D levels has also been shown to result in prolonged improvement in symptoms and fewer episodes of recurrent BPPV.[12 13]

There is currently uncertainty relating to how seasonal patterns of vitamin D and BPPV incidence are related. In countries within the northern hemisphere, vitamin D levels fluctuate in a seasonal pattern- serum vitamin D levels are highest within the population of these countries during summer months and lowest during winter and spring, when there is a high prevalence of vitamin deficiency.[14 15] BPPV may also exhibit a seasonal pattern, with higher incidence rates seen between autumn and early spring,[16-18] although this has not been replicated in every study.[19 20]

This systematic review will examine the evidence concerning the relationship between serum vitamin D and BPPV for patients in northern hemisphere countries, the first time to the authors' knowledge that a review on this topic has been restricted by geographic location.

For peer review only - http://bmjopen.bmj.com/site/about/guidelines.xhtml

Methods

This systematic review was conducted following the methodology of Higgins *et al.*[21] as closely as possible, and was reported following the Preferred Reporting Items for Systematic reviews and Meta-Analyses (PRISMA). [22] Ethics approval was not necessary for this study as no new data were collected and no patient-identifiable data included.

Patient and public involvement. No patient involved.

Data sources and search strategy

A systematic literature search was performed in PUBMED, SCOPUS and Web of Science databases relating to the association between vitamin D and benign paroxysmal positional vertigo (BPPV). Date was restricted to include studies published in English between 2000 and 2023. Three searches were run through each database to account for differing possible terms for BPPV, making up a total of 12 searches which were completed on the 18th of February 2023. Search criteria and results are described in Appendix Table 1. Microsoft Excel software (Microsoft Office for Windows, version 2204) was used to store and process search results. A total of 1113 results were initially identified. Once duplicate entries were removed, the remaining 350 entries were processed further.

Studies published before the year 2000 and those pertaining to animal research were removed, then any entries that did not contain keywords "BPPV", "Vertigo", "Vitamin D" or "25-hydroxyvitamin D" within their title or abstract were excluded. Entries that were not experimental in design e.g., case reports, letters and editorials were also removed, leaving a total of 68 full texts to be manually reviewed. The included articles were reviewed independently by two reviewers for inclusion evaluation. Any inconsistencies between the two reviewers were evaluated further by both reviewers until a consensus was reached.

Inclusion and Exclusion criteria

In order to better account for seasonal differences in serum vitamin D levels, the geographical location of study participants was limited to the Northern hemisphere. All studies were available with English full texts that investigated the association between serum 25-hydroxyvitamin D levels in adult patients diagnosed with at least one episode of benign paroxysmal positional vertigo (BPPV) were included. Studies were excluded if participants had past medical history of ENT comorbidities or head trauma or were taking vitamin D supplements at the start of the study.

The literature search was undertaken by HW. Study selection was independently undertaken by the HW and GB, with inconsistencies resolved through discussion between the reviewers. Studies were deemed eligible for inclusion if they met the following criteria: 1) participants were over the age of 18 with at least one episode of diagnosed BPPV 2) participants resided in countries in the Northern hemisphere; 3) serum 25-hydroxyvitamin D levels were measured and reported; 3) participants had no past medical history of head trauma or ENT comorbidities.

Articles were excluded according to the following criteria: 1) Study did not contain reference to "BPPV", "vertigo" or "vitamin D" in title or abstract; 2) participants were taking vitamin D supplements prior to commencement of the study; 3) The article did not pertain to a research study (reviews, editorials, case studies). During application of inclusion and exclusion criteria, the decision was taken to exclude articles which included only participants with BPPV and pre-existing vitamin D deficiency as these would not be able to be utilised in subsequent analysis of the relationship between BPPV disease state and serum vitamin levels.

For peer review only - http://bmjopen.bmj.com/site/about/guidelines.xhtml

Assessment of bias

Following the application of the above inclusion and exclusion criteria, 35 studies were selected and classified according to type of study which consisted of 26 case-control and 9 cohort studies. Microsoft Excel and Microsoft Word software (Microsoft Office for Windows, version 2204) was used by the reviewers to record assessment of bias and document comments.

Assessment of bias was performed by two independent reviewers and any inconsistencies resolved by discussion between the reviewers. The case-control studies were assessed using the Risk of Bias Assessment Tool for Nonrandomized Studies (RoBANS)[23] which examines selection of participants, confounding variables, intervention measurement, blinding of outcome assessment, intervention measurement, incomplete outcome data and selective outcome reporting. The cohort studies were assessed according to the Newcastle Ottawa Scale (NOS),[24] as recommended by the Cochrane Collaboration [21]. The NOS examines participant selection, comparability and outcome and assigns studies as being of 'poor', 'fair' or 'good' quality.

Data Extraction

Data extraction was completed by HW and double checked by GB. Extracted data included: study design, author, year published, location of study participants, mean age of participants, range of participant age, sample size, percentage male participants, serum vitamin D levels for participants and the season study was performed (where reported). Standardised mean difference (SMD) with a 95% confidence interval was used to calculate the level of vitamin D in case and control groups. Based on recommendations from the clinical practice guidelines of the Endocrine Society Task Force on Vitamin D, plasma vitamin D levels of less than 20ng/ml were considered to indicate vitamin deficiency.[25] Microsoft Excel software (Microsoft Office for Windows, version 2204) was used to tabulate the extracted data.

Synthesis methods

19 articles were included in the meta-analysis of BBPV incidence and serum vitamin D; whilst 7 studies were included in meta-analysis of BPPV recurrence and serum vitamin D (See Appendix Tables 2 and 3 for data extracted from included studies). Studies were included if serum 25-hydroxyvitamin D levels (mean and standard deviation; ng/ml) were reported for cases with BPPV and controls with no medical history of BPPV. Where vitamin D levels were reported for subgroups within a study (for example male and female means reported separately), a population mean and standard deviation for vitamin D levels for both cases and controls was calculated according to the recommendations of the Cochrane Collaboration.[21]

The I^2 statistic was used to examine heterogeneity between studies, with I^2 >40% regarded as representing significant heterogeneity. The random-effect model was employed for the meta-analysis if I^2 >40%, otherwise the fixed-effect model was used instead. Statistical analysis was completed and forest plots generated using Review Manager (RevMan) software version 5.4.1. (64 bit; The Cochrane Collaboration, 2020).

Results

Study selection

The initial searches identified 1113 results comprising 350 individual articles (**Figure 1**). 282 of these were excluded; 163 articles were excluded because they did not contain reference to "BPPV" or

For peer review only - http://bmjopen.bmj.com/site/about/guidelines.xhtml

"vertigo" within their title or abstract, 61 were removed as they were not experimental (review articles, conference posters, editorials etc.), 27 did not contain "vitamin D" or related terms in their title or abstract, 6 were excluded as they related to animal studies and 25 studies were removed as they were erroneously included in search results despite being published before the year 2000.

The full text of the remaining 68 articles was reviewed for inclusion evaluation; eight articles were excluded as the participants were receiving vitamin D supplementation before commencement of the study; two were excluded as they included patients with past medical history of head trauma or ENT-related comorbidities; five were excluded as serum vitamin D levels were not measured or reported; two were excluded as the patients resided in countries in the Southern hemisphere; seven because they included participants under the age of 18 and a further five were excluded as they described patients with causes of vertigo other than BPPV. Four articles were also excluded as they included only participants with both BPPV and pre-existing vitamin D deficiency, precluding analysis of the relationship between disease state and serum vitamin levels. A manual search of the reference lists of these studies did not yield any additional articles to include in the review. A total of 35 articles underwent full-length review.

Quality assessment

The articles assessed with the RoBANS tool are described in Appendix Figure 1. All 26 of the studies were found to have a low risk of bias in three of the six domains assessed ("intervention measurement", "incomplete outcome data" and "selective outcome reporting" domains). 14 of the studies had an uncertain risk of bias in either or both of the "selection of participant" and "confounding variables" domains. All but two of the studies had a high risk of bias in the domain relating to "blinding of outcome assessment", four had a high risk of bias relating to "confounding variables" and one article had a high risk of bias in the "selection of participants" domain. None of the articles was judged to have a low risk of bias in all six domains.

The cohort studies assessed using the Newcastle Ottawa scale (NoS) ranged in quality from 'poor' to 'good' and are described in Appendix Figure 2. There were significant limitations using the NoS for some of the studies, meaning that one of the eight domains of the tool ("adequacy of follow-up of cohorts") was not applicable for five of the nine studies assessed by this tool. One study was determined to be of 'poor' quality, two 'fair' quality and the remaining six studies were 'good' quality. High risk of bias was most commonly seen in the "outcome of interest not present at start domain", and was present in seven out of nine studies. High risk of bias was seen in two studies in the "representativeness of exposed cohort" domain and in one study for the "follow-up long enough" domain. A single study was found to have a low risk of bias in all eight domains.

Description of included studies

Table 1 describes the characteristics of the included studies investigating the association between serum vitamin D and BPPV for patients within northern hemisphere countries. The 35 studies included 9,843 participants in total: 26 were case-control studies and 9 were cohort studies; there were no randomised controlled trials. Sample size varied from 10 to 3505 individuals, sex distribution from 0 to 100% male and age ranged between 18 to 89 years. Most studies were conducted in countries situated in Asia (twelve in China, six in Turkey, four in the Republic of Korea, two in India, and one each in Japan, Kuwait and Thailand); six studies in Europe (three in Italy and one each in Croatia, Germany and Portugal); one study was conducted in Africa (Egypt) and one in North America (USA).

A total of 12 studies reported that compared with controls, patients with BPPV have lower average serum vitamin D levels.[1 26-35] Seven studies found no significant difference between the serum vitamin

For peer review only - http://bmjopen.bmj.com/site/about/guidelines.xhtml

D levels of BPPV patients and controls.[36-42] Amongst cases and controls with osteoporosis and osteopenia, two studies[2 43] found no significant difference between cases and controls, although osteoporosis was correlated with low serum vitamin D in BPPV patients.[2] Serum vitamin D levels were also reported to be lower in recovering BPPV patients with residual dizziness, compared to those without.[44]

The relationship between BPPV recurrence and serum 25-hydroxyvitamin D was examined in 12 studies, with nine studies reporting lower serum vitamin D levels in patients with recurrent BPPV compared to those experiencing an initial attack[1 31 35-37 42 45 46] and three reporting no significant difference in vitamin D levels between the two groups.[38 47 48] Three studies[41 49 50] reported that vitamin D supplementation reduced the number of recurrent episodes of BPPV. Vitamin D supplementation was seen to improve the bone mineral density of BPPV patients in one study, however the effects on BPPV recurrence were not reported.[51]

Whether vitamin D levels differed between sub-types of BPPV was assessed in four studies, two of which found that serum 25-hydroxyvitamin D were lower in canalithiasis compared to cupulolithiasis[47 52] whilst the remaining two found no significant difference in vitamin levels between BPPV sub-types.[29 53] One study reported a much higher prevalence of vitamin D deficiency (<20ng/ml) in patients undergoing an acute attack of BPPV compared with 6 months post-episode (93.5% and 38.7% respectively).[54]

Seasonality and BPPV was examined by two included studies; Shu and colleagues[16] reported that the median number of BPPV referrals was higher in winter compared to summer months, whilst Califano *et al.*[36] found no seasonal patterns of BPPV cases and high levels of vitamin D deficiency and insufficiency in both winter and summer.

Meta-analysis

A total of 19 studies reported mean and standard deviation for serum 25-hydroxyvitamin D levels in case and control groups and were included in the meta-analysis. These described a total of 7387 participants (2325 cases; 5062 controls), ranging in age from 18 to 88 years and sex distribution 0 to 100% male. Seven studies were excluded for the following reasons: four because the case and control groups both had BPPV, two as standard deviation was not reported and one as vitamin D levels were not given in ng/mL. Combined mean averages and standard deviations were calculated for cases and controls using data provided in two studies as these were initially reported according to groups (age, sex, BPPV recurrence). Pooled standardised mean difference (SMD) indicated that serum vitamin D is not significantly different in cases with BPPV compared to controls (SMD -1.90; 95% confidence interval -4.18, 0.38; p = 0.1 random effects model; **Figure 2**). There was substantial heterogeneity across included studies (I^2 = 99%; P < 0.00001).

Seven studies reported mean and standard deviation for serum 25-hydroxyvitamin D levels in patient groups with recurrent and non-recurrent BPPV and were included in the meta-analysis. These described a total of 615 participants (248 cases; 367 controls), ranging in age from 20 to 89 years and sex distribution from 0 to 35% male. Eight studies were excluded from the meta-analysis for the following reasons: three because no standard deviation was reported, three because serum vitamin D levels were not reported and two because vitamin D supplements were utilised. Pooled standardised mean difference (SMD) indicated that serum vitamin D is no different in individuals with recurrent BPPV compared to those with non-recurrent disease (SMD -1.92; 95% confidence interval -3.85 - -0.02; p = 0.05 random effects model; **Figure 3**). There was moderate heterogeneity across included studies (I^2 = 66%; P < 0.01).

For peer review only - http://bmjopen.bmj.com/site/about/guidelines.xhtml

***Table 1*. Characteristics of included studies.** NOS = Newcastle Ottawa Scale (Wells et al 2018).

Author and year	Sample size	Study design	Country	Age range	Case Mean age (SD)	Case % male	Case 25(OH)D (ng/ml) mean (SD)	Quality
Bi *et al.* 2021	52 (27 case, 25 control)	Prospective Case-control	China	not reported	51.6 ± 15.8	33.33	14.64 ± 6.94*	n/a RoBANS
Califano *et al.* 2019	227 (127 case, 100 control)	Retro- + Pro-spective Case-control	Italy	not reported	60 ± 11.5**	40.2	24.4 ± 10.69	n/a RoBANS
Carneiro de Sousa *et al.* 2019	10 (5 case, 5 control)	Prospective Case-control	Portugal	52-82	64 ± 9.3 (case + control)	0	13.6 ± 5.7**	n/a RoBANS
Çelik *et al.* 2021	339 (190 case, 149 control)	Prospective Case-control	Turkey	not reported	43.7 ± 16	57.4	15.64 ± 8.4*	n/a RoBANS
Ceylan and Kanmaz 2020	197 (97 case, 100 control)	Retrospective Case-control	Turkey	not reported	57.07 ± 12.6	34	15.87 ± 8.6*	n/a RoBANS
Cheng et al. 2021	640 (320 case, 320 control	Prospective Case-control	China	not reported	68.2±6.02	45	23.2±4.09	n/a RoBANS
Çıkrıkçı Işık *et al.* 2017	127 (64 case, 63 control)	Retrospective Case-control	Turkey	not reported	56 ± 13.1	26.6	9.51 ± 5.49*	n/a RoBANS
Ding *et al.* 2019	522 (174 case, 348 control)	Prospective Case-control	China	not reported	Median reported	41.4	18.2 (n/a)*	n/a RoBANS
Elmoursey and Abbas 2021	60	Prospective Case-control	Egypt	24-70	46 ± 12.6	40	26.1 ± 11.7	n/a RoBANS
Goldschagg *et al.* 2021	459 (158 cases, 301 control)	Retrospective Case-control	Germany	not reported	61 ± 14	44.9	23.4 ± 9.4	n/a RoBANS
Gu, Dong and Gu 2018	100 (50 case, 50 control)	Prospective Case-control	China	not reported	51.36 ± 10.58	40	17.62 ± 9.0	n/a RoBANS
Han *et al.* 2018	165 (85 case, 80 control)	Retrospective Case-control	China	44-88	63.5 ± 9.72	0	19.1 ± 5.2	n/a RoBANS
Han *et al.* 2020	117	Retrospective Cohort	Republic of Korea	25-78	55 ± 11	22.2	24.47 ± 11.6**	Good
Han *et al.* 2021	201	Retrospective Cohort	China	not reported	65.14 ± 13.35**	0	19.74 ± 6.4**	Fair
Inan *et al.* 2021	104 (52 case, 52 control)	Retrospective Case-control	Turkey	18-80	55.6 (n/a)	44	15.3 ± 9.8	n/a RoBANS
Kahraman *et al.* 2016	37	Retrospective Cohort	Turkey	23–75	49.8 (n/a)	35.5	9.73 ± 8.8 (initial visit)*	Good
Karataş et al 2017	156 (78 case, 78 control)	Retrospective Case-control	Turkey	22-85	51.4 ± 12.2	37.2	23.0 ± 14.4	n/a RoBANS
Lee *et al.* 2017	132	Retrospective Cohort	Republic of Korea	49-81	63 ± 10.0	0	29.43 ± 14.2**	Fair
Maslovara *et al.* 2018	40	Prospective Cohort	Croatia	not reported	64.2 ± 12.1	28	21 ± 7.4	Good
Melis *et al.* 2020	73	Prospective Cohort	Italy	not reported	62.99 ± 14.6**	27.4**	22.13 ± 15.1**	Good
Nakada *et al.* 2019	35	Retrospective Cohort	Japan	not reported	76.36 ± 6.4**	34.3**	16.29 ± 3.9**	Poor
Parham *et al.* 2013	29 (16 case, 13 control)	Prospective Case-Control	USA	49-81	68.5 (n/a)	0	reported as nM/L	n/a RoBANS
Pecci *et al.* 2022	50 (26 case, 24 control)	Prospective Case-control	Italy	39-79	61.85 (n/a)	23	20.18 (SD not given)	n/a RoBANS
Sarsitthithim *et al.* 2021	137 (69 case, 68 control)	Prospective Case-Control	Thailand	39-89	61.4 ± 11.5	14.5	21.5 ± 5.3	n/a RoBANS
Sen, Padiyar and Arora 2018	200 (100 case, 100 control)	Prospective Case-control	India	18-60	48.6 ± 10.2	33	20.3 ± 12.2	n/a RoBANS
Shu *et al.* 2019	877	Retrospective Cohort	China	not reported	Median reported	not reported	18.03 (n/a)**	n/a RoBANS
Shin *et al.* 2023	50	Retrospective Cohort	Republic of Korea	not reported	50.5 ± 14.6**	28**	Reported by group	Good
Song *et al.* 2020	3505 (380 case, 3125 control)	Prospective Case-Control	China	19-85	50.7 ± 13.6**	25.5	14.24 ± 6.6**	Good
Talaat *et al.* 2015	180 (80 case, 100 control)	Prospective Case-Control	Kuwait	31-71	47.6 ± 9.1	35	14.19 ± 9.3**	n/a RoBANS
Thomas *et al.* 2021	98 (49 cases, 49 controls)	Prospective Case-control	India	21-71	44.39 (n/a)	32.7	21.3 ± 0.57	n/a RoBANS
Wang *et al.* 2020	183 (103 case, 80 control)	Prospective Case-Control	China	50-86	63.0 ± 9.3	0	17.2 ± 2.0	n/a RoBANS
Wu *et al.* 2018	152 (60 case, 92 control)	Retrospective Case-control	China	not reported	59.35 ± 13.24	100	23.0 ± 6.8	n/a RoBANS
Wu *et al.* 2022	123 (51 case; 72 control)	Prospective Case-control	China	not reported	58.5 ± 12.6**	32.5	Median reported	n/a RoBANS
Yang *et al.* 2017	260 (130 case, 130 control)	Retrospective Case-control	Republic of Korea	20-87	54.9 ± 12.2	23.1	18.2 ± 10.3	n/a RoBANS
Zhang *et al.* 2022	206 (156 case, 50 control)	Prospective Case-control	China	46-75	59.5 ± 7.4	0	18.8 ± 2.5**	n/a RoBANS

Abbreviations: 25(OH)D = 25hydroxyvitamin D, BPPV= benign paroxysmal positional vertigo, SD= standard deviation, *= vitamin deficiency (≤20 ng/ml)

For peer review only - http://bmjopen.bmj.com/site/about/guidelines.xhtml

** Calculated combined average/SD. Combines groups of means and Standard Deviations into a Single Group by Repeating Cochrane's formula

For peer review only

For peer review only - http://bmjopen.bmj.com/site/about/guidelines.xhtml

Discussion

Our systematic review and meta-analyses were based on a total of 35 articles; 26 case-control studies and nine cohort studies. It provides up-to-date information regarding the evidence base for vitamin D and benign paroxysmal positional vertigo (BPPV). The included studies described 9843 participants, of which 8002 were included in the meta-analysis. The studies included a broad range of populations from thirteen different countries in the Northern Hemisphere. Study quality was variable, and the majority of included studies were identified as having a high risk of bias in at least one domain after application of the NOS or ROBANS tools.

The main findings of the meta-analysis were that although a negative trend was identified indicating that individuals with BPPV may have lower average serum 25-hydroxyvitamin D levels compared to controls, this was found to be statistically non-significant (P = 0.1). Examining the published literature to date, it is clear that the evidence base regarding the link between vitamin D and BPPV is mixed- with a number of studies included in this review reporting no significant difference between cases with BPPV and controls and others reporting lower serum vitamin D levels in patients with BPPV. These mixed results may reflect that the strength of the relationship between vitamin D and BPPV is relatively weak. Another possibility is that rather than vitamin D having a direct physiological role in the development of BPPV it is an indirect marker of another related biomarker, for example calcium; as calcitriol, manufactured from 25-hydroxyvitamin D, plays a role in regulating intestinal absorption of dietary calcium and bone mineralisation both directly and through interaction with parathyroid hormone.[55]

Whilst the effect size in our meta-analysis was relatively small (SMD -1.90), and not seen to be statistically significant, its small effect size aligns with other non-geographically restricted systematic reviews performed previously which have established an association between low serum vitamin D and BPPV incidence.[7 8] It remains unclear however, whether the small absolute difference in serum vitamin D concentration reported between patients with BPPV and those without is physiologically relevant.

The results of our meta-analysis into the association between BPPV recurrence and serum vitamin D levels indicated a similar (SMD -1.92), also non-significant association between BPPV recurrence and lower serum vitamin D levels. Previously published, non-geographically restricted systematic reviews have demonstrated a mixed picture relating to the relationship between serum vitamin D and BPPV recurrence with both a negative association[8 56], or no impact on recurrence[11] reported.

It might have been expected that restricting our review to include only those conducted in the northern hemisphere would result in a larger effect size, as more northern latitude has previously been shown to be a risk factor for vitamin D deficiency.[57 58] However, as vitamin D deficiency is more prevalent in such countries during the winter,[59] this may have been confounded by the fact that most of the included studies did not include temporal data relating to the month and/or seasons when vitamin D levels were measured or BPPV occurred. Some of the articles included in this review indicated mixed evidence for a potential relationship between serum vitamin D levels and sub-types of BPPV. Two articles[47 52] found lower serum vitamin D levels in canalithiasis compared to cupulolithiasis, whilst a further two found no significant difference in vitamin D levels between the BPPV sub-types.[29 53] It was not possible to perform a meta-analysis on this effect due to the small number of studies investigating this relationship, however this could be a parameter for future studies investigating BPPV and vitamin D to investigate further. Similarly, the potential association between BPPV, vitamin D and bone mineral density identified by some of the articles[2 43] could not be further analysed with meta-analysis but could be an area of interest for future research.

For peer review only - http://bmjopen.bmj.com/site/about/guidelines.xhtml

It was not possible to perform a meta-analysis examining the seasonality of both BPPV and vitamin D as only two studies reported vitamin D levels according to month or season.[16 36] This is an important potential confounding factor, as vitamin D levels have been observed to be at their lowest during months when the incidence of BPPV is greatest.[18] One hypothesis is that higher levels of vitamin D may offer a protective effect against BPPV during the summer months, and that the decrease in vitamin D levels over the winter months results in the increased incidence of BPPV seen during these months. Future research should separate individuals by month or season of admission (summer and winter-spring) and examine the difference in serum vitamin D levels between patients and controls during these time periods.

In conclusion, our results indicate that low serum vitamin D levels are not correlated with incidence nor recurrence of BPPV. It was not possible to further investigate the seasonal relationships of BPPV and vitamin D deficiency as most studies do not include data relating to when patients are seen, and this is an area of interest for future research. Investigating if serum vitamin D levels relate to sub-types of BPPV and whether restoring vitamin D levels to sufficient levels via supplementation offers protection against BPPV are other topics which would benefit from further studies. Despite the lack of association between BPPV and serum vitamin D, as a large proportion of the population of countries in northern hemisphere countries are at a greater risk of vitamin D deficiency, patients developing BPPV in these countries may benefit from opportunistic serum vitamin D testing and/or supplementation.

For peer review only

For peer review only - http://bmjopen.bmj.com/site/about/guidelines.xhtml

Figure Legends:

Figure 1. *Flow diagram detailing results of searches and study selection process*

Figure 2. Relationship between serum 25-hydroxyvitamin D and Benign Paroxysmal Positional Vertigo incidence.

Figure 3. Relationship between serum 25-hydroxyvitamin D and Benign Paroxysmal Positional Vertigo recurrence.

For peer review only

For peer review only - http://bmjopen.bmj.com/site/about/guidelines.xhtml

Funding: This research received no specific grant from any funding agency in the public, commercial or not-for-profit sectors. KK was supported by the NIHR Manchester Biomedical Research Centre, UK IS-BRC-1215-20007, NIHR203308 and MRC Programme Grants MR/L003589/1, MR/V01272X/1.

Competing interests: None to declare

Data Sharing: No additional data.

For peer review only

References

1. Talaat HS, Abuhadied G, Talaat AS, et al. Low bone mineral density and vitamin D deficiency in patients with benign positional paroxysmal vertigo. *European archives of oto-rhino-laryngology : official journal of the European Federation of Oto-Rhino-Laryngological Societies (EUFOS) : affiliated with the German Society for Oto-Rhino-Laryngology - Head and Neck Surgery* 2015;272(9):2249-53. doi: 10.1007/s00405-014-3175-3 [published Online First: 2014/06/30]
2. Lee SB, Lee CH, Kim YJ, et al. Biochemical markers of bone turnover in benign paroxysmal positional vertigo. *PloS one* 2017;12(5):e0176011. doi: 10.1371/journal.pone.0176011 [published Online First: 2017/05/04]
3. Büki B, Jünger H, Zhang Y, et al. The Price of Immune Responses and the Role of Vitamin D in the Inner Ear. *Otology & neurotology : official publication of the American Otological Society, American Neurotology Society [and] European Academy of Otology and Neurotology* 2019;40(6):701-09. doi: 10.1097/mao.0000000000002258 [published Online First: 2019/06/14]
4. Borel P, Caillaud D, Cano NJ. Vitamin D Bioavailability: State of the Art. *Critical Reviews in Food Science and Nutrition* 2015;55(9):1193-205. doi: 10.1080/10408398.2012.688897
5. Alshahrani F, Aljohani N. Vitamin D: deficiency, sufficiency and toxicity. *Nutrients* 2013;5(9):3605-16. doi: 10.3390/nu5093605 [published Online First: 20130913]
6. You P, Instrum R, Parnes L. Benign paroxysmal positional vertigo. *Laryngoscope investigative otolaryngology* 2019;4(1):116-23. doi: 10.1002/lio2.230 [published Online First: 2019/03/05]
7. Chen J, Zhao W, Yue X, et al. Risk Factors for the Occurrence of Benign Paroxysmal Positional Vertigo: A Systematic Review and Meta-Analysis. *Frontiers in neurology* 2020;11:506. doi: 10.3389/fneur.2020.00506 [published Online First: 2020/07/14]
8. Yang B, Lu Y, Xing D, et al. Association between serum vitamin D levels and benign paroxysmal positional vertigo: a systematic review and meta-analysis of observational studies. *European archives of oto-rhino-laryngology : official journal of the European Federation of Oto-Rhino-Laryngological Societies (EUFOS) : affiliated with the German Society for Oto-Rhino-Laryngology - Head and Neck Surgery* 2020;277(1):169-77. doi: 10.1007/s00405-019-05694-0 [published Online First: 2019/10/21]
9. AlGarni MA, Mirza AA, Althobaiti AA, et al. Association of benign paroxysmal positional vertigo with vitamin D deficiency: a systematic review and meta-analysis. *European archives of oto-rhino-laryngology : official journal of the European Federation of Oto-Rhino-Laryngological Societies (EUFOS) : affiliated with the German Society for Oto-Rhino-Laryngology - Head and Neck Surgery* 2018;275(11):2705-11. doi: 10.1007/s00405-018-5146-6 [published Online First: 2018/10/12]
10. Chen J, Zhang S, Cui K, et al. Risk factors for benign paroxysmal positional vertigo recurrence: a systematic review and meta-analysis. *Journal of neurology* 2020 doi: 10.1007/s00415-020-10175-0 [published Online First: 2020/08/26]
11. Li S, Wang Z, Liu Y, et al. Risk Factors for the Recurrence of Benign Paroxysmal Positional Vertigo: A Systematic Review and Meta-Analysis. *Ear, nose, & throat journal* 2022;101(3):NP112-NP34. doi: 10.1177/0145561320943362 [published Online First: 2020/08/11]
12. Sheikhzadeh M, Lotfi Y, Mousavi A, et al. Influence of supplemental vitamin D on intensity of benign paroxysmal positional vertigo: A longitudinal clinical study. *Caspian journal of internal medicine* 2016;7(2):93-8. [published Online First: 2016/07/08]
13. Yang Z, Li J, Zhu Z, et al. Effect of vitamin D supplementation on benign paroxysmal positional vertigo recurrence: A meta-analysis. *Science progress* 2021;104(2):368504211024569. doi: 10.1177/00368504211024569 [published Online First: 2021/06/16]
14. Cashman KD, Dowling KG, Škrabáková Z, et al. Vitamin D deficiency in Europe: pandemic? *The American journal of clinical nutrition* 2016;103(4):1033-44. doi: 10.3945/ajcn.115.120873 [published Online First: 2016/02/13]
15. Manios Y, Moschonis G, Lambrinou CP, et al. A systematic review of vitamin D status in southern European countries. *European journal of nutrition* 2018;57(6):2001-36. doi: 10.1007/s00394-017-1564-2 [published Online First: 2017/11/02]
16. Shu L, Wu J, Jiang CY, et al. Seasonal variation of idiopathic benign paroxysmal positional vertigo correlates with serum 25-hydroxyvitamin D levels: a six-year registry study in Shanghai, China. *Scientific reports* 2019;9(1):16230. doi: 10.1038/s41598-019-52803-4 [published Online First: 2019/11/09]
17. Sideris G, Sapountzi MC, Malamas V, et al. Climatic Variations as Indicators of Vitamin D Levels and Benign Paroxysmal Positional Vertigo. *Cureus* 2021;13(10):e18811. doi: 10.7759/cureus.18811 [published Online First: 2021/11/23]
18. Whitman GT, Baloh RW. Seasonality of benign paroxysmal positional vertigo. *JAMA otolaryngology-- head & neck surgery* 2015;141(2):188-9. doi: 10.1001/jamaoto.2014.2941 [published Online First: 2014/11/21]
19. Jeong J, Eo TS, Oh J, et al. Monthly and seasonal variations in benign paroxysmal positional vertigo. *Journal of vestibular research : equilibrium & orientation* 2021;31(2):101-07. doi: 10.3233/ves-200030 [published Online First: 2021/02/09]
20. Seidel DU, Park JJ, Sesterhenn AM, et al. Demographic data and seasonal variation in peripheral vestibular disorders in ENT practices in Germany. *Journal of vestibular research : equilibrium & orientation* 2019;29(4):181-90. doi: 10.3233/ves-190668 [published Online First: 2019/07/01]
21. Higgins JPT, Altman DG, Gøtzsche PC, et al. The Cochrane Collaboration’s tool for assessing risk of bias in randomised trials. *BMJ* 2011;343

For peer review only - http://bmjopen.bmj.com/site/about/guidelines.xhtml

22. Page MJ, McKenzie JE, Bossuyt PM, et al. The PRISMA 2020 statement: an updated guideline for reporting systematic reviews. *BMJ* 2021;372:n71. doi: 10.1136/bmj.n71
23. Kim SY, Park JE, Lee YJ, et al. Testing a tool for assessing the risk of bias for nonrandomized studies showed moderate reliability and promising validity. *Journal of clinical epidemiology* 2013;66(4):408-14. doi: 10.1016/j.jclinepi.2012.09.016 [published Online First: 2013/01/23]
24. Wells GA, Shea B, O'Connell D, et al. The Newcastle-Ottawa Scale (NOS) for assessing the quality of nonrandomised studies in meta-analyses 2018 [Available from: http://www.ohri.ca/programs/clinical_epidemiology/oxford.asp accessed 21.05.2021 2021.
25. Holick MF. Vitamin D deficiency. *The New England journal of medicine* 2007;357(3):266-81. doi: 10.1056/NEJMra070553 [published Online First: 2007/07/20]
26. Bi J, Liu B, Zhang Y, et al. Study on the Bone Metabolism Indices and Otoconin-90 in Benign Paroxysmal Positional Vertigo. *Otology & neurotology : official publication of the American Otological Society, American Neurotology Society [and] European Academy of Otology and Neurotology* 2021;42(6):e744-e49. doi: 10.1097/mao.0000000000003087 [published Online First: 2021/02/20]
27. Çelik H, Yardim A, Ertaş A, et al. Evaluation of Serum 25-Hydroxyvitamin Vitamin D, Vitamin B12, and Folate Levels in Patients with Benign Paroxysmal Positional Vertigo. . *Eurasian Journal of Emergency Medicine* 2021;20(1):19-24. doi: 10.4274/eajem.galenos.2020.88942
28. Ding J, Liu L, Kong WK, et al. Serum levels of 25-hydroxy vitamin D correlate with idiopathic benign paroxysmal positional vertigo. *Bioscience reports* 2019;39(4) doi: 10.1042/bsr20190142 [published Online First: 2019/04/10]
29. Han K, Yun YM, Moon SG, et al. Bone mineral density and serum 25-hydroxyvitamin D in subtypes of idiopathic benign paroxysmal positional vertigo. *American journal of otolaryngology* 2020;41(1):102313. doi: 10.1016/j.amjoto.2019.102313 [published Online First: 2019/11/17]
30. Inan HC, Mertoğlu C, Erdur ZB. Investigation of Serum Calcium and 25-Hydroxy Vitamin D Levels in Benign Paroxysmal Positional Vertigo Patients. *Ear, nose, & throat journal* 2021;100(9):643-46. doi: 10.1177/0145561321989451 [published Online First: 2021/01/26]
31. Sarsitthithum K, Wisupagan T, Kiatthanabumrung S, et al. The Association Between Serum Vitamin D Levels and Benign Paroxysmal Positional Vertigo. *Ear, nose, & throat journal* 2021:1455613211008561. doi: 10.1177/01455613211008561 [published Online First: 2021/04/20]
32. Sen K, Padiyar BV, Arora G. Association of Benign Paroxysmal Positional Vertigo with Osteoporosis and Vitamin D Deficiency – A Case-Control Study. *Dubai Medical Journal* 2018;1:2-5. doi: 10.1159/000492756
33. Wang Z, Yao G, Tao X, et al. Evaluation of bone mineral density and 25-(OH) vitamin D levels in middle-aged and elderly women with recurrent benign paroxysmal positional vertigo. *Acta oto-laryngologica* 2020;140(2):89-93. doi: 10.1080/00016489.2019.1692146 [published Online First: 2019/12/27]
34. Wu Y, Fan Z, Jin H, et al. Assessment of Bone Metabolism in Male Patients With Benign Paroxysmal Positional Vertigo. *Frontiers in neurology* 2018;9:742. doi: 10.3389/fneur.2018.00742 [published Online First: 2018/09/21]
35. Zhang X, Zhang Z, Lv X. Predictive values of serum estradiol, calcium, and 25-hydroxyvitamin D levels for recurrence of benign paroxysmal positional vertigo in postmenopausal women. *Turk J Phys Med Rehabil* 2022;68(1):30-36. doi: 10.5606/tftrd.2022.5964 [published Online First: 20220301]
36. Califano L, Salafia F, Melillo MG, et al. Is hypovitaminosis D a risk factor for either the onset or the recurrence of Benign Paroxysmal Positional Vertigo? . *Frontiera ORL* 2019
37. Ceylan SM, Kanmaz MA. Vitamin D Influence on the Clinical Course of Benign Paroxysmal Positional Vertigo: A Retrospective Cohort Study. *Otorhinolaryngology Clinics: An International Journal* 2020;12(3):43-47.
38. Çikrikçi Işik G, Cevik Y, Emektar E, et al. Analysis of Vitamin D and Calcium Levels in Benign Paroxysmal Positional Vertigo. *Eurasian Journal of Emergency Medicine* 2017;16:128-32. doi: 10.5152/eajem.2017.58077
39. Goldschagg N, Teupser D, Feil K, et al. No evidence for a specific vitamin D deficit in benign paroxysmal positional vertigo. *European journal of neurology* 2021;28(9):3182-86. doi: 10.1111/ene.14980 [published Online First: 2021/06/17]
40. Karataş A, Acar Yuceant G, Yuce T, et al. Association of Benign Paroxysmal Positional Vertigo with Osteoporosis and Vitamin D Deficiency: A Case Controlled Study. *The journal of international advanced otology* 2017;13(2):259-65. doi: 10.5152/iao.2016.2640 [published Online First: 2017/03/10]
41. Pecci R, Mandalà M, Marcari A, et al. Vitamin D Insufficiency/Deficiency in Patients with Recurrent Benign Paroxysmal Positional Vertigo. *The journal of international advanced otology* 2022;18(2):158-66. doi: 10.5152/iao.2022.21269
42. Thomas RJ, Goutham MK, Bhat VS, et al. Association of Serum Calcium and Vitamin D with Benign Paroxysmal Positional Vertigo. *Int Arch Otorhinolaryngol* 2022;26(3):e365-e69. doi: 10.1055/s-0041-1724093 [published Online First: 20211103]
43. Parham K, Leonard G, Feinn RS, et al. Prospective clinical investigation of the relationship between idiopathic benign paroxysmal positional vertigo and bone turnover: a pilot study. *The Laryngoscope* 2013;123(11):2834-9. doi: 10.1002/lary.24162 [published Online First: 2013/05/16]
44. Wu Y, Han K, Han W, et al. Low 25-Hydroxyvitamin D Levels Are Associated With Residual Dizziness After Successful Treatment of Benign Paroxysmal Positional Vertigo. *Frontiers in neurology* 2022;13:915239. doi: 10.3389/fneur.2022.915239 [published Online First: 20220622]

For peer review only - http://bmjopen.bmj.com/site/about/guidelines.xhtml

45. Melis A, Rizzo D, Gallus R, et al. Relationship between calcium metabolism and benign paroxysmal positional vertigo in north Sardinia population. *Journal of vestibular research : equilibrium & orientation* 2020;30(6):375-82. doi: 10.3233/ves-200025
46. Shin HI, Park Y, Lee HJ, et al. Correlation between serum vitamin D level and benign paroxysmal positional vertigo recurrence. *Auris Nasus Larynx* 2023 doi: 10.1016/j.anl.2022.12.017 [published Online First: 20230123]
47. Maslovara S, Butkovic Soldo S, Sestak A, et al. 25 (OH) D3 levels, incidence and recurrence of different clinical forms of benig paroxysmal positional vertigo. *Brazilian journal of otorhinolaryngology* 2018;84(4):453-59. doi: 10.1016/j.bjorl.2017.05.007 [published Online First: 2017/06/29]
48. Yang CJ, Kim Y, Lee HS, et al. Bone mineral density and serum 25-hydroxyvitamin D in patients with idiopathic benign paroxysmal positional vertigo. *Journal of vestibular research : equilibrium & orientation* 2018;27(5-6):287-94. doi: 10.3233/ves-170625 [published Online First: 2018/02/06]
49. Carneiro de Sousa PJM, Abreu Pereira DM, Carneiro Melo Pereira de Magalhães P, et al. Vitamin D deficiency and benign paroxysmal positioning vertigo. *Hearing, Balance and Communication* 2019;17(2):179-81. doi: 10.1080/21695717.2019.1590988
50. Elmoursy MM, Abbas AS. The role of low levels of vitamin D as a co-factor in the relapse of benign paroxysmal positional vertigo (BPPV). *American journal of otolaryngology* 2021;42(6):103134. doi: 10.1016/j.amjoto.2021.103134 [published Online First: 2021/06/25]
51. Gu X, Dong F, Gu J. Analysis of effect of 1α-hydroxyvitamin D3 on benign paroxysmal positional vertigo and risk factors. *Experimental and therapeutic medicine* 2018;15(3):2321-26. doi: 10.3892/etm.2018.5699 [published Online First: 2018/02/20]
52. Nakada T, Sugiura S, Uchida Y, et al. Difference in Serum Levels of Vitamin D Between Canalolithiasis and Cupulolithiasis of the Horizontal Semicircular Canal in Benign Paroxysmal Positional Vertigo. *Frontiers in neurology* 2019;10:176. doi: 10.3389/fneur.2019.00176 [published Online First: 2019/03/19]
53. Han WW, Wu YQ, Fan ZY, et al. Characteristics of bone metabolism in postmenopausal female patients with different types of idiopathic benign paroxysmal positional vertigo: A single-centre retrospective study. *American journal of otolaryngology* 2021;42(6):103149. doi: 10.1016/j.amjoto.2021.103149 [published Online First: 2021/07/10]
54. Kahraman SS, Ozcan O, Arli C, et al. Calcium Homeostasis During Attack and Remission in Patients With Idiopathic Benign Paroxysmal Positional Vertigo. *Otology & neurotology : official publication of the American Otological Society, American Neurotology Society [and] European Academy of Otology and Neurotology* 2016;37(9):1388-92. doi: 10.1097/mao.0000000000001167 [published Online First: 2016/08/16]
55. Fleet JC. The role of vitamin D in the endocrinology controlling calcium homeostasis. *Molecular and cellular endocrinology* 2017;453:36-45. doi: 10.1016/j.mce.2017.04.008 [published Online First: 2017/04/13]
56. Chen J, Zhang S, Cui K, et al. Risk factors for benign paroxysmal positional vertigo recurrence: a systematic review and meta-analysis. *Journal of neurology* 2021;268(11):4117-27. doi: 10.1007/s00415-020-10175-0 [published Online First: 20200824]
57. Leary PF, Zamfirova I, Au J, et al. Effect of Latitude on Vitamin D Levels. *J Am Osteopath Assoc* 2017;117(7):433-39. doi: 10.7556/jaoa.2017.089
58. Oskarsson V, Eliasson M, Salomaa V, et al. Influence of geographical latitude on vitamin D status: cross-sectional results from the BiomarCaRE consortium. *British Journal of Nutrition* 2022;128(11):2208-18. doi: 10.1017/S0007114521005080 [published Online First: 2021/12/22]
59. Huotari A, Herzig KH. Vitamin D and living in northern latitudes--an endemic risk area for vitamin D deficiency. *International journal of circumpolar health* 2008;67(2-3):164-78. doi: 10.3402/ijch.v67i2-3.18258 [published Online First: 2008/09/05]

For peer review only - http://bmjopen.bmj.com/site/about/guidelines.xhtml

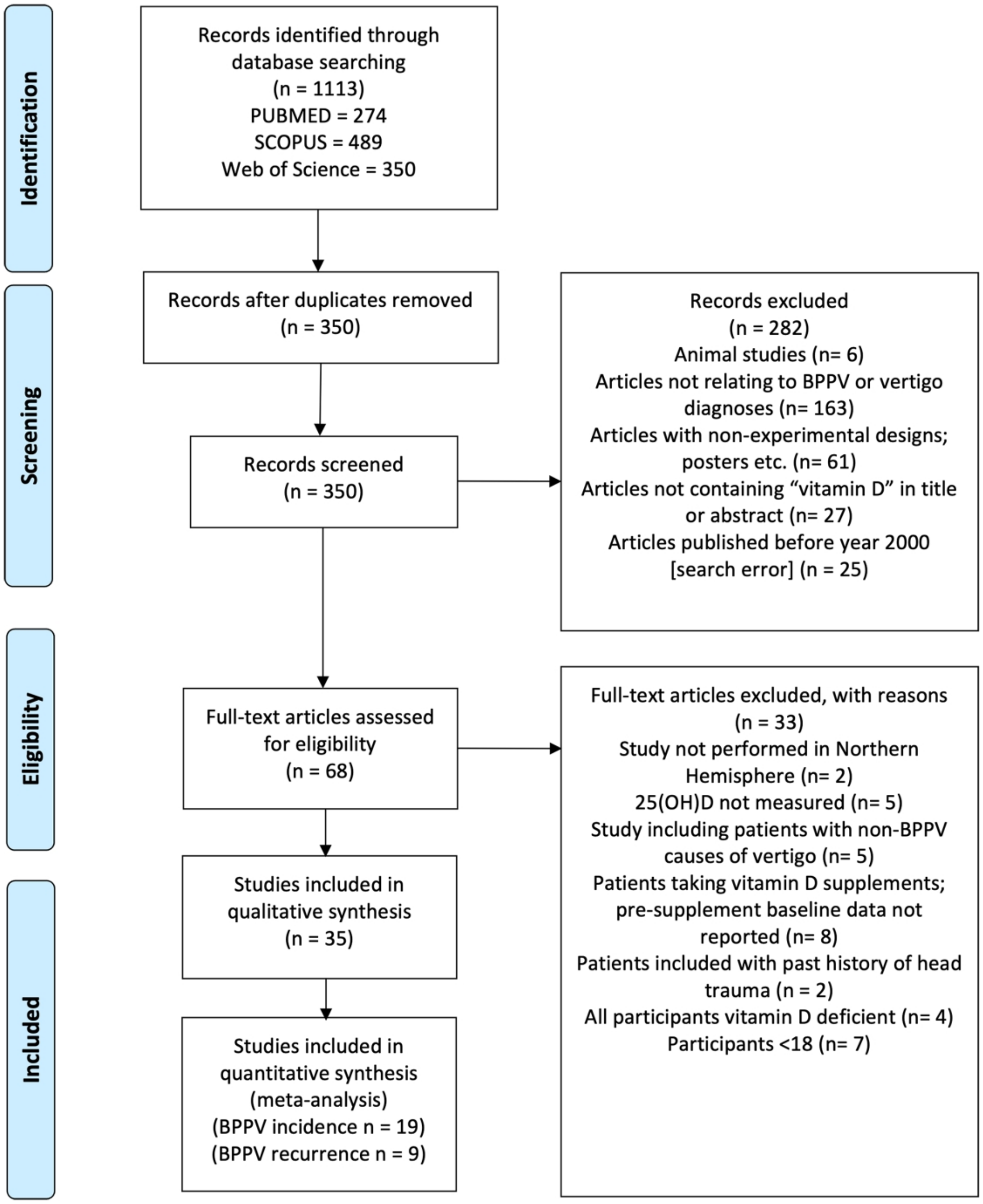

Figure 1. Flow diagram detailing results of searches and study selection process

170x208mm (300 x 300 DPI)

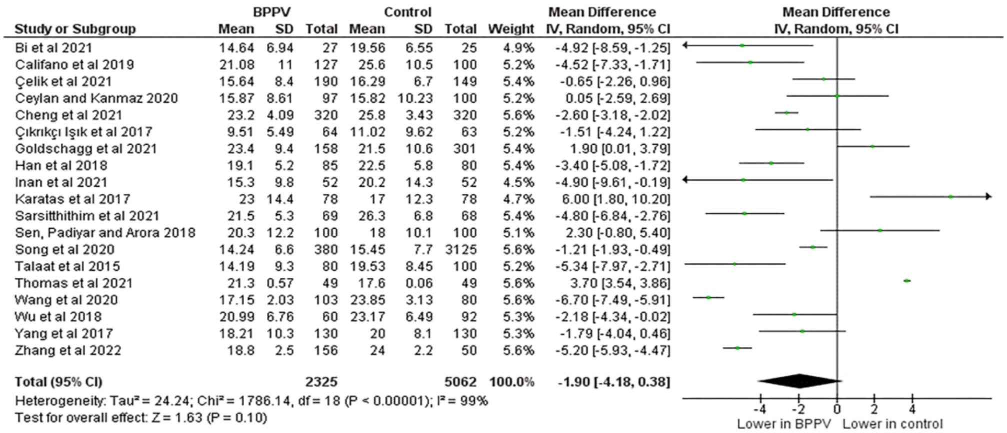

Study or Subgroup	BPPV Mean	BPPV SD	BPPV Total	Control Mean	Control SD	Control Total	Weight	Mean Difference IV, Random, 95% CI
Bi et al 2021	14.64	6.94	27	19.56	6.55	25	4.9%	-4.92 [-8.59, -1.25]
Califano et al 2019	21.08	11	127	25.6	10.5	100	5.2%	-4.52 [-7.33, -1.71]
Çelik et al 2021	15.64	8.4	190	16.29	6.7	149	5.4%	-0.65 [-2.26, 0.96]
Ceylan and Kanmaz 2020	15.87	8.61	97	15.82	10.23	100	5.2%	0.05 [-2.59, 2.69]
Cheng et al 2021	23.2	4.09	320	25.8	3.43	320	5.6%	-2.60 [-3.18, -2.02]
Çıkrıkçı Işık et al 2017	9.51	5.49	64	11.02	9.62	63	5.2%	-1.51 [-4.24, 1.22]
Goldschagg et al 2021	23.4	9.4	158	21.5	10.6	301	5.4%	1.90 [0.01, 3.79]
Han et al 2018	19.1	5.2	85	22.5	5.8	80	5.4%	-3.40 [-5.08, -1.72]
Inan et al 2021	15.3	9.8	52	20.2	14.3	52	4.5%	-4.90 [-9.61, -0.19]
Karatas et al 2017	23	14.4	78	17	12.3	78	4.7%	6.00 [1.80, 10.20]
Sarsitthithim et al 2021	21.5	5.3	69	26.3	6.8	68	5.4%	-4.80 [-6.84, -2.76]
Sen, Padiyar and Arora 2018	20.3	12.2	100	18	10.1	100	5.1%	2.30 [-0.80, 5.40]
Song et al 2020	14.24	6.6	380	15.45	7.7	3125	5.6%	-1.21 [-1.93, -0.49]
Talaat et al 2015	14.19	9.3	80	19.53	8.45	100	5.2%	-5.34 [-7.97, -2.71]
Thomas et al 2021	21.3	0.57	49	17.6	0.06	49	5.6%	3.70 [3.54, 3.86]
Wang et al 2020	17.15	2.03	103	23.85	3.13	80	5.6%	-6.70 [-7.49, -5.91]
Wu et al 2018	20.99	6.76	60	23.17	6.49	92	5.3%	-2.18 [-4.34, -0.02]
Yang et al 2017	18.21	10.3	130	20	8.1	130	5.3%	-1.79 [-4.04, 0.46]
Zhang et al 2022	18.8	2.5	156	24	2.2	50	5.6%	-5.20 [-5.93, -4.47]
**Total (95% CI)**			**2325**			**5062**	**100.0%**	**-1.90 [-4.18, 0.38]**

Heterogeneity: $Tau^2 = 24.24$; $Chi^2 = 1786.14$, df = 18 (P < 0.00001); $I^2 = 99\%$
Test for overall effect: Z = 1.63 (P = 0.10)

Figure 2. Relationship between serum 25-hydroxyvitamin D and Benign Paroxysmal Positional Vertigo incidence.

251x110mm (300 x 300 DPI)

Study or Subgroup	Recurrence Mean	SD	Total	Non-recurrence Mean	SD	Total	Weight	Mean Difference IV, Random, 95% CI
Çıkrıkçı Işık et al 2017	8.81	4.9	16	9.91	5.81	41	15.8%	-1.10 [-4.09, 1.89]
Melis et al 2020	19.53	15.33	43	25.85	14.1	30	6.1%	-6.32 [-13.14, 0.50]
Sarsitthithim et al 2021	21.9	4.9	41	21	5.9	28	17.2%	0.90 [-1.75, 3.55]
Shin et al 2023	12.9	8	19	19.2	8.2	31	10.4%	-6.30 [-10.91, -1.69]
Talaat et al 2015	11.93	7.57	36	16.04	10.26	44	12.5%	-4.11 [-8.02, -0.20]
Yang et al 2017	19.3	11.1	63	17.2	9.4	67	13.7%	2.10 [-1.45, 5.65]
Zhang et al 2022	16.7	2.1	30	19.3	2.3	126	24.3%	-2.60 [-3.45, -1.75]
**Total (95% CI)**			**248**			**367**	**100.0%**	**-1.92 [-3.85, 0.02]**

Heterogeneity: $Tau^2 = 3.82$; $Chi^2 = 17.62$, $df = 6$ ($P = 0.007$); $I^2 = 66\%$
Test for overall effect: $Z = 1.94$ ($P = 0.05$)

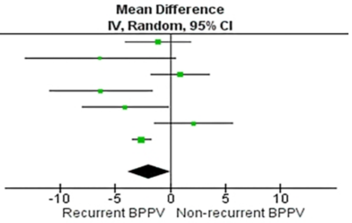

Figure 3. Relationship between serum 25-hydroxyvitamin D and Benign Paroxysmal Positional Vertigo recurrence.

258x59mm (300 x 300 DPI)

Appendix

Appendix Table 1. Systematic review searches performed and results.

Search	*Search terms*	*Database*	*Results*	*Filters*
**1**	(Vitamin D* OR 25-hydroxyvitamin D OR ergocalciferol OR cholecalciferol) AND BPPV	PUBMED	86	Years 2000 - 2021, English Language
**2**	(Vitamin D* OR 25-hydroxyvitamin D OR ergocalciferol OR cholecalciferol) AND "Benign paroxysmal positional vertigo"	PUBMED	86	Years 2000 - 2021, English Language
**3**	(Vitamin D* OR 25-hydroxyvitamin D OR ergocalciferol OR cholecalciferol) AND vertigo	PUBMED	102	Years 2000 - 2021, English Language
**4**	(Vitamin D* OR 25-hydroxyvitamin D OR ergocalciferol OR cholecalciferol) AND BPPV	SCOPUS	85	Year >1999, English Language
**5**	(Vitamin D* OR 25-hydroxyvitamin D OR ergocalciferol OR cholecalciferol) AND "Benign paroxysmal positional vertigo"	SCOPUS	104	Year >1999, English Language
**6**	(Vitamin D* OR 25-hydroxyvitamin D OR ergocalciferol OR cholecalciferol) AND "vertigo"	SCOPUS	300	Year >1999, English Language
**7**	(ALL=(Vitamin D OR 25-hydroxyvitamin D OR ergocalciferol OR cholecalciferol)) AND ALL=(BPPV)	Web of Science	101	Timespan: 2000-01-01 to 2021-08-09, English Language
**8**	(ALL=(Vitamin D OR 25-hydroxyvitamin D OR ergocalciferol OR cholecalciferol)) AND ALL=(Benign Paroxysmal Positional Vertigo)	Web of Science	108	Timespan: 2000-01-01 to 2021-08-09, English Language
**9**	(ALL=(Vitamin D OR 25-hydroxyvitamin D OR ergocalciferol OR cholecalciferol)) AND ALL=(Vertigo)	Web of Science	141	Timespan: 2000-01-01 to 2021-08-09, English Language

For peer review only

Appendix Table 2. Data extracted from included studies for the Meta-analysis exploring the relationship between Vitamin D deficiency and BPPV occurrence.

Study	Participants with BPPV			Control participants (without BPPV)		
	Number of Participants	Vitamin D (Mean: ng/mL)	Vitamin D (SD: ng/mL)	Number of Participants	Vitamin D (Mean: ng/mL)	Vitamin D (SD: ng/mL)
Bi et al. 2021	27	14.64	6.94	25	19.56	6.55
Califano et al. 2019	127	21.08	11	100	25.6	10.5
Çelik et al. 2021	190	15.64	8.4	149	16.29	6.7
Ceylan and Kanmaz 2020	97	15.87	8.61	100	15.82	10.23
Cheng et al. 2021	320	23.2	4.09	320	25.8	3.43
Çıkrıkçı Işık et al. 2017	64	9.51	5.49	63	11.02	9.62
Goldschagg et al. 2021	158	23.4	9.4	301	21.5	10.6
Han et al. 2018	85	19.1	5.2	80	22.5	5.8
Inan et al. 2021	52	15.3	9.8	52	20.2	14.3
Karataş et al. 2017	78	23	14.4	78	17	12.3
Sarsitthithim et al. 2021	69	21.5	5.3	68	26.3	6.8
Sen, Padiyar and Arora 2018	100	20.3	12.2	100	18	10.1
Song et al. 2020	380	14.24	6.6	3125	15.45	7.7
Talaat et al. 2015	80	14.19	9.3	100	19.53	8.45
Thomas et al. 2021	49	21.3	0.57	49	17.6	0.06
Wang et al. 2020	103	17.15	2.03	80	23.85	3.13
Wu et al. 2018	60	20.99	6.76	92	23.17	6.49
Yang et al. 2017	130	18.21	10.3	130	20	8.1
Zhang et al. 2022	156	18.8	2.5	50	24	2.2

For peer review only - http://bmjopen.bmj.com/site/about/guidelines.xhtml

Appendix Table 3. Data extracted from included studies for the Meta-analysis exploring the relationship between Vitamin D deficiency and BPPV recurrence.

	Participants with BPPV Recurrence			Participants without BPPV Recurrence		
**Study**	**Number of Participants**	**Vitamin D (Mean: ng/mL)**	**Vitamin D (SD: ng/mL)**	**Number of Participants**	**Vitamin D (Mean: ng/mL)**	**Vitamin D (SD: ng/mL)**
Çıkrıkçı Işık et al. 2017	16	8.81	4.9	41	9.91	5.81
Melis et al. 2020	43	19.53	15.33	30	25.85	14.1
Sarsitthithim et al. 2021	41	21.9	4.9	28	21	5.9
Shin *et al.* 2023	19	12.9	8	31	19.2	8.2
Talaat et al. 2015	36	11.93	7.57	44	16.04	10.26
Yang et al. 2017	63	19.3	11.1	67	17.2	9.4
Zhang *et al.* 2022	30	16.7	2.1	126	19.3	2.3

For peer review only

Study	Selection of Participants	Confounding Variables	Intervention measurement	Blinding of outcome assessment	Incomplete outcome data	Selective outcome reporting
Bi *et al.* 2021	-	?	-	+	-	-
Califano *et al.* 2019	?	+	-	+	-	-
Carneiro de Sousa *et al.* 2019	-	+	-	+	-	-
Çelik *et al*. 2021	-	-	-	+	-	-
Ceylan 2020	-	+	-	+	-	-
Cheng *et al*. 2021	-	?	-	+	-	-
Çıkrıkçı Işık *et al.* 2017	-	-	-	+	-	-
Ding *et al.* 2019	-	-	-	+	-	-
Elmoursey and Abbas 2021	-	+	-	+	-	-
Goldschlagg *et al.* 2021	-	?	-	+	-	-
Gu, Dong and Gu 2018	?	-	-	-	-	-
Han *et al.* 2018	-	?	-	+	-	-
Inan *et al.* 2021	?	-	-	+	-	-
Karatas *et al.* 2016	?	-	-	+	-	-
Parham *et al.* 2013	?	-	-	-	-	-
Pecci *et al.* 2022	+	-	-	+	-	-
Sarsitthithim *et al.* 2021	?	?	-	+	-	-
Sen, Padiyar and Arora 2018	?	?	-	+	-	-
Song *et al*. 2020	-	-	-	+	-	-
Talaat *et al*. 2015	-	-	-	+	-	-
Thomas *et al*. 2021	-	?	-	+	-	-
Wang *et al*. 2020	?	-	-	+	-	-
Wu *et al*. 2018	-	-	-	+	-	-
Wu *et al*. 2022	-	-	-	+	-	-
Yang *et al*. 2017	?	-	-	+	-	-
Zhang *et al*. 2022	-	-	-	+	-	-

Appendix Figure 1. Assessment of bias for case-control studies according to RoBANs.
Green '-' symbols= low risk of bias, red '+' symbols= high risk of bias and purple '?' symbols = unclear risk of bias.

	Representativeness of exposed cohort	Selection of non-exposed cohort	Ascertainment of exposure	Outcome of interest not present at start	Comparability of cohorts/confounders	Assessment of outcome	Follow-up long enough?	Adequacy of follow-up of cohorts	Study Quality
Han *et al.* 2020	*	*	*	-	**	*	*	n/a	Good
Han *et al*. 2021	-	*	*	-	**	*	*	n/a	Fair
Kahraman *et al.* 2016	*	*	*	-	*	*	*	*	Good
Lee *et al.* 2017	-	*	*	-	**	*	*	n/a	Fair
Maslovara *et al.* 2018	*	*	*	*	**	*	*	*	Good
Melis *et al.* 2020	*	*	*	*	**	*	-	*	Good
Nakada *et al*. 2019	*	*	*	-	-	*	*	n/a	Poor
Shin *et al*. 2023	*	*	*	-	*	*	*	*	Good
Shu *et al*. 2019	*	*	*	-	**	*	*	n/a	Good

Appendix Figure 2. Assessment of bias of cohort studies using the Newcastle-Ottawa Quality Assessment form for cohort studies.
'*' symbols indicate low risk of bias for a given criteria, '-' symbols indicate the study does not meet criteria for low risk of bias and 'n/a' indicates that the criterion does not apply to a given study (for example retrospective studies do not have a follow-up period by design).

For peer review only - http://bmjopen.bmj.com/site/about/guidelines.xhtml

PRISMA 2020 Checklist

Section and Topic	Item #	Checklist item	Location where item is reported
**TITLE**			
Title	1	Identify the report as a systematic review.	Title page (p1)
**ABSTRACT**			
Abstract	2	See the PRISMA 2020 for Abstracts checklist.	Abstract (p2)
**INTRODUCTION**			
Rationale	3	Describe the rationale for the review in the context of existing knowledge.	Introduction (p3)
Objectives	4	Provide an explicit statement of the objective(s) or question(s) the review addresses.	Introduction (p3)
**METHODS**			
Eligibility criteria	5	Specify the inclusion and exclusion criteria for the review and how studies were grouped for the syntheses.	Methods: Inclusion and Exculsion criteria (p4)
Information sources	6	Specify all databases, registers, websites, organisations, reference lists and other sources searched or consulted to identify studies. Specify the date when each source was last searched or consulted.	Methods: Data sources and search strategy (p4)
Search strategy	7	Present the full search strategies for all databases, registers and websites, including any filters and limits used.	Methods: Data sources and search strategy (p4)
Selection process	8	Specify the methods used to decide whether a study met the inclusion criteria of the review, including how many reviewers screened each record and each report retrieved, whether they worked independently, and if applicable, details of automation tools used in the process.	Methods: Inclusion and Exculsion criteria (p4)
Data collection process	9	Specify the methods used to collect data from reports, including how many reviewers collected data from each report, whether they worked independently, any processes for obtaining or confirming data from study investigators, and if applicable, details of automation tools used in the process.	Methods: Data Extraction (p5)
Data items	10a	List and define all outcomes for which data were sought. Specify whether all results that were compatible with each outcome domain in each study were sought (e.g. for all measures, time points, analyses), and if not, the methods used to decide which results to collect.	Methods: Data Extraction (p5)
	10b	List and define all other variables for which data were sought (e.g. participant and intervention characteristics, funding sources). Describe any assumptions made about any missing or unclear information.	Methods: Data Extraction (p5)
Study risk of bias assessment	11	Specify the methods used to assess risk of bias in the included studies, including details of the tool(s) used, how many reviewers assessed each study and whether they worked independently, and if applicable, details of automation tools used in the process.	Methods: Assessment of bias (p5)
Effect measures	12	Specify for each outcome the effect measure(s) (e.g. risk ratio, mean difference) used in the synthesis or presentation of results.	Methods: Synthesis methods (p5)
Synthesis methods	13a	Describe the processes used to decide which studies were eligible for each synthesis (e.g. tabulating the study intervention characteristics and comparing against the planned groups for each synthesis (item #5)).	Methods: Synthesis methods (p5)
	13b	Describe any methods required to prepare the data for presentation or synthesis, such as handling of missing summary statistics, or data conversions.	Methods: Synthesis methods (p5)
	13c	Describe any methods used to tabulate or visually display results of individual studies and syntheses.	Methods: Synthesis methods (p5)
	13d	Describe any methods used to synthesize results and provide a rationale for the choice(s). If meta-analysis was performed, describe the model(s), method(s) to identify the presence and extent of statistical heterogeneity, and software package(s) used.	Methods: Synthesis methods (p5)
	13e	Describe any methods used to explore possible causes of heterogeneity among study results (e.g. subgroup analysis, meta-	Not applicable

For peer review only - http://bmjopen.bmj.com/site/about/guidelines.xhtml

PRISMA 2020 Checklist

Section and Topic	Item #	Checklist item	Location where item is reported
		regression).	
	13f	Describe any sensitivity analyses conducted to assess robustness of the synthesized results.	Not applicable
Reporting bias assessment	14	Describe any methods used to assess risk of bias due to missing results in a synthesis (arising from reporting biases).	Not applicable
Certainty assessment	15	Describe any methods used to assess certainty (or confidence) in the body of evidence for an outcome.	Not applicable
**RESULTS**			
Study selection	16a	Describe the results of the search and selection process, from the number of records identified in the search to the number of studies included in the review, ideally using a flow diagram.	Results: Study selection (p5-6); Figure 1. Flow diagram detailing results of searches and study selection process; Appendix 1. Systematic review searches performed and results
	16b	Cite studies that might appear to meet the inclusion criteria, but which were excluded, and explain why they were excluded.	Results: Study selection (p5-6); Figure 1. Flow diagram detailing results of searches and study selection process; Appendix 1. Systematic review searches performed and results
Study characteristics	17	Cite each included study and present its characteristics.	Results: Description of included studies (p6-7)
Risk of bias in studies	18	Present assessments of risk of bias for each included study.	Results: Quality assessment (p6); Appendix 2 Assessment of bias for case-control studies according to RoBANs; Appendix 3 Assessment of bias of cohort studies using the Newcastle-Ottawa Quality Assessment form for cohort studies
Results of individual studies	19	For all outcomes, present, for each study: (a) summary statistics for each group (where appropriate) and (b) an effect estimate and its precision (e.g. confidence/credible interval), ideally using structured tables or plots.	Table 1. Characteristics of included studies
Results of syntheses	20a	For each synthesis, briefly summarise the characteristics and risk of bias among contributing studies.	Table 1. Characteristics of included studies<br>Figure 5. Relationship between serum 25-hydroxyvitamin D and Benign Paroxysmal Positional Vertigo recurrence.
	20b	Present results of all statistical syntheses conducted. If meta-analysis was done, present for each the summary estimate and its precision (e.g. confidence/credible interval) and measures of statistical heterogeneity. If comparing groups, describe the	Results: Meta analysis (p7)

For peer review only

For peer review only - http://bmjopen.bmj.com/site/about/guidelines.xhtml

PRISMA 2020 Checklist

Section and Topic	Item #	Checklist item	Location where item is reported
		direction of the effect.	Figure 5. Relationship between serum 25-hydroxyvitamin D and Benign Paroxysmal Positional Vertigo recurrence.
	20c	Present results of all investigations of possible causes of heterogeneity among study results.	Not applicable
	20d	Present results of all sensitivity analyses conducted to assess the robustness of the synthesized results.	Not applicable
Reporting biases	21	Present assessments of risk of bias due to missing results (arising from reporting biases) for each synthesis assessed.	Not applicable
Certainty of evidence	22	Present assessments of certainty (or confidence) in the body of evidence for each outcome assessed.	Not applicable
**DISCUSSION**			
Discussion	23a	Provide a general interpretation of the results in the context of other evidence.	Discussion (p9-10)
	23b	Discuss any limitations of the evidence included in the review.	Strengths and limitations of the study (p2); Discussion (p9-10)
	23c	Discuss any limitations of the review processes used.	Strengths and limitations (p.2)
	23d	Discuss implications of the results for practice, policy, and future research.	Discussion (p9-10)
**OTHER INFORMATION**			Prospero registration number (p.2)
Registration and protocol	24a	Provide registration information for the review, including register name and registration number, or state that the review was not registered.	Abstract: Trial registration (p2)
	24b	Indicate where the review protocol can be accessed, or state that a protocol was not prepared.	Prospero registration number (p.2)
	24c	Describe and explain any amendments to information provided at registration or in the protocol.	Prospero registration number (p.2)
Support	25	Describe sources of financial or non-financial support for the review, and the role of the funders or sponsors in the review.	Abstract: Funding sources (p2)
Competing interests	26	Declare any competing interests of review authors.	Competing interests statement (p2)
Availability of data, code and other materials	27	Report which of the following are publicly available and where they can be found: template data collection forms; data extracted from included studies; data used for all analyses; analytic code; any other materials used in the review.	Methods: Synthesis methods (p5) and Appendix tables 2 and 3

From: Page MJ, McKenzie JE, Bossuyt PM, Boutron I, Hoffmann TC, Mulrow CD, et al. The PRISMA 2020 statement: an updated guideline for reporting systematic reviews. BMJ 2021;372:n71. doi: 10.1136/bmj.n71

For more information, visit: http://www.prisma-statement.org/

For peer review only

PRISMA 2020 for Abstracts Checklist

Section and Topic	Item #	Checklist item	Reported (Yes/No)
**TITLE**			
Title	1	Identify the report as a systematic review.	YES
**BACKGROUND**			
Objectives	2	Provide an explicit statement of the main objective(s) or question(s) the review addresses.	YES
**METHODS**			
Eligibility criteria	3	Specify the inclusion and exclusion criteria for the review.	YES
Information sources	4	Specify the information sources (e.g. databases, registers) used to identify studies and the date when each was last searched.	YES
Risk of bias	5	Specify the methods used to assess risk of bias in the included studies.	YES
Synthesis of results	6	Specify the methods used to present and synthesise results.	YES
**RESULTS**			
Included studies	7	Give the total number of included studies and participants and summarise relevant characteristics of studies.	YES
Synthesis of results	8	Present results for main outcomes, preferably indicating the number of included studies and participants for each. If meta-analysis was done, report the summary estimate and confidence/credible interval. If comparing groups, indicate the direction of the effect (i.e. which group is favoured).	YES
**DISCUSSION**			
Limitations of evidence	9	Provide a brief summary of the limitations of the evidence included in the review (e.g. study risk of bias, inconsistency and imprecision).	YES
Interpretation	10	Provide a general interpretation of the results and important implications.	YES
**OTHER**			
Funding	11	Specify the primary source of funding for the review.	YES
Registration	12	Provide the register name and registration number.	YES

From: Page MJ, McKenzie JE, Bossuyt PM, Boutron I, Hoffmann TC, Mulrow CD, et al. The PRISMA 2020 statement: an updated guideline for reporting systematic reviews. BMJ 2021;372:n71. doi: 10.1136/bmj.n71

For more information, visit: http://www.prisma-statement.org/

For peer review only

Systematic review

*Fields that have an **asterisk (*)** next to them means that they **must be answered. Word limits** are provided for each section. You will be unable to submit the form if the word limits are exceeded for any section. Registrant means the person filling out the form.*

1. * Review title.

Give the title of the review in English

Association Between Vitamin D deficiency and Benign Paroxysmal Positional Vertigo: A Systematic Review and Meta-Analysis

2. Original language title.

For reviews in languages other than English, give the title in the original language. This will be displayed with the English language title.

Association Between Vitamin D deficiency and Benign Paroxysmal Positional Vertigo: A Systematic Review and Meta-Analysis

3. * Anticipated or actual start date.

Give the date the systematic review started or is expected to start.

09/08/2021

4. * Anticipated completion date.

Give the date by which the review is expected to be completed.

15/02/2022

5. * Stage of review at time of this submission.

Tick the boxes to show which review tasks have been started and which have been completed.
Update this field each time any amendments are made to a published record.

Reviews that have started data extraction (at the time of initial submission) are not eligible for inclusion in PROSPERO.
If there is later evidence that incorrect status and/or completion date has been supplied, the published PROSPERO record will be marked as retracted.

This field uses answers to initial screening questions. It cannot be edited until after registration.

The review has not yet started: No

Review stage	Started	Completed
Preliminary searches	Yes	No
Piloting of the study selection process	Yes	No
Formal screening of search results against eligibility criteria	No	No
Data extraction	No	No
Risk of bias (quality) assessment	No	No
Data analysis	No	No

Provide any other relevant information about the stage of the review here.

Previous work completed in MSc dissertation including literature up to January 2018. This review to extend on that work.
Previous work completed in MSc dissertation including literature up to January 2018. This review to extend on that work.

6. * Named contact.

The named contact is the guarantor for the accuracy of the information in the register record. This may be any member of the review team.

Heather Wood

Email salutation (e.g. "Dr Smith" or "Joanne") for correspondence:
Ms Wood

7. * Named contact email.

Give the electronic email address of the named contact.

hmw998@student.bham.ac.uk

8. Named contact address

PLEASE NOTE this information will be published in the PROSPERO record so please do not enter private information, i.e. personal home address

Give the full institutional/organisational postal address for the named contact.

School of Medicine and Dentistry, University of Birmingham, Edgbaston, Birmingham B15 2TT

9. Named contact phone number.

Give the telephone number for the named contact, including international dialling code.

07516621378

10. * Organisational affiliation of the review.

Full title of the organisational affiliations for this review and website address if available. This field may be completed as 'None' if the review is not affiliated to any organisation.

University of Birmingham

Organisation web address:

11. * Review team members and their organisational affiliations.

Give the personal details and the organisational affiliations of each member of the review team. Affiliation refers to groups or organisations to which review team members belong.
NOTE: email and country now MUST be entered for each person, unless you are amending a published record.

Ms Heather Wood. University of Birmingham, Birmingham, UK
Dr Ghada BinKhamis. University of Manchester, Manchester, UK. King Fahad Medical City, Riyadh, Saudi Arabia

12. * Funding sources/sponsors.

Details of the individuals, organizations, groups, companies or other legal entities who have funded or sponsored the review.

None

Grant number(s)
State the funder, grant or award number and the date of award

13. * Conflicts of interest.

List actual or perceived conflicts of interest (financial or academic).

None

14. Collaborators.

Give the name and affiliation of any individuals or organisations who are working on the review but who are not listed as review team members. **NOTE: email and country must be completed for each person, unless you are amending a published record.**

15. * Review question.

State the review question(s) clearly and precisely. It may be appropriate to break very broad questions down into a series of related more specific questions. Questions may be framed or refined using PI(E)COS or similar where relevant.

How do serum vitamin D levels affect the incidence and recurrence rates of benign paroxysmal positional vertigo (BPPV) in adult patients in the Northern Hemisphere?

16. * Searches.

State the sources that will be searched (e.g. Medline). Give the search dates, and any restrictions (e.g. language or publication date). Do NOT enter the full search strategy (it may be provided as a link or attachment below.)

We will search the following electronic bibliographic databases: PubMed, NICE, Scopus, Web of Science
using the following search terms: "Vitamin D* OR 25-hydroxyvitamin D OR ergocalciferol OR cholecalciferol AND benign paroxysmal positional vertigo", "Vitamin D* OR 25-hydroxyvitamin D OR ergocalciferol OR cholecalciferol AND BPPV" "Vitamin D* OR 25-hydroxyvitamin D OR ergocalciferol OR cholecalciferol AND vertigo"

Research papers published between 2000 and 2021 will be reviewed and language is restricted with English.

- Searches will be re-run prior to the final analysis to identify any further studies.
- Unpublished studies will not be sought

17. URL to search strategy.

Upload a file with your search strategy, or an example of a search strategy for a specific database, (including the keywords) in pdf or word format. In doing so you are consenting to the file being made publicly accessible.

Or provide a URL or link to the strategy. Do NOT provide links to your search **results**.

Do not make this file publicly available until the review is complete

18. * Condition or domain being studied.

Give a short description of the disease, condition or healthcare domain being studied in your systematic review.

Benign Paroxysmal positional vertigo (BPPV) and serum vitamin D levels

19. * Participants/population.

Specify the participants or populations being studied in the review. The preferred format includes details of both inclusion and exclusion criteria.

Inclusion criteria: Participants over the age of 18 with at least one episode of diagnosed benign paroxysmal positional vertigo. Geographical location of study participants in Northern hemisphere. Participants not taking vitamin D supplements, no other comorbid diagnoses.

Exclusion criteria: children below the age of 18 with at least one episode of diagnosed benign paroxysmal positional vertigo. Geographical location of study participants not in Northern hemisphere. Participants taking vitamin D supplements, participants with other comorbid diagnoses.

20. * Intervention(s), exposure(s).

Give full and clear descriptions or definitions of the interventions or the exposures to be reviewed. The preferred format includes details of both inclusion and exclusion criteria.

Serum 25-hydroxyvitamin D levels

21. * Comparator(s)/control.

Where relevant, give details of the alternatives against which the intervention/exposure will be compared (e.g. another intervention or a non-exposed control group). The preferred format includes details of both inclusion and exclusion criteria.

Normal controls over the age of 18 with no past medical history of benign paroxysmal positional vertigo

22. * Types of study to be included.

Give details of the study designs (e.g. RCT) that are eligible for inclusion in the review. The preferred format includes both inclusion and exclusion criteria. If there are no restrictions on the types of study, this should be stated.

Randomised controlled trial, cohort study or case-controlled study

23. Context.

Give summary details of the setting or other relevant characteristics, which help define the inclusion or exclusion criteria.

Studies in hospital ENT outpatient departments. Research including participants in southern hemisphere countries will be excluded

24. * Main outcome(s).

Give the pre-specified main (most important) outcomes of the review, including details of how the outcome is defined and measured and when these measurement are made, if these are part of the review inclusion criteria.

Incidence of benign paroxysmal positional vertigo. Recurrence of benign paroxysmal positional vertigo

Measures of effect

Difference in average serum vitamin D levels between patients with new or recurrent benign paroxysmal positional vertigo compared to controls.

25. * Additional outcome(s).

List the pre-specified additional outcomes of the review, with a similar level of detail to that required for main outcomes. Where there are no additional outcomes please state ‘None’ or ‘Not applicable’ as appropriate to the review

Not applicable

Measures of effect

26. * Data extraction (selection and coding).

Describe how studies will be selected for inclusion. State what data will be extracted or obtained. State how this will be done and recorded.

Study selection: one author will select and this selection will be reviewed by two authors.
The final decision will be recorded in an excel spreadsheet.
Any disagreements will be resolved by discussion between the authors.

27. * Risk of bias (quality) assessment.

State which characteristics of the studies will be assessed and/or any formal risk of bias/quality assessment tools that will be used.

Any randomised controlled trials will be assessed using the Cochrane Collaboration’s tool for assessing risk of bias in randomised trials.

Case-control studies will be assessed using the Risk of Bias Assessment Tool for Nonrandomized Studies (RoBANS) tool.

Cohort studies will be assessed according to the Newcastle Ottawa Scale (NOS).

Records of risk of bias will be recorded in Microsoft Word and Microsoft Excel files.

28. * Strategy for data synthesis.

Describe the methods you plan to use to synthesise data. This **must not be generic text** but should be **specific to your review** and describe how the proposed approach will be applied to your data.
If meta-analysis is planned, describe the models to be used, methods to explore statistical heterogeneity, and software package to be used.

Extracted data will include:
Study design; country; author and published year; mean age of participants; range of participant age; sample size; disease diagnosis; percentage male participants and serum vitamin D level for cases and controls.

Standardised mean difference (SMD) with a 95% confidence interval will be used to calculate the level of vitamin D in case and control groups. Plasma vitamin D levels of greater than 20 ng/ml will be considered to be sufficient. The Q statistic will be used to examine heterogeneity between studies, with P<0.10 regarded as significant. The random-effect model will be employed for the meta-analyses if P<0.10, otherwise the fixed-effect model will be used instead.

Statistical analysis will be completed using RevMan software version 5.4.1

29. * Analysis of subgroups or subsets.

State any planned investigation of ‘subgroups’. Be clear and specific about which type of study or participant will be included in each group or covariate investigated. State the planned analytic approach.

Vitamin D status will be categorised into "sufficient" if serum levels are >20ng/l; or "insufficient" if serum levels <20ng/L

There may be differences between patients experiencing recurrent BPPV and those experiencing single episodes. Recurrent BPPV will be classified as symptom recurrence within 3 years of initial diagnosis.

30. * Type and method of review.

Select the type of review, review method and health area from the lists below.

Type of review

Cost effectiveness	No
Diagnostic	No
Epidemiologic	No
Individual patient data (IPD) meta-analysis	No
Intervention	No
Living systematic review	No
Meta-analysis	Yes
Methodology	No
Narrative synthesis	No
Network meta-analysis	No
Pre-clinical	No
Prevention	No
Prognostic	No
Prospective meta-analysis (PMA)	No

Review of reviews	No
Service delivery	No
Synthesis of qualitative studies	No
Systematic review	Yes
Other	No

Health area of the review

Alcohol/substance misuse/abuse	No
Blood and immune system	No
Cancer	No
Cardiovascular	No
Care of the elderly	No
Child health	No
Complementary therapies	No
COVID-19	No
Crime and justice	No
Dental	No
Digestive system	No
Ear, nose and throat	Yes
Education	No
Endocrine and metabolic disorders	No
Eye disorders	No
General interest	No
Genetics	No
Health inequalities/health equity	No
Infections and infestations	No
International development	No
Mental health and behavioural conditions	No
Musculoskeletal	No
Neurological	No

Nursing	No
Obstetrics and gynaecology	No
Oral health	No
Palliative care	No
Perioperative care	No
Physiotherapy	No
Pregnancy and childbirth	No
Public health (including social determinants of health)	No
Rehabilitation	No
Respiratory disorders	No
Service delivery	No
Skin disorders	No
Social care	No
Surgery	No
Tropical Medicine	No
Urological	No
Wounds, injuries and accidents	No
Violence and abuse	No

31. Language.

Select each language individually to add it to the list below, use the bin icon to remove any added in error.

English

There is not an English language summary

32. * Country.

Select the country in which the review is being carried out. For multi-national collaborations select all the countries involved.

England

33. Other registration details.

Name any other organisation where the systematic review title or protocol is registered (e.g. Campbell, or The Joanna Briggs Institute) together with any unique identification number assigned by them.
If extracted data will be stored and made available through a repository such as the Systematic Review Data Repository (SRDR), details and a link should be included here. If none, leave blank.

34. Reference and/or URL for published protocol.

If the protocol for this review is published provide details (authors, title and journal details, preferably in Vancouver format)

No I do not make this file publicly available until the review is complete

35. Dissemination plans.

Do you intend to publish the review on completion?

Yes

Intention to publish completed systematic review and meta-analysis in appropriate peer-reviewed journal

36. Keywords.

Give words or phrases that best describe the review. Separate keywords with a semicolon or new line. Keywords help PROSPERO users find your review (keywords do not appear in the public record but are included in searches). Be as specific and precise as possible. Avoid acronyms and abbreviations unless these are in wide use.

Vitamin D, 25-hydroxyvitamin D, 25(OH)D, meta-analysis, benign paroxysmal positional vertigo, BPPV

37. Details of any existing review of the same topic by the same authors.

If you are registering an update of an existing review give details of the earlier versions and include a full bibliographic reference, if available.

38. * Current review status.

Update review status when the review is completed and when it is published.
New registrations must be ongoing so this field is not editable for initial submission.

Review_Ongoing

39. Any additional information.

Provide any other information relevant to the registration of this review.

40. Details of final report/publication(s) or preprints if available.

Leave empty until publication details are available OR you have a link to a preprint (NOTE: this field is not editable for initial submission).
List authors, title and journal details preferably in Vancouver format.

BMJ Open

Association between vitamin D deficiency and Benign Paroxysmal Positional Vertigo (BPPV) incidence and recurrence: A systematic review and meta-analysis

Journal:	*BMJ Open*
Manuscript ID	bmjopen-2023-077986.R1
Article Type:	Original research
Date Submitted by the Author:	31-Dec-2023
Complete List of Authors:	Wood, Heather; University of Birmingham, College of Medical and Dental Sciences<br>Kluk, Karolina; The University of Manchester, Manchester Centre for Audiology and Deafness<br>Binkhamis, Ghada; The University of Manchester, Manchester Centre for Audiology and Deafness (ManCAD) ; King Fahad Medical City, Communication and Swallowing Disorders
**Primary Subject Heading**:	Ear, nose and throat/otolaryngology
Secondary Subject Heading:	General practice / Family practice
Keywords:	Audiology < OTOLARYNGOLOGY, Adult otolaryngology < OTOLARYNGOLOGY, Systematic Review, Meta-Analysis

SCHOLARONE™
Manuscripts

I, the Submitting Author has the right to grant and does grant on behalf of all authors of the Work (as defined in the below author licence), an exclusive licence and/or a non-exclusive licence for contributions from authors who are: i) UK Crown employees; ii) where BMJ has agreed a CC-BY licence shall apply, and/or iii) in accordance with the terms applicable for US Federal Government officers or employees acting as part of their official duties; on a worldwide, perpetual, irrevocable, royalty-free basis to BMJ Publishing Group Ltd ("BMJ") its licensees and where the relevant Journal is co-owned by BMJ to the co-owners of the Journal, to publish the Work in this journal and any other BMJ products and to exploit all rights, as set out in our licence.

The Submitting Author accepts and understands that any supply made under these terms is made by BMJ to the Submitting Author unless you are acting as an employee on behalf of your employer or a postgraduate student of an affiliated institution which is paying any applicable article publishing charge ("APC") for Open Access articles. Where the Submitting Author wishes to make the Work available on an Open Access basis (and intends to pay the relevant APC), the terms of reuse of such Open Access shall be governed by a Creative Commons licence – details of these licences and which Creative Commons licence will apply to this Work are set out in our licence referred to above.

Other than as permitted in any relevant BMJ Author's Self Archiving Policies, I confirm this Work has not been accepted for publication elsewhere, is not being considered for publication elsewhere and does not duplicate material already published. I confirm all authors consent to publication of this Work and authorise the granting of this licence.

For peer review only

Association between vitamin D deficiency and Benign Paroxysmal Positional Vertigo (BPPV) incidence and recurrence: A systematic review and meta-analysis

Heather Wood[1], Karolina Kluk[2], Ghada BinKhamis[2,3]

[1]*College of Medical and Dental Sciences, University of Birmingham, United Kingdom;* [2]*Manchester Centre for Audiology and Deafness, Manchester Academic Health Science Centre, University of Manchester, Manchester, UK;* [3]*Department of Communication and Swallowing Disorders, King Fahad Medical City, Riyadh, Saudi Arabia*

Address for correspondence:

Ghada BinKhamis: ghada.binkhamis@manchester.ac.uk / gbinkhamis@gmail.com

Karolina Kluk: Karolina.Kluk@manchester.ac.uk

Heather Wood: hmw998@student.bham.ac.uk

Manchester Centre for Audiology and Deafness (ManCAD), School of Health Sciences, Faculty of Biology Medicine and Health, Room B2.15, Ellen Wilkinson Building, University of Manchester, Oxford Road, Manchester, M13 9PL, United Kingdom.

ORCiD:

- Ghada BinKhamis: 0000-0002-7696-1073
- Karolina Kluk: 0000-0003-3638-2787

Word Count: 3951

For peer review only

For peer review only - http://bmjopen.bmj.com/site/about/guidelines.xhtml

Abstract

OBJECTIVES: To determine the relationship between serum vitamin D level and the risk of developing benign paroxysmal positional vertigo (BPPV) incidence and recurrence in countries in the Northern hemisphere.

DESIGN: Systematic review and meta-analysis.

DATA SOURCES: PUBMED, SCOPUS and Web of Science databases were searched for studies published between January 2000 and February 2023.

ELIGIBILITY CRITERIA FOR SELECTING STUDIES: Participants located in the Northern hemisphere aged 18 or over with at least one episode of BPPV, serum 25-hydroxyvitamin D levels measured and reported, no comorbidities or history of vitamin D supplementation.

DATA EXTRACTION AND SYNTHESIS: Were performed by a single reviewer and checked by a second reviewer. Inclusion and exclusion criteria and risk of bias were assessed by 2 independent reviewers using the Newcastle Ottawa Tool for Cohort studies and RoBANS checklist for case-control studies. Meta-analysis was conducted using random effects models. Standard mean difference with a 95% confidence interval was used to measure the relationship between vitamin D level and BPPV.

RESULTS: The 35 articles identified by the literature search reported data of 9843 individuals. 19 studies (7387 individuals) were included in the BPPV incidence meta-analysis whilst seven studies (622 individuals) were included in the BPPV recurrence meta-analysis. Lower serum vitamin D levels were found in BPPV incidence compared to controls, but the relationship between vitamin D levels in recurrent BPPV compared to non-recurrent disease remained uncertain.

CONCLUSION: Results of this systematic review and meta-analysis demonstrated a negative correlation between serum vitamin D and BPPV incidence, whilst any relationship between serum vitamin D and BPPV recurrence remained uncertain. Risk of bias analysis revealed evidence of variable quality. There was insufficient data available to evaluate seasonal relationships between serum vitamin D and BPPV. Given the potential for this as a confounding factor, future research should aim to investigate this further.

PROSPERO REGISTRATION: CRD42021271840

For peer review only

For peer review only - http://bmjopen.bmj.com/site/about/guidelines.xhtml

Strengths and limitations of the study

- Strengths:
    - This is a comprehensive systematic review of vitamin D and Benign Paroxysmal Positional Vertigo (BPPV) covering literature published between January 2000 and February 2023
    - The meta-analyses include a large number of individuals (7510)
- Limitations:
    - It was not possible to account for the impact of seasonality on both serum vitamin D levels and incidence and recurrence rates of BPPV as the majority of studies did not report the dates or months when patients were seen.
    - Data were not available relating to factors potentially related to both BPPV and vitamin D such as subject occupation, comorbidities and symptom severity.
    - High-quality research is currently limited, and a few of the identified studies in this systematic review were from journals with unclear peer-review processes or non-impact journals.

Keywords:

BPPV, Benign Paroxysmal Positional Vertigo, Vitamin D, Vitamin D Deficiency

For peer review only

Introduction

The impact of vitamin D on audiovestibular health has been a research area of interest in recent years, with low serum levels of vitamin D associated with otological pathologies including benign paroxysmal positional vertigo (BPPV).[1] Due to vitamin D's role in skeletal mineral homeostasis, it has been suggested that audiovestibular pathology may be related to high bone turnover rates in the temporal bone and/or at a systemic level.[2] Another hypothesis suggests that inner ear pathology including BPPV may be secondary to an inflammatory or autoimmune response following viral infection, and that the immunomodulatory functions of vitamin D may be protective against this secondary response.[3]

There are two major bioavailable forms of vitamin D; vitamin D2 (also known as ergocalciferol) and vitamin D3 (cholecalciferol). Ergocalciferol can be ingested via diet or supplementation, whilst vitamin D3 is largely synthesised in human skin following exposure to UVB light (and may also be ingested via dietary supplements or animal-based foods).[4] The bioactive form of vitamin D3 is known to play an important role in calcium homeostasis and bone health; increasing the absorption of dietary calcium and phosphorus which in turn affects the regulation of bone mineral density.[5] Vitamin D3 is hydroxylated in the liver into 25-hydroxyvitamin D (calcifediol), before being processed further by the kidneys, converting it to its bioactive form (calcitriol).[5] Levels of the calcitriol precursor 25-hydroxyvitamin D (calcidiol) can be measured from blood samples and used to indicate the overall abundance of vitamin D within the body.

BPPV is a common vestibular disorder, characterised by positional vertigo and nystagmus elicited by head movements. It is caused by the displacement of small crystals of calcium carbonate known as otoconia into one of the semi-circular canals (most commonly a posterior canal). Displacement of otoconia can occur following head trauma, however most cases are idiopathic and the exact pathophysiology of BPPV currently not known.[6]

The relationship between vitamin D and BPPV is currently a popular topic of research, and a number of systematic reviews have been published in recent years indicating that BPPV incidence is associated with vitamin D deficiency,[7 8] although this relationship was not supported by an earlier review.[9] There is mixed evidence regarding the relationship between vitamin D levels and BPPV recurrence, with some reviews finding lower levels of the vitamin in patients with recurrent BPPV,[9 10] whilst others have observed no significant difference in vitamin D levels and BPPV recurrence.[11] Supplementation in BPPV patients with low vitamin D levels has also been shown to result in prolonged improvement in symptoms and fewer episodes of recurrent BPPV.[12 13]

There is currently uncertainty relating to how seasonal patterns of vitamin D and BPPV incidence are related. In countries within the northern hemisphere, vitamin D levels fluctuate in a seasonal pattern-serum vitamin D levels are highest within the population of these countries during summer months and lowest during winter and spring, when there is a high prevalence of vitamin deficiency.[14 15] BPPV may also exhibit a seasonal pattern, with higher incidence rates seen between autumn and early spring,[16 – 18] although this has not been replicated in every study.[19 20]

This systematic review will examine the evidence concerning the relationship between serum vitamin D and BPPV for patients in northern hemisphere countries, the first time to the authors' knowledge that a review on this topic has been restricted by geographic location.

For peer review only - http://bmjopen.bmj.com/site/about/guidelines.xhtml

Methods

This systematic review was conducted following the methodology of Higgins *et al.*[21] as closely as possible, and was reported following the Preferred Reporting Items for Systematic reviews and Meta-Analyses (PRISMA),[22] and was pre-registered in **PROSPERO (**CRD42021271840, see Supplementary 1) Ethics approval was not necessary for this study as no new data were collected and no patient-identifiable data included.

Patient and public involvement. No patient involved.

Data sources and search strategy

A systematic literature search was performed in PUBMED, SCOPUS and Web of Science databases relating to the association between vitamin D and benign paroxysmal positional vertigo (BPPV). Unpublished studies were not sought. Date was restricted to include studies published in English between 2000 and 2023. Three searches were run through each database to account for differing possible terms for BPPV, making up a total of 12 searches which were completed on the 18th of February 2023. Search criteria and results are described in Supplementary 2, Table 1. Microsoft Excel software (Microsoft Office for Windows, version 2204) was used to store and process search results.

Studies published before the year 2000 and those pertaining to animal research were removed, then any entries that did not contain keywords "BPPV", "Vertigo", "Vitamin D" or "25-hydroxyvitamin D" within their title or abstract were excluded. Entries that were not original research or did not include multiple participants (e.g., case reports, letters and editorials) were also removed, leaving a total of 68 full texts to be manually reviewed. A manual search of the reference list of included full texts and systematic reviews identified by the search strategy was also performed to identify any further relevant literature. The included articles were reviewed independently by two reviewers for inclusion evaluation. Any inconsistencies between the two reviewers were evaluated further by both reviewers until a consensus was reached.

Inclusion and Exclusion criteria

To better account for seasonal differences in serum vitamin D levels, the geographical location of study participants was limited to the Northern hemisphere. All studies that were available with English full texts that investigated the association between serum 25-hydroxyvitamin D levels in adult patients diagnosed with at least one episode of benign paroxysmal positional vertigo (BPPV) were included. Studies were excluded if participants had past medical history of ENT comorbidities, head trauma or were taking vitamin D supplements at the start of the study.

The literature search was undertaken by HW. Study selection was independently undertaken by HW and GB, with inconsistencies resolved through discussion between the reviewers. Studies were deemed eligible for inclusion if they met the following criteria: 1) participants were over the age of 18 with at least one episode of diagnosed BPPV 2) participants resided in countries in the Northern hemisphere; 3) serum 25-hydroxyvitamin D levels were measured and reported; 4) participants had no past medical history of head trauma or ENT comorbidities.

Articles were excluded according to the following criteria: 1) Study did not contain reference to "BPPV", "vertigo" or "vitamin D" in title or abstract; 2) participants were taking vitamin D supplements prior to commencement of the study; 3) The article did not pertain to a research study (reviews, editorials, case studies). During application of inclusion and exclusion criteria, the decision was taken to exclude articles which included only participants with BPPV and pre-existing vitamin D deficiency as

For peer review only - http://bmjopen.bmj.com/site/about/guidelines.xhtml

these would not be able to be utilised in subsequent analysis of the relationship between BPPV disease state and serum vitamin levels.

Assessment of bias

Microsoft Excel and Microsoft Word software (Microsoft Office for Windows, version 2204) was used by the reviewers to record assessment of bias and document comments.

Assessment of bias was performed by two independent reviewers and any inconsistencies resolved by discussion between the reviewers. The case-control studies were assessed using the Risk of Bias Assessment Tool for Nonrandomized Studies (RoBANS)[23] which examines selection of participants, confounding variables, intervention measurement, blinding of outcome assessment, intervention measurement, incomplete outcome data and selective outcome reporting. The cohort studies were assessed according to the Newcastle Ottawa Scale (NOS),[24] as recommended by the Cochrane Collaboration.[21] The NOS examines participant selection, comparability and outcome and assigns studies as being of 'poor', 'fair' or 'good' quality.

Data Extraction

Data extraction was completed by HW and double checked by GB. Extracted data included: study design, author, year published, location of study participants, mean age of participants, range of participant age, sample size, percentage male participants, serum vitamin D levels for participants and the season study was performed (where reported). Standardised mean difference (SMD) with a 95% confidence interval was used to calculate the level of vitamin D in case and control groups. Based on recommendations from the clinical practice guidelines of the Endocrine Society Task Force on Vitamin D, plasma vitamin D levels of less than 20ng/ml were considered to indicate vitamin deficiency.[25] Microsoft Excel software (Microsoft Office for Windows, version 2204) was used to tabulate the extracted data.

Synthesis methods

Studies were included if serum 25-hydroxyvitamin D levels (mean and standard deviation; ng/ml) were reported for cases with BPPV and controls with no medical history of BPPV. Where vitamin D levels were reported for subgroups within a study (for example male and female means reported separately), a population mean and standard deviation for vitamin D levels for both cases and controls was calculated according to the recommendations of the Cochrane Collaboration.[21]

The I^2 statistic was used to examine heterogeneity between studies, with I^2 >40% regarded as representing significant heterogeneity. The random-effect model was employed for the meta-analysis if I^2 >40%, otherwise the fixed-effect model was to be used instead. Statistical analysis was completed and forest plots generated using Review Manager (RevMan) software version 5.4.1. (64 bit; The Cochrane Collaboration, 2020).

<u>Results</u>

Study selection

The initial searches identified 1113 results comprising 350 individual articles (**Figure 1**). 282 of these were excluded; 163 articles were excluded because they did not contain reference to "BPPV" or "vertigo" within their title or abstract, 61 were removed as they were not peer reviewed or based on original research (review articles, conference posters, editorials etc.), 27 did not contain "vitamin D"

For peer review only - http://bmjopen.bmj.com/site/about/guidelines.xhtml

or related terms in their title or abstract, 6 were excluded as they related to animal studies and 25 studies were removed as they were erroneously included in search results despite being published before the year 2000.

The full text of the remaining 68 articles was reviewed for inclusion evaluation; eight articles were excluded as the participants were receiving vitamin D supplementation before commencement of the study; two were excluded as they included patients with past medical history of head trauma or ENT-related comorbidities; five were excluded as serum vitamin D levels were not measured or reported; two were excluded as the patients resided in countries in the Southern hemisphere; seven because they included participants under the age of 18 and a further five were excluded as they described patients with causes of vertigo other than BPPV. Four articles were also excluded as they included only participants with both BPPV and pre-existing vitamin D deficiency, precluding analysis of the relationship between disease state and serum vitamin levels. A manual search of the reference lists of these studies and identified systematic reviews did not yield any additional articles to include in the review. A total of 35 articles underwent full-length review.

Quality assessment

The 26 articles assessed with the RoBANS tool are described in Supplementary 2, Figure 1. All the studies were found to have a low risk of bias in three of the six domains assessed ("intervention measurement", "incomplete outcome data" and "selective outcome reporting" domains). 14 of the studies had an uncertain risk of bias in either or both of the "selection of participant" and "confounding variables" domains. All but two of the studies had a high risk of bias in the domain relating to "blinding of outcome assessment", four had a high risk of bias relating to "confounding variables" and one article had a high risk of bias in the "selection of participants" domain. None of the articles was judged to have a low risk of bias in all six domains.

The nine cohort studies assessed using the Newcastle Ottawa scale (NoS) ranged in quality from 'poor' to 'good' and are described in Supplementary 2, Figure 2. There were significant limitations using the NoS for some of the studies, meaning that one of the eight domains of the tool ("adequacy of follow-up of cohorts") was not applicable for five of the nine studies assessed by this tool. One study was determined to be of 'poor' quality, two 'fair' quality and the remaining six studies were 'good' quality. High risk of bias was most commonly seen in the "outcome of interest not present at start domain" and was present in seven out of nine studies. High risk of bias was seen in two studies in the "representativeness of exposed cohort" domain and in one study for the "follow-up long enough" domain. A single study was found to have a low risk of bias in all eight domains.

Description of included studies

Table 1 describes the characteristics of the included studies investigating the association between serum vitamin D and BPPV for patients within northern hemisphere countries. The 35 studies included 9,843 participants in total: 26 were case-control studies and 9 were cohort studies; there were no randomised controlled trials. Sample size varied from 10 to 3505 individuals, sex distribution from 0 to 100% male and age ranged between 18 to 89 years. Most studies were conducted in countries situated in Asia (twelve in China, six in Turkey, four in the Republic of Korea, two in India, and one each in Japan, Kuwait and Thailand); six studies in Europe (three in Italy and one each in Croatia, Germany and Portugal); one study was conducted in Africa (Egypt) and one in North America (USA).

A total of 12 studies reported that compared with controls, patients with BPPV have lower average serum vitamin D levels.[1 26 – 35] Seven studies found no significant difference between the serum vitamin D levels of BPPV patients and controls.[36 – 42] Amongst cases and controls with osteoporosis

For peer review only - http://bmjopen.bmj.com/site/about/guidelines.xhtml

and osteopenia, two studies[2 43] found no significant difference between cases and controls, although osteoporosis was correlated with low serum vitamin D in BPPV patients.[2] Serum vitamin D levels were also reported to be lower in recovering BPPV patients with residual dizziness, compared to those without.[44]

The relationship between BPPV recurrence and serum 25-hydroxyvitamin D was examined in 12 studies, with nine studies reporting lower serum vitamin D levels in patients with recurrent BPPV compared to those experiencing an initial attack[1 31 35 – 37 42 45 46] and three reporting no significant difference in vitamin D levels between the two groups.[38 47 48] Three studies[41 49 50] reported that vitamin D supplementation reduced the number of recurrent episodes of BPPV. Vitamin D supplementation was seen to improve the bone mineral density of BPPV patients in one study, however the effects on BPPV recurrence were not reported.[51]

Whether vitamin D levels differed between sub-types of BPPV was assessed in four studies, two of which found that serum 25-hydroxyvitamin D were lower in canalithiasis compared to cupulolithiasis[47 52] whilst the remaining two found no significant difference in vitamin levels between BPPV sub-types.[29 53] One study reported a much higher prevalence of vitamin D deficiency (<20ng/ml) in patients undergoing an acute attack of BPPV compared with 6 months post-episode (93.5% and 38.7% respectively).[54]

Seasonality and BPPV was examined by two included studies; Shu and colleagues[16] reported that the median number of BPPV referrals was higher in winter compared to summer months, whilst Califano *et al.*[36] found no seasonal patterns of BPPV cases and high levels of vitamin D deficiency and insufficiency in both winter and summer.

Meta-analysis

A total of 19 studies reported mean and standard deviation for serum 25-hydroxyvitamin D levels in case (BPPV) and control (non-BPPV) groups and were included in the meta-analysis (Supplementary 2, Table 2). These described a total of 7387 participants (2325 cases; 5062 controls), ranging in age from 18 to 88 years and sex distribution 0 to 100% male. Seven studies were excluded for the following reasons: four because the case and control groups both had BPPV,[44 46 49 51] two as standard deviation was not reported[28 41] and one as vitamin D levels were not given in ng/mL.[43] Combined mean averages and standard deviations were calculated for cases and controls using data provided in three studies[1 35 55] as these were initially reported according to groups (age, sex, BPPV recurrence).

Standard deviations were recalculated for one study[42] after consultation with a statistician as reported standard deviations were not congruent with reported means or confidence intervals. All included studies had 'uncertain' or 'high' risk of bias in at least one domain according to RoBANs, whilst two[36 37] were high risk in two domains. These were not excluded as the results of both were in accordance with the rest of the studies, and neither the trend or significance of the analysis was affected on removal of one or both studies from the analysis. Pooled standardised mean difference (SMD) indicated that serum vitamin D was on average lower in cases with BPPV compared to controls (SMD -2.07; 95% confidence interval -3.32, -0.81; $p = 0.001$ random effects model; **Figure 2**). There was substantial heterogeneity across included studies ($I^2 = 92\%$; $P < 0.00001$).

Seven studies reported mean and standard deviation for serum 25-hydroxyvitamin D levels in patient groups with recurrent and non-recurrent BPPV and were included in the meta-analysis (Supplementary 2, Table 3). These described a total of 622 participants (255 cases; 367 controls), ranging in age from 20 to 89 years and sex distribution from 0 to 35% male. Eight studies were excluded from the meta-analysis for the following reasons: three because no standard deviation was

For peer review only - http://bmjopen.bmj.com/site/about/guidelines.xhtml

reported,[28 47 56] three because serum vitamin D levels were not reported and two because vitamin D supplements were utilised.[36 49] All included studies were of ‘Good’ quality according to the NoS or had ‘uncertain’ or ‘high’ risk of bias in at least one domain according to RoBANs. Pooled standardised mean difference (SMD) indicated that serum vitamin D is no different in individuals with recurrent BPPV compared to those with non-recurrent disease (SMD -1.90; 95% confidence interval -3.80, 0.00; p = 0.05 random effects model; **Figure 3**). There was moderate heterogeneity across included studies ($I^2 = 66\%$; P = 0.007).

For peer review only

***Table 1*. Characteristics of included studies.** NOS = Newcastle Ottawa Scale (Wells et al 2018).

Author and year	Sample size	Study design	Country	Age range	Case Mean age (SD)	Case % male	Case 25(OH)D (ng/ml) mean (SD)	Quality
Bi *et al.* 2021	52 (27 case, 25 control)	Prospective Case-control	China	not reported	51.6 ± 15.8	33.33	14.64 ± 6.94*	n/a RoBANS
Califano *et al.* 2019	227 (127 case, 100 control)	Retro- + Pro-spective Case-control	Italy	not reported	60 ± 11.5**	40.2	24.4 ± 10.69	n/a RoBANS
Carneiro de Sousa *et al.* 2019	10 (5 case, 5 control)	Prospective Case-control	Portugal	52-82	64 ± 9.3 (case + control)	0	13.6 ± 5.7**	n/a RoBANS
Çelik *et al.* 2021	339 (190 case, 149 control)	Prospective Case-control	Turkey	not reported	43.7 ± 16	57.4	15.64 ± 8.4*	n/a RoBANS
Ceylan and Kanmaz 2020	197 (97 case, 100 control)	Retrospective Case-control	Turkey	not reported	57.07 ± 12.6	34	15.87 ± 8.6*	n/a RoBANS
Cheng et al. 2021	640 (320 case, 320 control	Prospective Case-control	China	not reported	68.2±6.02	45	23.2±4.09	n/a RoBANS
Çıkrıkçı Işık *et al.* 2017	127 (64 case, 63 control)	Retrospective Case-control	Turkey	not reported	56 ± 13.1	26.6	9.51 ± 5.49*	n/a RoBANS
Ding *et al.* 2019	522 (174 case, 348 control)	Prospective Case-control	China	not reported	Median reported	41.4	18.2 (n/a)*	n/a RoBANS
Elmoursey and Abbas 2021	60	Prospective Case-control	Egypt	24-70	46 ± 12.6	40	26.1 ± 11.7	n/a RoBANS
Goldschagg *et al.* 2021	459 (158 cases, 301 control)	Retrospective Case-control	Germany	not reported	61 ± 14	44.9	23.4 ± 9.4	n/a RoBANS
Gu, Dong and Gu 2018	100 (50 case, 50 control)	Prospective Case-control	China	not reported	51.36 ± 10.58	40	17.62 ± 9.0	n/a RoBANS
Han *et al.* 2018	165 (85 case, 80 control)	Retrospective Case-control	China	44-88	63.5 ± 9.72	0	19.1 ± 5.2	n/a RoBANS
Han *et al.* 2020	117	Retrospective Cohort	Republic of Korea	25-78	55 ± 11	22.2	24.47 ± 11.6**	Good
Han *et al.* 2021	201	Retrospective Cohort	China	not reported	65.14 ± 13.35**	0	19.74 ± 6.4**	Fair
Inan *et al.* 2021	104 (52 case, 52 control)	Retrospective Case-control	Turkey	18-80	55.6 (n/a)	44	15.3 ± 9.8	n/a RoBANS
Kahraman *et al.* 2016	37	Retrospective Cohort	Turkey	23–75	49.8 (n/a)	35.5	9.73 ± 8.8 (initial visit)*	Good
Karataş et al 2017	156 (78 case, 78 control)	Retrospective Case-control	Turkey	22-85	51.4 ± 12.2	37.2	23.0 ± 14.4	n/a RoBANS
Lee *et al.* 2017	132	Retrospective Cohort	Republic of Korea	49-81	63 ± 10.0	0	29.43 ± 14.2**	Fair
Maslovara *et al.* 2018	40	Prospective Cohort	Croatia	not reported	64.2 ± 12.1	28	21 ± 7.4	Good
Melis *et al.* 2020	73	Prospective Cohort	Italy	not reported	62.99 ± 14.6**	27.4**	22.13 ± 15.1**	Good
Nakada *et al.* 2019	35	Retrospective Cohort	Japan	not reported	76.36 ± 6.4**	34.3**	16.29 ± 3.9**	Poor
Parham *et al.* 2013	29 (16 case, 13 control)	Prospective Case-Control	USA	49-81	68.5 (n/a)	0	reported as nM/L	n/a RoBANS
Pecci *et al.* 2022	50 (26 case, 24 control)	Prospective Case-control	Italy	39-79	61.85 (n/a)	23	20.18 (SD not given)	n/a RoBANS
Sarsitthithim *et al.* 2021	137 (69 case, 68 control)	Prospective Case-Control	Thailand	39-89	61.4 ± 11.5	14.5	21.5 ± 5.3	n/a RoBANS
Sen, Padiyar and Arora 2018	200 (100 case, 100 control)	Prospective Case-control	India	18-60	48.6 ± 10.2	33	20.3 ± 12.2	n/a RoBANS
Shu *et al.* 2019	877	Retrospective Cohort	China	not reported	Median reported	not reported	18.03 (n/a)**	n/a RoBANS
Shin *et al.* 2023	50	Retrospective Cohort	Republic of Korea	not reported	50.5 ± 14.6**	28**	Reported by group	Good
Song *et al.* 2020	3505 (380 case, 3125 control)	Prospective Case-Control	China	19-85	50.7 ± 13.6**	25.5	14.24 ± 6.6**	Good
Talaat *et al.* 2015	180 (80 case, 100 control)	Prospective Case-Control	Kuwait	31-71	47.6 ± 9.1	35	14.19 ± 9.3**	n/a RoBANS
Thomas *et al.* 2021	98 (49 cases, 49 controls)	Prospective Case-control	India	21-71	44.39 (n/a)	32.7	21.3 ± 9.567***	n/a RoBANS
Wang *et al.* 2020	183 (103 case, 80 control)	Prospective Case-Control	China	50-86	63.0 ± 9.3	0	17.2 ± 2.0	n/a RoBANS
Wu *et al.* 2018	152 (60 case, 92 control)	Retrospective Case-control	China	not reported	59.35 ± 13.24	100	23.0 ± 6.8	n/a RoBANS
Wu *et al.* 2022	123 (51 case; 72 control)	Prospective Case-control	China	not reported	58.5 ± 12.6**	32.5	Median reported	n/a RoBANS
Yang *et al.* 2017	260 (130 case, 130 control)	Retrospective Case-control	Republic of Korea	20-87	54.9 ± 12.2	23.1	18.2 ± 10.3	n/a RoBANS
Zhang *et al.* 2022	206 (156 case, 50 control)	Prospective Case-control	China	46-75	59.5 ± 7.4	0	18.8 ± 2.5**	n/a RoBANS

Abbreviations: 25(OH)D = 25hydroxyvitamin D, BPPV= benign paroxysmal positional vertigo, SD= standard deviation, *= vitamin deficiency (≤20 ng/ml)
** Calculated combined average/SD. Combines groups of means and Standard Deviations into a Single Group by Repeating Cochrane's formula; *** SD recalculated using reported mean and confidence intervals

Discussion

Our systematic review and meta-analyses were based on a total of 35 articles; 26 case-control studies and nine cohort studies. It provides up-to-date information regarding the evidence base for vitamin D and benign paroxysmal positional vertigo (BPPV). The included studies described 9843 participants, of which 7510 were included in the meta-analyses. The studies included a broad range of populations from thirteen different countries in the Northern Hemisphere.

Study quality was variable, and the majority of included studies were identified as having a high risk of bias in at least one domain after application of the NOS or ROBANS tools, most commonly due to a lack of blinding of outcome assessment. Some of the identified studies in this systematic review were also from journals with unclear peer-review processes or non-impact journals. Bias and potential bias was also identified relating to participant selection and unknown confounding variables, for example participant occupation, unmatched case and control selection and incomplete data relating to relevant comorbidities including renal failure. Whilst it was not possible to complete meta-analysis using only high-quality studies, the authors are reassured that the findings from this review are comparable to previously published literature,[7 8] and did not find inclusion of studies with higher identified bias had any effect on analysis trend or significance.

The main findings of the meta-analysis were that individuals with BPPV had lower average serum 25-hydroxyvitamin D levels compared to controls, a finding which was statistically significant (P = 0.001). Examining the published literature to date, it is clear that the evidence base regarding the link between vitamin D and BPPV is mixed- with a number of studies included in this review reporting no significant difference between cases with BPPV and controls and others reporting lower serum vitamin D levels in patients with BPPV. These mixed results may reflect that the strength of the relationship between vitamin D and BPPV is relatively weak. Another possibility is that rather than vitamin D having a direct physiological role in the development of BPPV it is an indirect marker of another related biomarker, for example calcium; as calcitriol, manufactured from 25-hydroxyvitamin D, plays a role in regulating intestinal absorption of dietary calcium and bone mineralisation both directly and through interaction with parathyroid hormone.[57]

The effect size in our meta-analysis was relatively small (SMD -2.07), which aligns with other non-geographically restricted systematic reviews performed previously which have established an association between low serum vitamin D and BPPV incidence.[8 9] It remains unclear however, whether the small absolute difference in serum vitamin D concentration reported between patients with BPPV and those without is physiologically relevant.

The results of our meta-analysis into the association between BPPV recurrence and serum vitamin D levels indicated a similar (SMD -1.90), albeit non- significant association between BPPV recurrence and lower serum vitamin D levels. Previously published, non-geographically restricted systematic reviews have demonstrated a mixed picture relating to the relationship between serum vitamin D and BPPV recurrence with both a negative association,[8 10] or no impact on recurrence[11] reported.

It might have been expected that restricting our review to include only studies conducted in the northern hemisphere would result in a larger effect size, as more northerly latitudes have previously been shown to be a risk factor for vitamin D deficiency.[58 59] However, as vitamin D deficiency is more prevalent in such countries during the winter,[60] this may have been confounded by the fact that most of the included studies did not include temporal data relating to the month and/or seasons when vitamin D levels were measured or BPPV occurred. Some of the articles included in this review indicated mixed evidence for a potential relationship between serum vitamin D levels and sub-types of BPPV. Two articles[47 52] found lower serum vitamin D levels in canalithiasis compared to

For peer review only - http://bmjopen.bmj.com/site/about/guidelines.xhtml

cupulolithiasis, whilst a further two found no significant difference in vitamin D levels between the BPPV sub-types.[29 53] It was not possible to perform a meta-analysis on this effect due to the small number of studies investigating this relationship, however this could be a parameter for future studies investigating BPPV and vitamin D to explore further. Similarly, the potential association between BPPV, vitamin D and bone mineral density identified by some of the articles[2 43] could not be further analysed with meta-analysis but could be an area of interest for future research.

It was not possible to perform a meta-analysis examining the seasonality of both BPPV and vitamin D as only two studies reported vitamin D levels according to month or season.[16 36] This is an important potential confounding factor, as vitamin D levels have been observed to be at their lowest during months when the incidence of BPPV is greatest.[18] One hypothesis is that higher levels of vitamin D may offer a protective effect against BPPV during the summer months, and that the decrease in vitamin D levels over the winter months results in the increased incidence of BPPV seen during these months. Future research should separate individuals by month or season of admission (summer and winter-spring) and examine the difference in serum vitamin D levels between patients and controls during these time periods.

In conclusion, our results indicate that low serum vitamin D levels are correlated with the incidence of BPPV, whilst a non-significant association was reported in recurrent BPPV compared to non-recurrent disease. It was not possible to further investigate the seasonal relationships of BPPV and vitamin D deficiency as most studies do not include data relating to when patients are seen, and this is an area of interest for future research. Investigating if serum vitamin D levels relate to sub-types of BPPV and whether restoring vitamin D levels to sufficient levels via supplementation offers protection against BPPV are other topics which would benefit from further studies. As a large proportion of the population of countries in northern hemisphere countries are at a greater risk of vitamin D deficiency, patients developing BPPV in these countries may benefit from opportunistic serum vitamin D testing and/or supplementation.

For peer review only

For peer review only - http://bmjopen.bmj.com/site/about/guidelines.xhtml

Figure Legends:

Figure 1. *Flow diagram detailing results of searches and study selection process*

Figure 2. Relationship between serum 25-hydroxyvitamin D and Benign Paroxysmal Positional Vertigo incidence.

Figure 3. Relationship between serum 25-hydroxyvitamin D and Benign Paroxysmal Positional Vertigo recurrence.

For peer review only

Funding: KK was supported by the NIHR Manchester Biomedical Research Centre, UK IS-BRC-1215-20007, NIHR203308 and MRC Programme Grants MR/L003589/1, MR/V01272X/1.

Competing interests: None to declare

Data Sharing: No additional data.

Contributorship statement: HW, KK and GB conceptualised, planned, and designed this Systematic Review and Meta-Analyses. HW conducted the searches and initial screening of titles and abstracts. HW and GB independently reviewed the full texts and conducted the assessment of bias. HW extracted the data, and this was verified by GB, disagreements at any stage were resolved by discussion and by involvement of KK. HW and GB interpreted the results, and this was reviewed by KK. HW wrote the original manuscript. GB and KK were responsible for supervision, administration, reviewing, and editing the manuscript. All authors reviewed the results and approved the final version of the manuscript.

For peer review only

For peer review only - http://bmjopen.bmj.com/site/about/guidelines.xhtml

References

1. Talaat HS, Abuhadied G, Talaat AS, et al. Low bone mineral density and vitamin D deficiency in patients with benign positional paroxysmal vertigo. *European archives of oto-rhino-laryngology : official journal of the European Federation of Oto-Rhino-Laryngological Societies (EUFOS) : affiliated with the German Society for Oto-Rhino-Laryngology - Head and Neck Surgery* 2015;272(9):2249-53. doi: 10.1007/s00405-014-3175-3 [published Online First: 2014/06/30]
2. Lee SB, Lee CH, Kim YJ, et al. Biochemical markers of bone turnover in benign paroxysmal positional vertigo. *PloS one* 2017;12(5):e0176011. doi: 10.1371/journal.pone.0176011 [published Online First: 2017/05/04]
3. Büki B, Jünger H, Zhang Y, et al. The Price of Immune Responses and the Role of Vitamin D in the Inner Ear. *Otology & neurotology : official publication of the American Otological Society, American Neurotology Society [and] European Academy of Otology and Neurotology* 2019;40(6):701-09. doi: 10.1097/mao.0000000000002258 [published Online First: 2019/06/14]
4. Borel P, Caillaud D, Cano NJ. Vitamin D Bioavailability: State of the Art. *Critical Reviews in Food Science and Nutrition* 2015;55(9):1193-205. doi: 10.1080/10408398.2012.688897
5. Alshahrani F, Aljohani N. Vitamin D: deficiency, sufficiency and toxicity. *Nutrients* 2013;5(9):3605-16. doi: 10.3390/nu5093605 [published Online First: 20130913]
6. You P, Instrum R, Parnes L. Benign paroxysmal positional vertigo. *Laryngoscope investigative otolaryngology* 2019;4(1):116-23. doi: 10.1002/lio2.230 [published Online First: 2019/03/05]
7. Chen J, Zhao W, Yue X, et al. Risk Factors for the Occurrence of Benign Paroxysmal Positional Vertigo: A Systematic Review and Meta-Analysis. *Frontiers in neurology* 2020;11:506. doi: 10.3389/fneur.2020.00506 [published Online First: 2020/07/14]
8. Yang B, Lu Y, Xing D, et al. Association between serum vitamin D levels and benign paroxysmal positional vertigo: a systematic review and meta-analysis of observational studies. *European archives of oto-rhino-laryngology : official journal of the European Federation of Oto-Rhino-Laryngological Societies (EUFOS) : affiliated with the German Society for Oto-Rhino-Laryngology - Head and Neck Surgery* 2020;277(1):169-77. doi: 10.1007/s00405-019-05694-0 [published Online First: 2019/10/21]
9. AlGarni MA, Mirza AA, Althobaiti AA, et al. Association of benign paroxysmal positional vertigo with vitamin D deficiency: a systematic review and meta-analysis. *European archives of oto-rhino-laryngology : official journal of the European Federation of Oto-Rhino-Laryngological Societies (EUFOS) : affiliated with the German Society for Oto-Rhino-Laryngology - Head and Neck Surgery* 2018;275(11):2705-11. doi: 10.1007/s00405-018-5146-6 [published Online First: 2018/10/12]
10. Chen J, Zhang S, Cui K, et al. Risk factors for benign paroxysmal positional vertigo recurrence: a systematic review and meta-analysis. *Journal of neurology* 2020 doi: 10.1007/s00415-020-10175-0 [published Online First: 2020/08/26]
11. Li S, Wang Z, Liu Y, et al. Risk Factors for the Recurrence of Benign Paroxysmal Positional Vertigo: A Systematic Review and Meta-Analysis. *Ear, nose, & throat journal* 2022;101(3):NP112-NP34. doi: 10.1177/0145561320943362 [published Online First: 2020/08/11]
12. Sheikhzadeh M, Lotfi Y, Mousavi A, et al. Influence of supplemental vitamin D on intensity of benign paroxysmal positional vertigo: A longitudinal clinical study. *Caspian journal of internal medicine* 2016;7(2):93-8. [published Online First: 2016/07/08]
13. Yang Z, Li J, Zhu Z, et al. Effect of vitamin D supplementation on benign paroxysmal positional vertigo recurrence: A meta-analysis. *Science progress* 2021;104(2):368504211024569. doi: 10.1177/00368504211024569 [published Online First: 2021/06/16]
14. Cashman KD, Dowling KG, Škrabáková Z, et al. Vitamin D deficiency in Europe: pandemic? *The American journal of clinical nutrition* 2016;103(4):1033-44. doi: 10.3945/ajcn.115.120873 [published Online First: 2016/02/13]
15. Manios Y, Moschonis G, Lambrinou CP, et al. A systematic review of vitamin D status in southern European countries. *European journal of nutrition* 2018;57(6):2001-36. doi: 10.1007/s00394-017-1564-2 [published Online First: 2017/11/02]
16. Shu L, Wu J, Jiang CY, et al. Seasonal variation of idiopathic benign paroxysmal positional vertigo correlates with serum 25-hydroxyvitamin D levels: a six-year registry study in Shanghai, China. *Scientific reports* 2019;9(1):16230. doi: 10.1038/s41598-019-52803-4 [published Online First: 2019/11/09]
17. Sideris G, Sapountzi MC, Malamas V, et al. Climatic Variations as Indicators of Vitamin D Levels and Benign Paroxysmal Positional Vertigo. *Cureus* 2021;13(10):e18811. doi: 10.7759/cureus.18811 [published Online First: 2021/11/23]
18. Whitman GT, Baloh RW. Seasonality of benign paroxysmal positional vertigo. *JAMA otolaryngology-- head & neck surgery* 2015;141(2):188-9. doi: 10.1001/jamaoto.2014.2941 [published Online First: 2014/11/21]
19. Jeong J, Eo TS, Oh J, et al. Monthly and seasonal variations in benign paroxysmal positional vertigo. *Journal of vestibular research : equilibrium & orientation* 2021;31(2):101-07. doi: 10.3233/ves-200030 [published Online First: 2021/02/09]
20. Seidel DU, Park JJ, Sesterhenn AM, et al. Demographic data and seasonal variation in peripheral vestibular disorders in ENT practices in Germany. *Journal of vestibular research : equilibrium & orientation* 2019;29(4):181-90. doi: 10.3233/ves-190668 [published Online First: 2019/07/01]
21. Higgins JPT, Thomas J, Chandler J, et al. Cochrane Handbook for Systematic Reviews of Interventions version 6.4 (updated August 2023) 2023 [Available from: Available from www.training.cochrane.org/handbook.2023.

For peer review only - http://bmjopen.bmj.com/site/about/guidelines.xhtml

22. Page MJ, McKenzie JE, Bossuyt PM, et al. The PRISMA 2020 statement: an updated guideline for reporting systematic reviews. *BMJ* 2021;372:n71. doi: 10.1136/bmj.n71
23. Kim SY, Park JE, Lee YJ, et al. Testing a tool for assessing the risk of bias for nonrandomized studies showed moderate reliability and promising validity. *Journal of clinical epidemiology* 2013;66(4):408-14. doi: 10.1016/j.jclinepi.2012.09.016 [published Online First: 2013/01/23]
24. Wells GA, Shea B, O'Connell D, et al. The Newcastle-Ottawa Scale (NOS) for assessing the quality of nonrandomised studies in meta-analyses 2018 [Available from: http://www.ohri.ca/programs/clinical_epidemiology/oxford.asp accessed 21.05.2021 2021.
25. Holick MF. Vitamin D deficiency. *The New England journal of medicine* 2007;357(3):266-81. doi: 10.1056/NEJMra070553 [published Online First: 2007/07/20]
26. Bi J, Liu B, Zhang Y, et al. Study on the Bone Metabolism Indices and Otoconin-90 in Benign Paroxysmal Positional Vertigo. *Otology & neurotology : official publication of the American Otological Society, American Neurotology Society [and] European Academy of Otology and Neurotology* 2021;42(6):e744-e49. doi: 10.1097/mao.0000000000003087 [published Online First: 2021/02/20]
27. Çelik H, Yardim A, Ertaş A, et al. Evaluation of Serum 25-Hydroxyvitamin Vitamin D, Vitamin B12, and Folate Levels in Patients with Benign Paroxysmal Positional Vertigo. . *Eurasian Journal of Emergency Medicine* 2021;20(1):19-24. doi: 10.4274/eajem.galenos.2020.88942
28. Ding J, Liu L, Kong WK, et al. Serum levels of 25-hydroxy vitamin D correlate with idiopathic benign paroxysmal positional vertigo. *Bioscience reports* 2019;39(4) doi: 10.1042/bsr20190142 [published Online First: 2019/04/10]
29. Han K, Yun YM, Moon SG, et al. Bone mineral density and serum 25-hydroxyvitamin D in subtypes of idiopathic benign paroxysmal positional vertigo. *American journal of otolaryngology* 2020;41(1):102313. doi: 10.1016/j.amjoto.2019.102313 [published Online First: 2019/11/17]
30. Inan HC, Mertoğlu C, Erdur ZB. Investigation of Serum Calcium and 25-Hydroxy Vitamin D Levels in Benign Paroxysmal Positional Vertigo Patients. *Ear, nose, & throat journal* 2021;100(9):643-46. doi: 10.1177/0145561321989451 [published Online First: 2021/01/26]
31. Sarsitthithum K, Wisupagan T, Kiatthanabumrung S, et al. The Association Between Serum Vitamin D Levels and Benign Paroxysmal Positional Vertigo. *Ear, nose, & throat journal* 2021:1455613211008561. doi: 10.1177/01455613211008561 [published Online First: 2021/04/20]
32. Sen K, Padiyar BV, Arora G. Association of Benign Paroxysmal Positional Vertigo with Osteoporosis and Vitamin D Deficiency – A Case-Control Study. *Dubai Medical Journal* 2018;1:2-5. doi: 10.1159/000492756
33. Wang Z, Yao G, Tao X, et al. Evaluation of bone mineral density and 25-(OH) vitamin D levels in middle-aged and elderly women with recurrent benign paroxysmal positional vertigo. *Acta oto-laryngologica* 2020;140(2):89-93. doi: 10.1080/00016489.2019.1692146 [published Online First: 2019/12/27]
34. Wu Y, Fan Z, Jin H, et al. Assessment of Bone Metabolism in Male Patients With Benign Paroxysmal Positional Vertigo. *Frontiers in neurology* 2018;9:742. doi: 10.3389/fneur.2018.00742 [published Online First: 2018/09/21]
35. Zhang X, Zhang Z, Lv X. Predictive values of serum estradiol, calcium, and 25-hydroxyvitamin D levels for recurrence of benign paroxysmal positional vertigo in postmenopausal women. *Turk J Phys Med Rehabil* 2022;68(1):30-36. doi: 10.5606/tftrd.2022.5964 [published Online First: 20220301]
36. Califano L, Salafia F, Melillo MG, et al. Is hypovitaminosis D a risk factor for either the onset or the recurrence of Benign Paroxysmal Positional Vertigo? . *Frontiera ORL* 2019
37. Ceylan SM, Kanmaz MA. Vitamin D Influence on the Clinical Course of Benign Paroxysmal Positional Vertigo: A Retrospective Cohort Study. *Otorhinolaryngology Clinics: An International Journal* 2020;12(3):43-47.
38. Çikrikçi Işik G, Cevik Y, Emektar E, et al. Analysis of Vitamin D and Calcium Levels in Benign Paroxysmal Positional Vertigo. *Eurasian Journal of Emergency Medicine* 2017;16:128-32. doi: 10.5152/eajem.2017.58077
39. Goldschagg N, Teupser D, Feil K, et al. No evidence for a specific vitamin D deficit in benign paroxysmal positional vertigo. *European journal of neurology* 2021;28(9):3182-86. doi: 10.1111/ene.14980 [published Online First: 2021/06/17]
40. Karataş A, Acar Yuceant G, Yuce T, et al. Association of Benign Paroxysmal Positional Vertigo with Osteoporosis and Vitamin D Deficiency: A Case Controlled Study. *The journal of international advanced otology* 2017;13(2):259-65. doi: 10.5152/iao.2016.2640 [published Online First: 2017/03/10]
41. Pecci R, Mandalà M, Marcari A, et al. Vitamin D Insufficiency/Deficiency in Patients with Recurrent Benign Paroxysmal Positional Vertigo. *The journal of international advanced otology* 2022;18(2):158-66. doi: 10.5152/iao.2022.21269
42. Thomas RJ, Goutham MK, Bhat VS, et al. Association of Serum Calcium and Vitamin D with Benign Paroxysmal Positional Vertigo. *Int Arch Otorhinolaryngol* 2022;26(3):e365-e69. doi: 10.1055/s-0041-1724093 [published Online First: 20211103]
43. Parham K, Leonard G, Feinn RS, et al. Prospective clinical investigation of the relationship between idiopathic benign paroxysmal positional vertigo and bone turnover: a pilot study. *The Laryngoscope* 2013;123(11):2834-9. doi: 10.1002/lary.24162 [published Online First: 2013/05/16]
44. Wu Y, Han K, Han W, et al. Low 25-Hydroxyvitamin D Levels Are Associated With Residual Dizziness After Successful Treatment of Benign Paroxysmal Positional Vertigo. *Frontiers in neurology* 2022;13:915239. doi: 10.3389/fneur.2022.915239 [published Online First: 20220622]

For peer review only - http://bmjopen.bmj.com/site/about/guidelines.xhtml

45. Melis A, Rizzo D, Gallus R, et al. Relationship between calcium metabolism and benign paroxysmal positional vertigo in north Sardinia population. *Journal of vestibular research : equilibrium & orientation* 2020;30(6):375-82. doi: 10.3233/ves-200025
46. Shin HI, Park Y, Lee HJ, et al. Correlation between serum vitamin D level and benign paroxysmal positional vertigo recurrence. *Auris Nasus Larynx* 2023 doi: 10.1016/j.anl.2022.12.017 [published Online First: 20230123]
47. Maslovara S, Butkovic Soldo S, Sestak A, et al. 25 (OH) D3 levels, incidence and recurrence of different clinical forms of benig paroxysmal positional vertigo. *Brazilian journal of otorhinolaryngology* 2018;84(4):453-59. doi: 10.1016/j.bjorl.2017.05.007 [published Online First: 2017/06/29]
48. Yang CJ, Kim Y, Lee HS, et al. Bone mineral density and serum 25-hydroxyvitamin D in patients with idiopathic benign paroxysmal positional vertigo. *Journal of vestibular research : equilibrium & orientation* 2018;27(5-6):287-94. doi: 10.3233/ves-170625 [published Online First: 2018/02/06]
49. Carneiro de Sousa PJM, Abreu Pereira DM, Carneiro Melo Pereira de Magalhães P, et al. Vitamin D deficiency and benign paroxysmal positioning vertigo. *Hearing, Balance and Communication* 2019;17(2):179-81. doi: 10.1080/21695717.2019.1590988
50. Elmoursy MM, Abbas AS. The role of low levels of vitamin D as a co-factor in the relapse of benign paroxysmal positional vertigo (BPPV). *American journal of otolaryngology* 2021;42(6):103134. doi: 10.1016/j.amjoto.2021.103134 [published Online First: 2021/06/25]
51. Gu X, Dong F, Gu J. Analysis of effect of 1α-hydroxyvitamin D3 on benign paroxysmal positional vertigo and risk factors. *Experimental and therapeutic medicine* 2018;15(3):2321-26. doi: 10.3892/etm.2018.5699 [published Online First: 2018/02/20]
52. Nakada T, Sugiura S, Uchida Y, et al. Difference in Serum Levels of Vitamin D Between Canalolithiasis and Cupulolithiasis of the Horizontal Semicircular Canal in Benign Paroxysmal Positional Vertigo. *Frontiers in neurology* 2019;10:176. doi: 10.3389/fneur.2019.00176 [published Online First: 2019/03/19]
53. Han WW, Wu YQ, Fan ZY, et al. Characteristics of bone metabolism in postmenopausal female patients with different types of idiopathic benign paroxysmal positional vertigo: A single-centre retrospective study. *American journal of otolaryngology* 2021;42(6):103149. doi: 10.1016/j.amjoto.2021.103149 [published Online First: 2021/07/10]
54. Kahraman SS, Ozcan O, Arli C, et al. Calcium Homeostasis During Attack and Remission in Patients With Idiopathic Benign Paroxysmal Positional Vertigo. *Otology & neurotology : official publication of the American Otological Society, American Neurotology Society [and] European Academy of Otology and Neurotology* 2016;37(9):1388-92. doi: 10.1097/mao.0000000000001167 [published Online First: 2016/08/16]
55. Song P, Zhao X, Xu Y, et al. Correlation Between Benign Paroxysmal Positional Vertigo and 25-hydroxyvitamin D. *Frontiers in neurology* 2020;11:576. doi: 10.3389/fneur.2020.00576 [published Online First: 2020/07/14]
56. Rhim GI. Serum Vitamin D and Long-term Outcomes of Benign Paroxysmal Positional Vertigo. *Clin Exp Otorhinolaryngol* 2019;12(3):273-78. doi: 10.21053/ceo.2018.00381 [published Online First: 20190301]
57. Fleet JC. The role of vitamin D in the endocrinology controlling calcium homeostasis. *Molecular and cellular endocrinology* 2017;453:36-45. doi: 10.1016/j.mce.2017.04.008 [published Online First: 2017/04/13]
58. Leary PF, Zamfirova I, Au J, et al. Effect of Latitude on Vitamin D Levels. *J Am Osteopath Assoc* 2017;117(7):433-39. doi: 10.7556/jaoa.2017.089
59. Oskarsson V, Eliasson M, Salomaa V, et al. Influence of geographical latitude on vitamin D status: cross-sectional results from the BiomarCaRE consortium. *British Journal of Nutrition* 2022;128(11):2208-18. doi: 10.1017/S0007114521005080 [published Online First: 2021/12/22]
60. Huotari A, Herzig KH. Vitamin D and living in northern latitudes--an endemic risk area for vitamin D deficiency. *International journal of circumpolar health* 2008;67(2-3):164-78. doi: 10.3402/ijch.v67i2-3.18258 [published Online First: 2008/09/05]

For peer review only - http://bmjopen.bmj.com/site/about/guidelines.xhtml

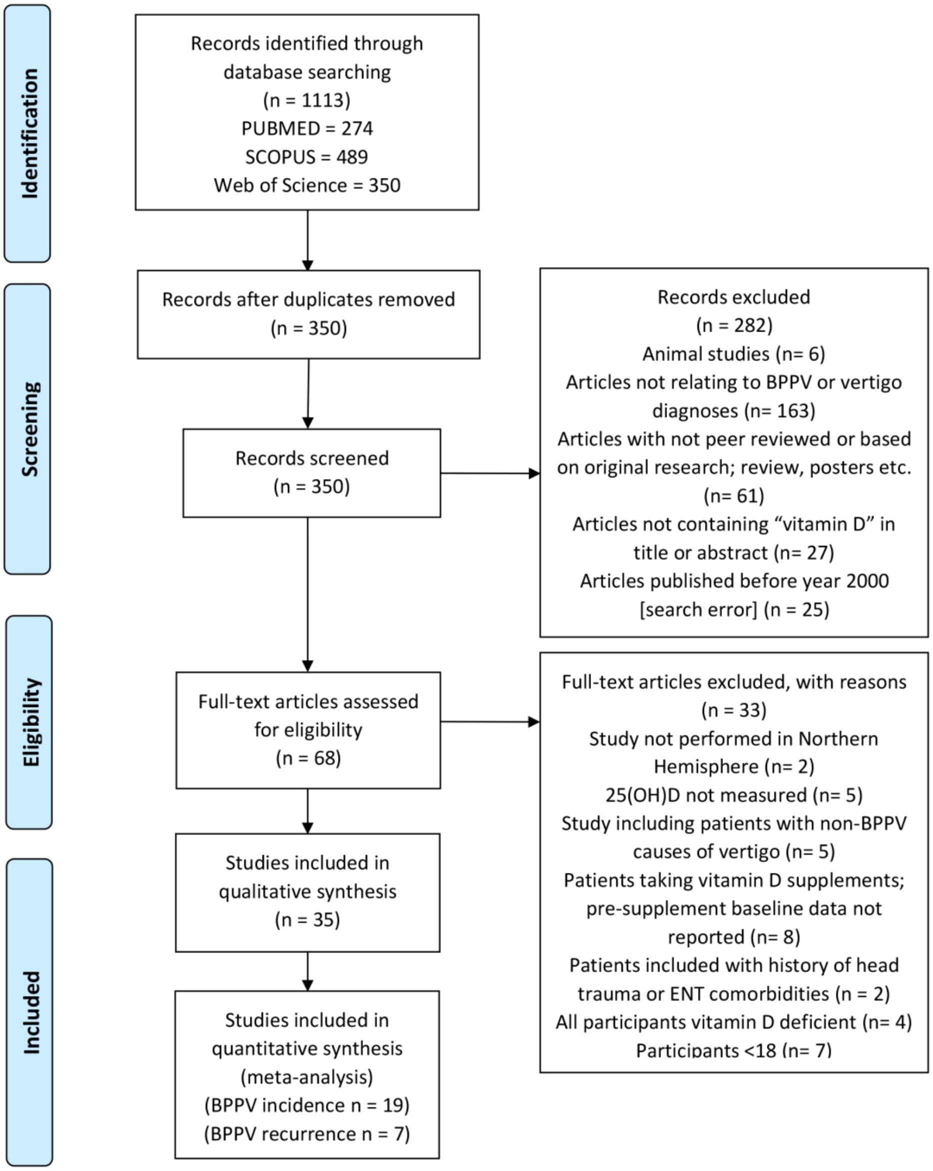

Figure 1. Flow diagram detailing results of searches and study selection process

171x216mm (300 x 300 DPI)

	BPPV			Control				Mean Difference
Study or Subgroup	Mean	SD	Total	Mean	SD	Total	Weight	IV, Random, 95% CI
Bi et al 2021	14.64	6.94	27	19.56	6.55	25	4.2%	-4.92 [-8.59, -1.25]
Califano et al 2019	21.08	11	127	25.6	10.5	100	4.9%	-4.52 [-7.33, -1.71]
Çelik et al 2021	15.64	8.4	190	16.29	6.7	149	5.9%	-0.65 [-2.26, 0.96]
Ceylan and Kanmaz 2020	15.87	8.61	97	15.82	10.23	100	5.1%	0.05 [-2.59, 2.69]
Cheng et al 2021	23.2	4.09	320	25.8	3.43	320	6.5%	-2.60 [-3.18, -2.02]
Çıkrıkçı Işık et al 2017	9.51	5.49	64	11.02	9.62	63	5.0%	-1.51 [-4.24, 1.22]
Goldschagg et al 2021	23.4	9.4	158	21.5	10.6	301	5.7%	1.90 [0.01, 3.79]
Han et al 2018	19.1	5.2	85	22.5	5.8	80	5.9%	-3.40 [-5.08, -1.72]
Inan et al 2021	15.3	9.8	52	20.2	14.3	52	3.4%	-4.90 [-9.61, -0.19]
Karatas et al 2017	23	14.4	78	17	12.3	78	3.8%	6.00 [1.80, 10.20]
Sarsitthithim et al 2021	21.5	5.3	69	26.3	6.8	68	5.6%	-4.80 [-6.84, -2.76]
Sen, Padiyar and Arora 2018	20.3	12.2	100	18	10.1	100	4.7%	2.30 [-0.80, 5.40]
Song et al 2020	14.24	6.6	380	15.45	7.7	3125	6.4%	-1.21 [-1.93, -0.49]
Talaat et al 2015	14.19	9.3	80	19.53	8.45	100	5.1%	-5.34 [-7.97, -2.71]
Thomas et al 2021	21.3	9.567128	49	17.6	8.061036	49	4.3%	3.70 [0.20, 7.20]
Wang et al 2020	17.15	2.03	103	23.85	3.13	80	6.4%	-6.70 [-7.49, -5.91]
Wu et al 2018	20.99	6.76	60	23.17	6.49	92	5.5%	-2.18 [-4.34, -0.02]
Yang et al 2017	18.21	10.3	130	20	8.1	130	5.4%	-1.79 [-4.04, 0.46]
Zhang et al 2022	18.8	2.5	156	24	2.2	50	6.4%	-5.20 [-5.93, -4.47]
**Total (95% CI)**			**2325**			**5062**	**100.0%**	**-2.07 [-3.32, -0.81]**

Heterogeneity: $Tau^2 = 6.22$; $Chi^2 = 238.02$, df = 18 ($P < 0.00001$); $I^2 = 92\%$
Test for overall effect: Z = 3.24 (P = 0.001)

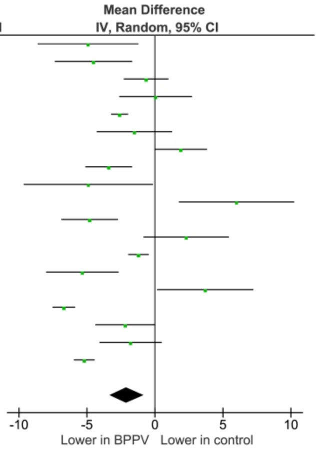

Figure 2. Relationship between serum 25-hydroxyvitamin D and Benign Paroxysmal Positional Vertigo incidence.

321x146mm (300 x 300 DPI)

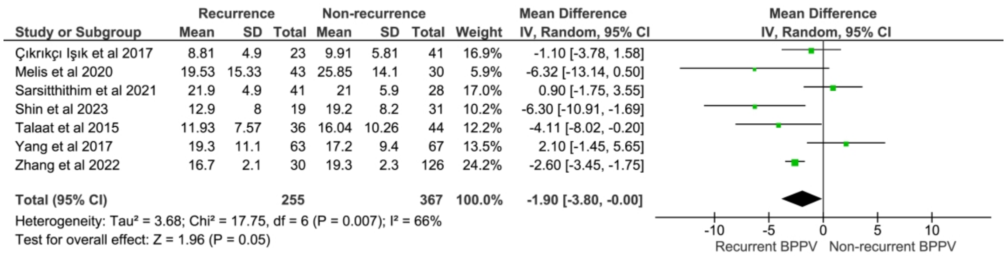

Figure 3. Relationship between serum 25-hydroxyvitamin D and Benign Paroxysmal Positional Vertigo recurrence.

303x78mm (300 x 300 DPI)

Association between vitamin D deficiency and benign paroxysmal positional vertigo: a systematic review and meta-analysis

Citation

Heather Wood, Ghada BinKhamis, Karolina Kluk De-Kort. Association between vitamin D deficiency and benign paroxysmal positional vertigo: a systematic review and meta-analysis. PROSPERO 2021 CRD42021271840 Available from: https://www.crd.york.ac.uk/prospero/display_record.php?ID=CRD42021271840

Review question

How do serum vitamin D levels affect the incidence and recurrence rates of benign paroxysmal positional vertigo (BPPV) in adult patients in the Northern Hemisphere?

Searches

We will search the following electronic bibliographic databases: PubMed, NICE, Scopus, Web of Science using the following search terms: "Vitamin D* OR 25-hydroxyvitamin D OR ergocalciferol OR cholecalciferol AND benign paroxysmal positional vertigo", "Vitamin D* OR 25-hydroxyvitamin D OR ergocalciferol OR cholecalciferol AND BPPV" "Vitamin D* OR 25-hydroxyvitamin D OR ergocalciferol OR cholecalciferol AND vertigo"

Research papers published between 2000 and 2021 will be reviewed and language is restricted with English.

- Searches will be re-run prior to the final analysis to identify any further studies.

- Unpublished studies will not be sought

Types of study to be included

Randomised controlled trial, cohort study or case-controlled study

Condition or domain being studied

Benign Paroxysmal positional vertigo (BPPV) and serum vitamin D levels

Participants/population [2 changes]

Inclusion criteria:

- Participants over the age of 18 with at least one episode of diagnosed benign paroxysmal positional vertigo (BPPV).

- Serum 25-hydroxyvitamin D levels measured and reported.

- Geographical location of study participants in Northern hemisphere.

- Participants not taking vitamin D supplements,

- No other comorbid diagnoses.

Exclusion criteria:

- Participants below the age of 18 with at least one episode of BPPV.

- Serum 25-hydroxyvitamin D levels not measured or reported.

- Geographical location of study participants not in Northern hemisphere.

- Participants taking vitamin D supplements,

- Participants with other comorbid diagnoses.

Intervention(s), exposure(s)

Serum 25-hydroxyvitamin D levels

Comparator(s)/control

Normal controls over the age of 18 with no past medical history of benign paroxysmal positional vertigo

Context

Studies in hospital ENT outpatient departments. Research including participants in southern hemisphere countries will be excluded

Main outcome(s)

Incidence of benign paroxysmal positional vertigo. Recurrence of benign paroxysmal positional vertigo

Measures of effect

Difference in average serum vitamin D levels between patients with new or recurrent benign paroxysmal positional vertigo compared to controls.

Additional outcome(s)

Not applicable

Data extraction (selection and coding)

Study selection: one author will select and this selection will be reviewed by two authors.

The final decision will be recorded in an excel spreadsheet.

Any disagreements will be resolved by discussion between the authors.

Extracted data will include:

Study design; country; author and published year; mean age of participants; range of participant age; sample size; disease diagnosis; percentage male participants and serum vitamin D level for cases and controls.

Risk of bias (quality) assessment

For peer review only

Any randomised controlled trials will be assessed using the Cochrane Collaboration's tool for assessing risk of bias in randomised trials.

Case-control studies will be assessed using the Risk of Bias Assessment Tool for Nonrandomized Studies (RoBANS) tool.

Cohort studies will be assessed according to the Newcastle Ottawa Scale (NOS).

Records of risk of bias will be recorded in Microsoft Word and Microsoft Excel files.

Strategy for data synthesis

Standardised mean difference (SMD) with a 95% confidence interval will be used to calculate the level of vitamin D in case and control groups. Plasma vitamin D levels of greater than 20 ng/ml will be considered to be sufficient. The Q statistic will be used to examine heterogeneity between studies, with P<0.10 regarded as significant. The random-effects model will be employed for the meta-analyses if P<0.10, otherwise the fixed-effect model will be used instead.

Statistical analysis will be completed using RevMan software version 5.4.1

Analysis of subgroups or subsets

Vitamin D status will be categorised into "sufficient" if serum levels are >20ng/l; or "insufficient" if serum levels <20ng/L

There may be differences between patients experiencing recurrent BPPV and those experiencing single episodes. Recurrent BPPV will be classified as symptom recurrence within 3 years of initial diagnosis.

Contact details for further information

Heather Wood

hmw998@student.bham.ac.uk

Organisational affiliation of the review

University of Birmingham

Review team members and their organisational affiliations [1 change]

Ms Heather Wood. University of Birmingham, Birmingham, UK

Dr Ghada BinKhamis. University of Manchester, Manchester, UK. King Fahad Medical City, Riyadh, Saudi Arabia

Dr Karolina Kluk De-Kort. University of Manchester, Manchester, UK

Type and method of review

Meta-analysis, Systematic review

Anticipated or actual start date

09 August 2021

For peer review only

Anticipated completion date [1 change]

01 September 2023

Funding sources/sponsors

None

Conflicts of interest

Language

English

Country

England

Stage of review

Review Ongoing

Subject index terms status

Subject indexing assigned by CRD

Subject index terms

Benign Paroxysmal Positional Vertigo; Humans; Vitamin D Deficiency

Date of registration in PROSPERO

06 August 2021

Date of first submission

04 August 2021

Stage of review at time of this submission [3 changes]

For peer review only

NIHR | National Institute for Health Research

PROSPERO
International prospective register of systematic reviews

Stage	Started	Completed
Preliminary searches	Yes	Yes
Piloting of the study selection process	Yes	Yes
Formal screening of search results against eligibility criteria	Yes	Yes
Data extraction	Yes	No
Risk of bias (quality) assessment	Yes	Yes
Data analysis	Yes	No

Revision note

Updating progress to date - checked boxes to indicate that screening and assessment of bias are complete and that data extraction and analysis have been started.

The record owner confirms that the information they have supplied for this submission is accurate and complete and they understand that deliberate provision of inaccurate information or omission of data may be construed as scientific misconduct.

The record owner confirms that they will update the status of the review when it is completed and will add publication details in due course.

For peer review

Versions

06 August 2021

09 August 2021

14 September 2021

27 October 2021

21 February 2023

01 April 2023

Supplementary 2

Supplementary 2, Table 1. Systematic review searches performed and results.

Search	*Search terms*	*Database*	*Results*	*Filters*
**1**	(Vitamin D OR 25-hydroxyvitamin D OR ergocalciferol OR cholecalciferol) AND BPPV	PUBMED	86	Years 2000 - 2023, English Language
**2**	(Vitamin D OR 25-hydroxyvitamin D OR ergocalciferol OR cholecalciferol) AND "Benign paroxysmal positional vertigo"	PUBMED	86	Years 2000 - 2023, English Language
**3**	(Vitamin D OR 25-hydroxyvitamin D OR ergocalciferol OR cholecalciferol) AND vertigo	PUBMED	102	Years 2000 - 2023, English Language
**4**	(Vitamin D* OR 25-hydroxyvitamin D OR ergocalciferol OR cholecalciferol) AND BPPV	SCOPUS	85	Year >1999, English Language
**5**	(Vitamin D* OR 25-hydroxyvitamin D OR ergocalciferol OR cholecalciferol) AND "Benign paroxysmal positional vertigo"	SCOPUS	104	Year >1999, English Language
**6**	(Vitamin D* OR 25-hydroxyvitamin D OR ergocalciferol OR cholecalciferol) AND "vertigo"	SCOPUS	300	Year >1999, English Language
**7**	(ALL=(Vitamin D OR 25-hydroxyvitamin D OR ergocalciferol OR cholecalciferol)) AND ALL=(BPPV)	Web of Science	101	Timespan: 2000-01-01 to2023-02-18, English Language
**8**	(ALL=(Vitamin D OR 25-hydroxyvitamin D OR ergocalciferol OR cholecalciferol)) AND ALL=(Benign Paroxysmal Positional Vertigo)	Web of Science	108	Timespan: 2000-01-01 to 2023-02-18, English Language
**9**	(ALL=(Vitamin D OR 25-hydroxyvitamin D OR ergocalciferol OR cholecalciferol)) AND ALL=(Vertigo)	Web of Science	141	Timespan: 2000-01-01 to2023-02-18, English Language

For peer review only

Supplementary 2, Table 2. Data extracted from included studies for the Meta-analysis exploring the relationship between Vitamin D deficiency and BPPV occurrence.

	Participants with BPPV			Control participants (without BPPV)		
**Study**	**Number of Participants**	**Vitamin D (Mean: ng/mL)**	**Vitamin D (SD: ng/mL)**	**Number of Participants**	**Vitamin D (Mean: ng/mL)**	**Vitamin D (SD: ng/mL)**
Bi et al. 2021	27	14.64	6.94	25	19.56	6.55
Califano et al. 2019	127	21.08	11	100	25.6	10.5
Çelik et al. 2021	190	15.64	8.4	149	16.29	6.7
Ceylan and Kanmaz 2020	97	15.87	8.61	100	15.82	10.23
Cheng et al. 2021	320	23.2	4.09	320	25.8	3.43
Çıkrıkçı Işık et al. 2017	64	9.51	5.49	63	11.02	9.62
Goldschagg et al. 2021	158	23.4	9.4	301	21.5	10.6
Han et al. 2018	85	19.1	5.2	80	22.5	5.8
Inan et al. 2021	52	15.3	9.8	52	20.2	14.3
Karataş et al. 2017	78	23	14.4	78	17	12.3
Sarsitthithim et al. 2021	69	21.5	5.3	68	26.3	6.8
Sen, Padiyar and Arora 2018	100	20.3	12.2	100	18	10.1
Song et al. 2020	380	14.24	6.6	3125	15.45	7.7
Talaat et al. 2015	80	14.19	9.3	100	19.53	8.45
Thomas et al. 2021	49	21.3	9.567	49	17.6	8.061
Wang et al. 2020	103	17.15	2.03	80	23.85	3.13
Wu et al. 2018	60	20.99	6.76	92	23.17	6.49
Yang et al. 2017	130	18.21	10.3	130	20	8.1
Zhang et al. 2022	156	18.8	2.5	50	24	2.2

For peer review only

Supplementary 2, Table 3. Data extracted from included studies for the Meta-analysis exploring the relationship between Vitamin D deficiency and BPPV recurrence.

Study	Participants with BPPV Recurrence			Participants without BPPV Recurrence		
	**Number of Participants**	**Vitamin D (Mean: ng/mL)**	**Vitamin D (SD: ng/mL)**	**Number of Participants**	**Vitamin D (Mean: ng/mL)**	**Vitamin D (SD: ng/mL)**
Çıkrıkçı Işık et al. 2017	23	8.81	4.9	41	9.91	5.81
Melis et al. 2020	43	19.53	15.33	30	25.85	14.1
Sarsitthithim et al. 2021	41	21.9	4.9	28	21	5.9
Shin *et al.* 2023	19	12.9	8	31	19.2	8.2
Talaat et al. 2015	36	11.93	7.57	44	16.04	10.26
Yang et al. 2017	63	19.3	11.1	67	17.2	9.4
Zhang *et al.* 2022	30	16.7	2.1	126	19.3	2.3

For peer review only

	Selection of Participants	Confounding Variables	Intervention measurement	Blinding of outcome assessment	Incomplete outcome data	Selective outcome reporting
Bi *et al.* 2021	-	?	-	+	-	-
Califano *et al.* 2019	?	+	-	+	-	-
Carneiro de Sousa *et al.* 2019	-	+	-	+	-	-
Çelik *et al*. 2021	-	-	-	+	-	-
Ceylan 2020	-	+	-	+	-	-
Cheng *et al*. 2021	-	?	-	+	-	-
Çıkrıkçı Işık *et al.* 2017	-	-	-	+	-	-
Ding *et al.* 2019	-	-	-	+	-	-
Elmoursey and Abbas 2021	-	+	-	+	-	-
Goldschlagg *et al.* 2021	-	?	-	+	-	-
Gu, Dong and Gu 2018	?	-	-	-	-	-
Han *et al.* 2018	-	?	-	+	-	-
Inan *et al.* 2021	?	-	-	+	-	-
Karatas *et al.* 2016	?	-	-	+	-	-
Parham *et al.* 2013	?	-	-	-	-	-
Pecci *et al.* 2022	+	-	-	+	-	-
Sarsitthithim *et al.* 2021	?	?	-	+	-	-
Sen, Padiyar and Arora 2018	?	?	-	+	-	-
Song *et al*. 2020	-	-	-	+	-	-
Talaat *et al*. 2015	-	-	-	+	-	-
Thomas *et al*. 2021	-	?	-	+	-	-
Wang *et al*. 2020	?	-	-	+	-	-
Wu *et al*. 2018	-	-	-	+	-	-
Wu *et al*. 2022	-	-	-	+	-	-
Yang *et al*. 2017	?	-	-	+	-	-
Zhang *et al*. 2022	-	-	-	+	-	-

Supplementary 2, Figure 1. Assessment of bias for case-control studies according to RoBANs.
Green '-' symbols= low risk of bias, red '+' symbols= high risk of bias and purple '?' symbols = unclear risk of bias.

	Representativeness of exposed cohort	Selection of non-exposed cohort	Ascertainment of exposure	Outcome of interest not present at start	Comparability of cohorts/confounders	Assessment of outcome	Follow-up long enough?	Adequacy of follow-up of cohorts	Study Quality
Han *et al.* 2020	*	*	*	-	**	*	*	n/a	Good
Han *et al*. 2021	-	*	*	-	**	*	*	n/a	Fair
Kahraman *et al.* 2016	*	*	*	-	*	*	*	*	Good
Lee *et al.* 2017	-	*	*	-	**	*	*	n/a	Fair
Maslovara *et al.* 2018	*	*	*	*	**	*	*	*	Good
Melis *et al.* 2020	*	*	*	*	**	*	-	*	Good
Nakada *et al*. 2019	*	*	*	-	-	*	*	n/a	Poor
Shin *et al*. 2023	*	*	*	-	*	*	*	*	Good
Shu *et al*. 2019	*	*	*	-	**	*	*	n/a	Good

Supplementary 2, Figure 2. Assessment of bias of cohort studies using the Newcastle-Ottawa Quality Assessment form for cohort studies.
'*' symbols indicate low risk of bias for a given criteria, '-' symbols indicate the study does not meet criteria for low risk of bias and 'n/a' indicates that the criterion does not apply to a given study (for example retrospective studies do not have a follow-up period by design).

Supplementary 2, Table 4. PRISMA Checklist

Section and Topic	Item #	Checklist item	Location where item is reported
**TITLE**			
Title	1	Identify the report as a systematic review.	Title page (p1)
**ABSTRACT**			
Abstract	2	See the PRISMA 2020 for Abstracts checklist.	Abstract (p2)
**INTRODUCTION**			
Rationale	3	Describe the rationale for the review in the context of existing knowledge.	Introduction (p4)
Objectives	4	Provide an explicit statement of the objective(s) or question(s) the review addresses.	Introduction (p4)
**METHODS**			
Eligibility criteria	5	Specify the inclusion and exclusion criteria for the review and how studies were grouped for the syntheses.	Methods: Inclusion and Exclusion criteria (p5)
Information sources	6	Specify all databases, registers, websites, organisations, reference lists and other sources searched or consulted to identify studies. Specify the date when each source was last searched or consulted.	Methods: Data sources and search strategy (p5)
Search strategy	7	Present the full search strategies for all databases, registers and websites, including any filters and limits used.	Methods: Data sources and search strategy (p5)
Selection process	8	Specify the methods used to decide whether a study met the inclusion criteria of the review, including how many reviewers screened each record and each report retrieved, whether they worked independently, and if applicable, details of automation tools used in the process.	Methods: Inclusion and Exculsion criteria (p5)
Data collection process	9	Specify the methods used to collect data from reports, including how many reviewers collected data from each report, whether they worked independently, any processes for obtaining or confirming data from study investigators, and if applicable, details of automation tools used in the process.	Methods: Data Extraction (p6)
Data items	10a	List and define all outcomes for which data were sought. Specify whether all results that were compatible with each outcome domain in each study were sought (e.g. for all measures, time points, analyses), and if not, the methods used to decide which results to collect.	Methods: Data Extraction (p6)
	10b	List and define all other variables for which data were sought (e.g. participant and intervention characteristics, funding sources). Describe any assumptions made about any missing or unclear information.	Methods: Data Extraction (p6)
Study risk of bias assessment	11	Specify the methods used to assess risk of bias in the included studies, including details of the tool(s) used, how many reviewers assessed each study and whether they worked independently, and if applicable, details of automation tools used in the process.	Methods: Assessment of bias (p6)
Effect measures	12	Specify for each outcome the effect measure(s) (e.g. risk ratio, mean difference) used in the synthesis or presentation of results.	Methods: Synthesis methods (p6)
Synthesis methods	13a	Describe the processes used to decide which studies were eligible for each synthesis (e.g. tabulating the study intervention characteristics and comparing against the planned groups for each synthesis (item #5)).	Methods: Synthesis methods (p6)
	13b	Describe any methods required to prepare the data for presentation or synthesis, such as handling of missing summary statistics, or data conversions.	Methods: Synthesis methods (p6)

For peer review only - http://bmjopen.bmj.com/site/about/guidelines.xhtml

Section and Topic	Item #	Checklist item	Location where item is reported
	13c	Describe any methods used to tabulate or visually display results of individual studies and syntheses.	Methods: Synthesis methods (p6)
	13d	Describe any methods used to synthesize results and provide a rationale for the choice(s). If meta-analysis was performed, describe the model(s), method(s) to identify the presence and extent of statistical heterogeneity, and software package(s) used.	Methods: Synthesis methods (p6)
	13e	Describe any methods used to explore possible causes of heterogeneity among study results (e.g. subgroup analysis, meta-regression).	Not applicable
	13f	Describe any sensitivity analyses conducted to assess robustness of the synthesized results.	Not applicable
Reporting bias assessment	14	Describe any methods used to assess risk of bias due to missing results in a synthesis (arising from reporting biases).	Not applicable
Certainty assessment	15	Describe any methods used to assess certainty (or confidence) in the body of evidence for an outcome.	Not applicable
**RESULTS**			
Study selection	16a	Describe the results of the search and selection process, from the number of records identified in the search to the number of studies included in the review, ideally using a flow diagram.	Results: Study selection (p6-7); Figure 1. Flow diagram detailing results of searches and study selection process; Supplementary 2. Systematic review searches performed and results
	16b	Cite studies that might appear to meet the inclusion criteria, but which were excluded, and explain why they were excluded.	Results: Study selection (p6-7); Figure 1. Flow diagram detailing results of searches and study selection process; Supplementary 2. Systematic review searches performed and results
Study characteristics	17	Cite each included study and present its characteristics.	Results: Description of included studies (p7-8)
Risk of bias in studies	18	Present assessments of risk of bias for each included study.	Results: Quality assessment (p7); Supplementary 2 Assessment of bias for case-control studies according to RoBANs; Supplementary 2 Assessment of bias of cohort studies using the Newcastle-Ottawa Quality Assessment form for cohort studies

For peer review only

Section and Topic	Item #	Checklist item	Location where item is reported
Results of individual studies	19	For all outcomes, present, for each study: (a) summary statistics for each group (where appropriate) and (b) an effect estimate and its precision (e.g. confidence/credible interval), ideally using structured tables or plots.	Table 1. Characteristics of included studies
Results of syntheses	20a	For each synthesis, briefly summarise the characteristics and risk of bias among contributing studies.	Table 1. Characteristics of included studies<br>Figure 3. Relationship between serum 25-hydroxyvitamin D and Benign Paroxysmal Positional Vertigo recurrence.
	20b	Present results of all statistical syntheses conducted. If meta-analysis was done, present for each the summary estimate and its precision (e.g. confidence/credible interval) and measures of statistical heterogeneity. If comparing groups, describe the direction of the effect.	Results: Meta analysis (p8-9)<br>Figure 3. Relationship between serum 25-hydroxyvitamin D and Benign Paroxysmal Positional Vertigo recurrence.
	20c	Present results of all investigations of possible causes of heterogeneity among study results.	Not applicable
	20d	Present results of all sensitivity analyses conducted to assess the robustness of the synthesized results.	Not applicable
Reporting biases	21	Present assessments of risk of bias due to missing results (arising from reporting biases) for each synthesis assessed.	Not applicable
Certainty of evidence	22	Present assessments of certainty (or confidence) in the body of evidence for each outcome assessed.	Not applicable
**DISCUSSION**			
Discussion	23a	Provide a general interpretation of the results in the context of other evidence.	Discussion (p11-12)
	23b	Discuss any limitations of the evidence included in the review.	Strengths and limitations of the study (p3); Discussion (p11-12)
	23c	Discuss any limitations of the review processes used.	Strengths and limitations (p3)
	23d	Discuss implications of the results for practice, policy, and future research.	Discussion (p11-12)
**OTHER INFORMATION**			Prospero registration number (p.2)
Registration and protocol	24a	Provide registration information for the review, including register name and registration number, or state that the review was not registered.	Abstract: Trial registration (p2)
	24b	Indicate where the review protocol can be accessed, or state that a protocol was not prepared.	Prospero registration number (p.2)
	24c	Describe and explain any amendments to information provided at registration or in the protocol.	Prospero registration number (p.2)
Support	25	Describe sources of financial or non-financial support for the review, and the role of the funders or sponsors in the review.	Funding sources (p14)

For peer review only

Section and Topic	Item #	Checklist item	Location where item is reported
Competing interests	26	Declare any competing interests of review authors.	Competing interests statement (p14)
Availability of data, code and other materials	27	Report which of the following are publicly available and where they can be found: template data collection forms; data extracted from included studies; data used for all analyses; analytic code; any other materials used in the review.	Methods: Synthesis methods (p6) and Supplementary 2 tables 2 and 3

From: Page MJ, McKenzie JE, Bossuyt PM, Boutron I, Hoffmann TC, Mulrow CD, et al. The PRISMA 2020 statement: an updated guideline for reporting systematic reviews. BMJ 2021;372:n71. doi: 10.1136/bmj.n71

For more information, visit: http://www.prisma-statement.org/

For peer review only

Supplementary 2, Table 5. PRISMA Abstract Checklist

Section and Topic	Item #	Checklist item	Reported (Yes/No)
**TITLE**			
Title	1	Identify the report as a systematic review.	YES
**BACKGROUND**			
Objectives	2	Provide an explicit statement of the main objective(s) or question(s) the review addresses.	YES
**METHODS**			
Eligibility criteria	3	Specify the inclusion and exclusion criteria for the review.	YES
Information sources	4	Specify the information sources (e.g. databases, registers) used to identify studies and the date when each was last searched.	YES
Risk of bias	5	Specify the methods used to assess risk of bias in the included studies.	YES
Synthesis of results	6	Specify the methods used to present and synthesise results.	YES
**RESULTS**			
Included studies	7	Give the total number of included studies and participants and summarise relevant characteristics of studies.	YES
Synthesis of results	8	Present results for main outcomes, preferably indicating the number of included studies and participants for each. If meta-analysis was done, report the summary estimate and confidence/credible interval. If comparing groups, indicate the direction of the effect (i.e. which group is favoured).	YES
**DISCUSSION**			
Limitations of evidence	9	Provide a brief summary of the limitations of the evidence included in the review (e.g. study risk of bias, inconsistency and imprecision).	YES
Interpretation	10	Provide a general interpretation of the results and important implications.	YES
**OTHER**			
Funding	11	Specify the primary source of funding for the review.	YES
Registration	12	Provide the register name and registration number.	YES

From: Page MJ, McKenzie JE, Bossuyt PM, Boutron I, Hoffmann TC, Mulrow CD, et al. The PRISMA 2020 statement: an updated guideline for reporting systematic reviews. BMJ 2021;372:n71. doi: 10.1136/bmj.n71

For more information, visit: http://www.prisma-statement.org/

For peer review only - http://bmjopen.bmj.com/site/about/guidelines.xhtml

PRISMA Checklist

Section and Topic	Item #	Checklist item	Location where item is reported
**TITLE**			
Title	1	Identify the report as a systematic review.	Title page (p1)
**ABSTRACT**			
Abstract	2	See the PRISMA 2020 for Abstracts checklist.	Abstract (p2)
**INTRODUCTION**			
Rationale	3	Describe the rationale for the review in the context of existing knowledge.	Introduction (p4)
Objectives	4	Provide an explicit statement of the objective(s) or question(s) the review addresses.	Introduction (p4)
**METHODS**			
Eligibility criteria	5	Specify the inclusion and exclusion criteria for the review and how studies were grouped for the syntheses.	Methods: Inclusion and Exclusion criteria (p5)
Information sources	6	Specify all databases, registers, websites, organisations, reference lists and other sources searched or consulted to identify studies. Specify the date when each source was last searched or consulted.	Methods: Data sources and search strategy (p5)
Search strategy	7	Present the full search strategies for all databases, registers and websites, including any filters and limits used.	Methods: Data sources and search strategy (p5)
Selection process	8	Specify the methods used to decide whether a study met the inclusion criteria of the review, including how many reviewers screened each record and each report retrieved, whether they worked independently, and if applicable, details of automation tools used in the process.	Methods: Inclusion and Exclusion criteria (p5)
Data collection process	9	Specify the methods used to collect data from reports, including how many reviewers collected data from each report, whether they worked independently, any processes for obtaining or confirming data from study investigators, and if applicable, details of automation tools used in the process.	Methods: Data Extraction (p6)
Data items	10a	List and define all outcomes for which data were sought. Specify whether all results that were compatible with each outcome domain in each study were sought (e.g. for all measures, time points, analyses), and if not, the methods used to decide which results to collect.	Methods: Data Extraction (p6)
	10b	List and define all other variables for which data were sought (e.g. participant and intervention characteristics, funding sources). Describe any assumptions made about any missing or unclear information.	Methods: Data Extraction (p6)
Study risk of bias assessment	11	Specify the methods used to assess risk of bias in the included studies, including details of the tool(s) used, how many reviewers assessed each study and whether they worked independently, and if applicable, details of automation tools used in the process.	Methods: Assessment of bias (p6)
Effect measures	12	Specify for each outcome the effect measure(s) (e.g. risk ratio, mean difference) used in the synthesis or presentation of results.	Methods: Synthesis methods (p6)
Synthesis methods	13a	Describe the processes used to decide which studies were eligible for each synthesis (e.g. tabulating the study intervention characteristics and comparing against the planned groups for each synthesis (item #5)).	Methods: Synthesis methods (p6)
	13b	Describe any methods required to prepare the data for presentation or synthesis, such as handling of missing	Methods: Synthesis

For peer review only

Section and Topic	Item #	Checklist item	Location where item is reported
		summary statistics, or data conversions.	methods (p6)
	13c	Describe any methods used to tabulate or visually display results of individual studies and syntheses.	Methods: Synthesis methods (p6)
	13d	Describe any methods used to synthesize results and provide a rationale for the choice(s). If meta-analysis was performed, describe the model(s), method(s) to identify the presence and extent of statistical heterogeneity, and software package(s) used.	Methods: Synthesis methods (p6)
	13e	Describe any methods used to explore possible causes of heterogeneity among study results (e.g. subgroup analysis, meta-regression).	Not applicable
	13f	Describe any sensitivity analyses conducted to assess robustness of the synthesized results.	Not applicable
Reporting bias assessment	14	Describe any methods used to assess risk of bias due to missing results in a synthesis (arising from reporting biases).	Not applicable
Certainty assessment	15	Describe any methods used to assess certainty (or confidence) in the body of evidence for an outcome.	Not applicable
**RESULTS**			
Study selection	16a	Describe the results of the search and selection process, from the number of records identified in the search to the number of studies included in the review, ideally using a flow diagram.	Results: Study selection (p6-7); Figure 1. Flow diagram detailing results of searches and study selection process; Supplemental 2. Systematic review searches performed and results
	16b	Cite studies that might appear to meet the inclusion criteria, but which were excluded, and explain why they were excluded.	Results: Study selection (p6-7); Figure 1. Flow diagram detailing results of searches and study selection process; Supplemental 2. Systematic review searches performed and results
Study characteristics	17	Cite each included study and present its characteristics.	Results: Description of included studies (p7-8)
Risk of bias in studies	18	Present assessments of risk of bias for each included study.	Results: Quality assessment (p7); Supplemental 2 Assessment of bias for case-control studies

For peer review only

Section and Topic	Item #	Checklist item	Location where item is reported
			according to RoBANs; Supplemental 2 Assessment of bias of cohort studies using the Newcastle-Ottawa Quality Assessment form for cohort studies
Results of individual studies	19	For all outcomes, present, for each study: (a) summary statistics for each group (where appropriate) and (b) an effect estimate and its precision (e.g. confidence/credible interval), ideally using structured tables or plots.	Table 1. Characteristics of included studies
Results of syntheses	20a	For each synthesis, briefly summarise the characteristics and risk of bias among contributing studies.	Table 1. Characteristics of included studies<br>Figure 3. Relationship between serum 25-hydroxyvitamin D and Benign Paroxysmal Positional Vertigo recurrence.
	20b	Present results of all statistical syntheses conducted. If meta-analysis was done, present for each the summary estimate and its precision (e.g. confidence/credible interval) and measures of statistical heterogeneity. If comparing groups, describe the direction of the effect.	Results: Meta analysis (p8-9)<br>Figure 3. Relationship between serum 25-hydroxyvitamin D and Benign Paroxysmal Positional Vertigo recurrence.
	20c	Present results of all investigations of possible causes of heterogeneity among study results.	Not applicable
	20d	Present results of all sensitivity analyses conducted to assess the robustness of the synthesized results.	Not applicable
Reporting biases	21	Present assessments of risk of bias due to missing results (arising from reporting biases) for each synthesis assessed.	Not applicable
Certainty of evidence	22	Present assessments of certainty (or confidence) in the body of evidence for each outcome assessed.	Not applicable
**DISCUSSION**			
Discussion	23a	Provide a general interpretation of the results in the context of other evidence.	Discussion (p11-12)
	23b	Discuss any limitations of the evidence included in the review.	Strengths and limitations of the study (p3); Discussion (p11-12)

For peer review only

Section and Topic	Item #	Checklist item	Location where item is reported
	23c	Discuss any limitations of the review processes used.	Strengths and limitations (p3)
	23d	Discuss implications of the results for practice, policy, and future research.	Discussion (p11-12)
**OTHER INFORMATION**			Prospero registration number (p.2)
Registration and protocol	24a	Provide registration information for the review, including register name and registration number, or state that the review was not registered.	Abstract: Trial registration (p2)
	24b	Indicate where the review protocol can be accessed, or state that a protocol was not prepared.	Prospero registration number (p.2)
	24c	Describe and explain any amendments to information provided at registration or in the protocol.	Prospero registration number (p.2)
Support	25	Describe sources of financial or non-financial support for the review, and the role of the funders or sponsors in the review.	Funding sources (p14)
Competing interests	26	Declare any competing interests of review authors.	Competing interests statement (p14)
Availability of data, code and other materials	27	Report which of the following are publicly available and where they can be found: template data collection forms; data extracted from included studies; data used for all analyses; analytic code; any other materials used in the review.	Methods: Synthesis methods (p6) and Supplemental 2 tables 2 and 3

From: Page MJ, McKenzie JE, Bossuyt PM, Boutron I, Hoffmann TC, Mulrow CD, et al. The PRISMA 2020 statement: an updated guideline for reporting systematic reviews. BMJ 2021;372:n71. doi: 10.1136/bmj.n71

For more information, visit: http://www.prisma-statement.org/

For peer review only

PRISMA Abstract Checklist

Section and Topic	Item #	Checklist item	Reported (Yes/No)
**TITLE**			
Title	1	Identify the report as a systematic review.	YES
**BACKGROUND**			
Objectives	2	Provide an explicit statement of the main objective(s) or question(s) the review addresses.	YES
**METHODS**			
Eligibility criteria	3	Specify the inclusion and exclusion criteria for the review.	YES
Information sources	4	Specify the information sources (e.g. databases, registers) used to identify studies and the date when each was last searched.	YES
Risk of bias	5	Specify the methods used to assess risk of bias in the included studies.	YES
Synthesis of results	6	Specify the methods used to present and synthesise results.	YES
**RESULTS**			
Included studies	7	Give the total number of included studies and participants and summarise relevant characteristics of studies.	YES
Synthesis of results	8	Present results for main outcomes, preferably indicating the number of included studies and participants for each. If meta-analysis was done, report the summary estimate and confidence/credible interval. If comparing groups, indicate the direction of the effect (i.e. which group is favoured).	YES
**DISCUSSION**			
Limitations of evidence	9	Provide a brief summary of the limitations of the evidence included in the review (e.g. study risk of bias, inconsistency and imprecision).	YES
Interpretation	10	Provide a general interpretation of the results and important implications.	YES
**OTHER**			
Funding	11	Specify the primary source of funding for the review.	YES
Registration	12	Provide the register name and registration number.	YES

From: Page MJ, McKenzie JE, Bossuyt PM, Boutron I, Hoffmann TC, Mulrow CD, et al. The PRISMA 2020 statement: an updated guideline for reporting systematic reviews. BMJ 2021;372:n71. doi: 10.1136/bmj.n71

For more information, visit: http://www.prisma-statement.org/

For peer review only

BMJ Open

Association between vitamin D deficiency and Benign Paroxysmal Positional Vertigo (BPPV) incidence and recurrence: A systematic review and meta-analysis

Journal:	*BMJ Open*
Manuscript ID	bmjopen-2023-077986.R2
Article Type:	Original research
Date Submitted by the Author:	24-Feb-2024
Complete List of Authors:	Wood, Heather; University of Birmingham, College of Medical and Dental Sciences<br>Kluk, Karolina; The University of Manchester, Manchester Centre for Audiology and Deafness<br>Binkhamis, Ghada; The University of Manchester, Manchester Centre for Audiology and Deafness (ManCAD) ; King Fahad Medical City, Communication and Swallowing Disorders
**Primary Subject Heading**:	Ear, nose and throat/otolaryngology
Secondary Subject Heading:	General practice / Family practice
Keywords:	Audiology < OTOLARYNGOLOGY, Adult otolaryngology < OTOLARYNGOLOGY, Systematic Review, Meta-Analysis

SCHOLARONE™
Manuscripts

I, the Submitting Author has the right to grant and does grant on behalf of all authors of the Work (as defined in the below author licence), an exclusive licence and/or a non-exclusive licence for contributions from authors who are: i) UK Crown employees; ii) where BMJ has agreed a CC-BY licence shall apply, and/or iii) in accordance with the terms applicable for US Federal Government officers or employees acting as part of their official duties; on a worldwide, perpetual, irrevocable, royalty-free basis to BMJ Publishing Group Ltd ("BMJ") its licensees and where the relevant Journal is co-owned by BMJ to the co-owners of the Journal, to publish the Work in this journal and any other BMJ products and to exploit all rights, as set out in our licence.

The Submitting Author accepts and understands that any supply made under these terms is made by BMJ to the Submitting Author unless you are acting as an employee on behalf of your employer or a postgraduate student of an affiliated institution which is paying any applicable article publishing charge ("APC") for Open Access articles. Where the Submitting Author wishes to make the Work available on an Open Access basis (and intends to pay the relevant APC), the terms of reuse of such Open Access shall be governed by a Creative Commons licence – details of these licences and which Creative Commons licence will apply to this Work are set out in our licence referred to above.

Other than as permitted in any relevant BMJ Author's Self Archiving Policies, I confirm this Work has not been accepted for publication elsewhere, is not being considered for publication elsewhere and does not duplicate material already published. I confirm all authors consent to publication of this Work and authorise the granting of this licence.

For peer review only

Association between vitamin D deficiency and Benign Paroxysmal Positional Vertigo (BPPV) incidence and recurrence: A systematic review and meta-analysis

Heather Wood[1], Karolina Kluk[2], Ghada BinKhamis[2,3]

[1]*College of Medical and Dental Sciences, University of Birmingham, United Kingdom;* [2]*Manchester Centre for Audiology and Deafness, Manchester Academic Health Science Centre, University of Manchester, Manchester, UK;* [3]*Department of Communication and Swallowing Disorders, King Fahad Medical City, Riyadh, Saudi Arabia*

Address for correspondence:

Ghada BinKhamis: ghada.binkhamis@manchester.ac.uk / gbinkhamis@gmail.com

Karolina Kluk: Karolina.Kluk@manchester.ac.uk

Heather Wood: hmw998@student.bham.ac.uk

Manchester Centre for Audiology and Deafness (ManCAD), School of Health Sciences, Faculty of Biology Medicine and Health, Room B2.15, Ellen Wilkinson Building, University of Manchester, Oxford Road, Manchester, M13 9PL, United Kingdom.

ORCiD:

- Ghada BinKhamis: 0000-0002-7696-1073
- Karolina Kluk: 0000-0003-3638-2787

Word Count: 3956

For peer review only - http://bmjopen.bmj.com/site/about/guidelines.xhtml

Abstract

OBJECTIVES: To determine the relationship between serum vitamin D level and the risk of developing benign paroxysmal positional vertigo (BPPV) incidence and recurrence in countries in the Northern hemisphere.

DESIGN: Systematic review and meta-analysis.

DATA SOURCES: PUBMED, SCOPUS and Web of Science databases were searched for studies published between January 2000 and February 2023.

ELIGIBILITY CRITERIA FOR SELECTING STUDIES: Participants located in the Northern hemisphere aged 18 or over with at least one episode of BPPV, serum 25-hydroxyvitamin D levels measured and reported, no comorbidities or history of vitamin D supplementation.

DATA EXTRACTION AND SYNTHESIS: Were performed by a single reviewer and checked by a second reviewer. Inclusion and exclusion criteria and risk of bias were assessed by 2 independent reviewers using the Newcastle Ottawa Tool for Cohort studies and RoBANS checklist for case-control studies. Meta-analysis was conducted using random effects models. Standard mean difference with a 95% confidence interval was used to measure the relationship between vitamin D level and BPPV.

RESULTS: The 35 articles identified by the literature search reported data of 9843 individuals. 19 studies (7387 individuals) were included in the BPPV incidence meta-analysis whilst seven studies (622 individuals) were included in the BPPV recurrence meta-analysis. Lower serum vitamin D levels were found in BPPV incidence compared to controls, but the relationship between vitamin D levels in recurrent BPPV compared to non-recurrent disease remained uncertain.

CONCLUSION: Results of this systematic review and meta-analysis demonstrated a negative correlation between serum vitamin D and BPPV incidence, whilst any relationship between serum vitamin D and BPPV recurrence remained uncertain. Risk of bias analysis revealed evidence of variable quality. There was insufficient data available to evaluate seasonal relationships between serum vitamin D and BPPV. Given the potential for this as a confounding factor, future research should aim to investigate this further.

PROSPERO REGISTRATION: CRD42021271840

For peer review only - http://bmjopen.bmj.com/site/about/guidelines.xhtml

Strengths and limitations of the study

- Strengths:
    - This is a comprehensive systematic review of vitamin D and Benign Paroxysmal Positional Vertigo (BPPV) covering literature published between January 2000 and February 2023
    - The meta-analyses include a large number of individuals (7510)
- Limitations:
    - It was not possible to account for the impact of seasonality on both serum vitamin D levels and incidence and recurrence rates of BPPV as the majority of studies did not report the dates or months when patients were seen.
    - Data were not available relating to factors potentially related to both BPPV and vitamin D such as subject occupation, comorbidities and symptom severity.
    - High-quality research is currently limited, and a few of the identified studies in this systematic review were from journals with unclear peer-review processes or non-impact journals.

Keywords:

BPPV, Benign Paroxysmal Positional Vertigo, Vitamin D, Vitamin D Deficiency

For peer review only

Introduction

The impact of vitamin D on audiovestibular health has been a research area of interest in recent years, with low serum levels of vitamin D associated with otological pathologies including benign paroxysmal positional vertigo (BPPV).[1] Due to vitamin D's role in skeletal mineral homeostasis, it has been suggested that audiovestibular pathology may be related to high bone turnover rates in the temporal bone and/or at a systemic level.[2] Another hypothesis suggests that inner ear pathology including BPPV may be secondary to an inflammatory or autoimmune response following viral infection, and that the immunomodulatory functions of vitamin D may be protective against this secondary response.[3]

There are two major bioavailable forms of vitamin D; vitamin D2 (also known as ergocalciferol) and vitamin D3 (cholecalciferol). Ergocalciferol can be ingested via diet or supplementation, whilst vitamin D3 is largely synthesised in human skin following exposure to UVB light (and may also be ingested via dietary supplements or animal-based foods).[4] The bioactive form of vitamin D3 is known to play an important role in calcium homeostasis and bone health; increasing the absorption of dietary calcium and phosphorus which in turn affects the regulation of bone mineral density.[5] Vitamin D3 is hydroxylated in the liver into 25-hydroxyvitamin D (calcifediol), before being processed further by the kidneys, converting it to its bioactive form (calcitriol).[5] Levels of the calcitriol precursor 25-hydroxyvitamin D (calcidiol) can be measured from blood samples and used to indicate the overall abundance of vitamin D within the body.

BPPV is a common vestibular disorder, characterised by positional vertigo and nystagmus elicited by head movements. It is caused by the displacement of small crystals of calcium carbonate known as otoconia into one of the semi-circular canals (most commonly a posterior canal). Displacement of otoconia can occur following head trauma, however most cases are idiopathic and the exact pathophysiology of BPPV currently not known.[6]

The relationship between vitamin D and BPPV is currently a popular topic of research, and a number of systematic reviews have been published in recent years indicating that BPPV incidence is associated with vitamin D deficiency,[7, 8] although this relationship was not supported by an earlier review.[9] There is mixed evidence regarding the relationship between vitamin D levels and BPPV recurrence, with some reviews finding lower levels of the vitamin in patients with recurrent BPPV,[9, 10] whilst others have observed no significant difference in vitamin D levels and BPPV recurrence.[11] Supplementation in BPPV patients with low vitamin D levels has also been shown to result in prolonged improvement in symptoms and fewer episodes of recurrent BPPV.[12, 13]

There is currently uncertainty relating to how seasonal patterns of vitamin D and BPPV incidence are related. In countries within the northern hemisphere, vitamin D levels fluctuate in a seasonal pattern- serum vitamin D levels are highest within the population of these countries during summer months and lowest during winter and spring, when there is a high prevalence of vitamin deficiency.[14, 15] BPPV may also exhibit a seasonal pattern, with higher incidence rates seen between autumn and early spring,[16 – 18] although this has not been replicated in every study.[19, 20]

This systematic review will examine the evidence concerning the relationship between serum vitamin D and BPPV for patients in northern hemisphere countries, the first time to the authors' knowledge that a review on this topic has been restricted by geographic location.

For peer review only - http://bmjopen.bmj.com/site/about/guidelines.xhtml

Methods

This systematic review was conducted following the methodology of Higgins *et al.*[21] as closely as possible, and was reported following the Preferred Reporting Items for Systematic reviews and Meta-Analyses (PRISMA),[22] and was pre-registered in **PROSPERO (**CRD42021271840, see Supplementary 1) Ethics approval was not necessary for this study as no new data were collected and no patient-identifiable data included.

Patient and public involvement. No patient involved.

Data sources and search strategy

A systematic literature search was performed in PUBMED, SCOPUS and Web of Science databases relating to the association between vitamin D and benign paroxysmal positional vertigo (BPPV). Unpublished studies were not sought. Date was restricted to include studies published in English between 2000 and 2023. Three searches were run through each database to account for differing possible terms for BPPV, making up a total of 12 searches which were completed on the 18th of February 2023. Search criteria and results are described in Supplementary 2, Table 1. Microsoft Excel software (Microsoft Office for Windows, version 2204) was used to store and process search results.

Studies published before the year 2000 and those pertaining to animal research were removed, then any entries that did not contain keywords "BPPV", "Vertigo", "Vitamin D" or "25-hydroxyvitamin D" within their title or abstract were excluded. Entries that were not original research or did not include multiple participants (e.g., case reports, letters and editorials) were also removed, leaving a total of 68 full texts to be manually reviewed. A manual search of the reference list of included full texts and systematic reviews identified by the search strategy was also performed to identify any further relevant literature. The included articles were reviewed independently by two reviewers for inclusion evaluation. Any inconsistencies between the two reviewers were evaluated further by both reviewers until a consensus was reached.

Inclusion and Exclusion criteria

To better account for seasonal differences in serum vitamin D levels, the geographical location of study participants was limited to the Northern hemisphere. All studies that were available with English full texts that investigated the association between serum 25-hydroxyvitamin D levels in adult patients diagnosed with at least one episode of benign paroxysmal positional vertigo (BPPV) were included. Studies were excluded if participants had past medical history of ENT comorbidities, head trauma or were taking vitamin D supplements at the start of the study.

The literature search was undertaken by HW. Study selection was independently undertaken by HW and GB, with inconsistencies resolved through discussion between the reviewers. Studies were deemed eligible for inclusion if they met the following criteria: 1) participants were over the age of 18 with at least one episode of diagnosed BPPV 2) participants resided in countries in the Northern hemisphere; 3) serum 25-hydroxyvitamin D levels were measured and reported; 4) participants had no past medical history of head trauma or ENT comorbidities.

Articles were excluded according to the following criteria: 1) Study did not contain reference to "BPPV", "vertigo" or "vitamin D" in title or abstract; 2) participants were taking vitamin D supplements prior to commencement of the study; 3) The article did not pertain to a research study (reviews, editorials, case studies). During application of inclusion and exclusion criteria, the decision was taken to exclude articles which included only participants with BPPV and pre-existing vitamin D deficiency as

For peer review only - http://bmjopen.bmj.com/site/about/guidelines.xhtml

these would not be able to be utilised in subsequent analysis of the relationship between BPPV disease state and serum vitamin levels.

Assessment of bias

Microsoft Excel and Microsoft Word software (Microsoft Office for Windows, version 2204) was used by the reviewers to record assessment of bias and document comments.

Assessment of bias was performed by two independent reviewers and any inconsistencies resolved by discussion between the reviewers. The case-control studies were assessed using the Risk of Bias Assessment Tool for Nonrandomized Studies (RoBANS)[23] which examines selection of participants, confounding variables, intervention measurement, blinding of outcome assessment, intervention measurement, incomplete outcome data and selective outcome reporting. The cohort studies were assessed according to the Newcastle Ottawa Scale (NOS),[24] as recommended by the Cochrane Collaboration.[21] The NOS examines participant selection, comparability and outcome and assigns studies as being of 'poor', 'fair' or 'good' quality.

Data Extraction

Data extraction was completed by HW and double checked by GB. Extracted data included: study design, author, year published, location of study participants, mean age of participants, range of participant age, sample size, percentage male participants, serum vitamin D levels for participants and the season study was performed (where reported). Standardised mean difference (SMD) with a 95% confidence interval was used to calculate the level of vitamin D in case and control groups. Based on recommendations from the clinical practice guidelines of the Endocrine Society Task Force on Vitamin D, plasma vitamin D levels of less than 20ng/ml were considered to indicate vitamin deficiency.[25] Microsoft Excel software (Microsoft Office for Windows, version 2204) was used to tabulate the extracted data.

Synthesis methods

Studies were included if serum 25-hydroxyvitamin D levels (mean and standard deviation; ng/ml) were reported for cases with BPPV and controls with no medical history of BPPV. Where vitamin D levels were reported for subgroups within a study (for example male and female means reported separately), a population mean and standard deviation for vitamin D levels for both cases and controls was calculated according to the recommendations of the Cochrane Collaboration.[21]

The I^2 statistic was used to examine heterogeneity between studies, with I^2 >40% regarded as representing significant heterogeneity. The random-effects model was to be employed for the meta-analysis if I^2 >40%, whilst the fixed-effect model was to be used instead if I^2 <40%. Statistical analysis was completed and forest plots generated using Review Manager (RevMan) software version 5.4.1. (64 bit; The Cochrane Collaboration, 2020).

Results

Study selection

The initial searches identified 1113 results comprising 350 individual articles (**Figure 1**). 282 of these were excluded; 163 articles were excluded because they did not contain reference to "BPPV" or "vertigo" within their title or abstract, 61 were removed as they were not peer reviewed or based on original research (review articles, conference posters, editorials etc.), 27 did not contain "vitamin D"

For peer review only - http://bmjopen.bmj.com/site/about/guidelines.xhtml

or related terms in their title or abstract, 6 were excluded as they related to animal studies and 25 studies were removed as they were erroneously included in search results despite being published before the year 2000.

The full text of the remaining 68 articles was reviewed for inclusion evaluation; eight articles were excluded as the participants were receiving vitamin D supplementation before commencement of the study; two were excluded as they included patients with past medical history of head trauma or ENT-related comorbidities; five were excluded as serum vitamin D levels were not measured or reported; two were excluded as the patients resided in countries in the Southern hemisphere; seven because they included participants under the age of 18 and a further five were excluded as they described patients with causes of vertigo other than BPPV. Four articles were also excluded as they included only participants with both BPPV and pre-existing vitamin D deficiency, precluding analysis of the relationship between disease state and serum vitamin levels. A manual search of the reference lists of these studies and identified systematic reviews did not yield any additional articles to include in the review. A total of 35 articles underwent full-length review.

Quality assessment

The 26 articles assessed with the RoBANS tool are described in Supplementary 2, Figure 1. All the studies were found to have a low risk of bias in three of the six domains assessed ("intervention measurement", "incomplete outcome data" and "selective outcome reporting" domains). 14 of the studies had an uncertain risk of bias in either or both of the "selection of participant" and "confounding variables" domains. All but two of the studies had a high risk of bias in the domain relating to "blinding of outcome assessment", four had a high risk of bias relating to "confounding variables" and one article had a high risk of bias in the "selection of participants" domain. None of the articles was judged to have a low risk of bias in all six domains.

The nine cohort studies assessed using the Newcastle Ottawa scale (NoS) ranged in quality from 'poor' to 'good' and are described in Supplementary 2, Figure 2. There were significant limitations using the NoS for some of the studies, meaning that one of the eight domains of the tool ("adequacy of follow-up of cohorts") was not applicable for five of the nine studies assessed by this tool. One study was determined to be of 'poor' quality, two 'fair' quality and the remaining six studies were 'good' quality. High risk of bias was most commonly seen in the "outcome of interest not present at start domain" and was present in seven out of nine studies. High risk of bias was seen in two studies in the "representativeness of exposed cohort" domain and in one study for the "follow-up long enough" domain. A single study was found to have a low risk of bias in all eight domains.

Description of included studies

Table 1 describes the characteristics of the included studies investigating the association between serum vitamin D and BPPV for patients within northern hemisphere countries. The 35 studies included 9,843 participants in total: 26 were case-control studies and 9 were cohort studies; there were no randomised controlled trials. Sample size varied from 10 to 3505 individuals, sex distribution from 0 to 100% male and age ranged between 18 to 89 years. Most studies were conducted in countries situated in Asia (twelve in China, six in Turkey, four in the Republic of Korea, two in India, and one each in Japan, Kuwait and Thailand); six studies in Europe (three in Italy and one each in Croatia, Germany and Portugal); one study was conducted in Africa (Egypt) and one in North America (USA).

A total of 12 studies reported that compared with controls, patients with BPPV have lower average serum vitamin D levels.[1, 26 – 35] Seven studies found no significant difference between the serum vitamin D levels of BPPV patients and controls.[36 – 42] Amongst cases and controls with osteoporosis

For peer review only - http://bmjopen.bmj.com/site/about/guidelines.xhtml

For peer review only

and osteopenia, two studies[2, 43] found no significant difference between cases and controls, although osteoporosis was correlated with low serum vitamin D in BPPV patients.[2] Serum vitamin D levels were also reported to be lower in recovering BPPV patients with residual dizziness, compared to those without.[44]

The relationship between BPPV recurrence and serum 25-hydroxyvitamin D was examined in 12 studies, with nine studies reporting lower serum vitamin D levels in patients with recurrent BPPV compared to those experiencing an initial attack[1, 31, 35 – 37, 42, 45, 46] and three reporting no significant difference in vitamin D levels between the two groups.[38, 47, 48] Three studies[41, 49, 50] reported that vitamin D supplementation reduced the number of recurrent episodes of BPPV. Vitamin D supplementation was seen to improve the bone mineral density of BPPV patients in one study, however the effects on BPPV recurrence were not reported.[51]

Whether vitamin D levels differed between sub-types of BPPV was assessed in four studies, two of which found that serum 25-hydroxyvitamin D were lower in canalithiasis compared to cupulolithiasis[47, 52] whilst the remaining two found no significant difference in vitamin levels between BPPV sub-types.[29, 53] One study reported a much higher prevalence of vitamin D deficiency (<20ng/ml) in patients undergoing an acute attack of BPPV compared with 6 months post-episode (93.5% and 38.7% respectively).[54]

Seasonality and BPPV was examined by two included studies; Shu and colleagues[16] reported that the median number of BPPV referrals was higher in winter compared to summer months, whilst Califano *et al.*[36] found no seasonal patterns of BPPV cases and high levels of vitamin D deficiency and insufficiency in both winter and summer.

Meta-analysis

A total of 19 studies reported mean and standard deviation for serum 25-hydroxyvitamin D levels in case (BPPV) and control (non-BPPV) groups and were included in the meta-analysis (Supplementary 2, Table 2). These described a total of 7387 participants (2325 cases; 5062 controls), ranging in age from 18 to 88 years and sex distribution 0 to 100% male. Seven studies were excluded for the following reasons: four because the case and control groups both had BPPV,[44, 46, 49, 51] two as standard deviation was not reported[28, 41] and one as vitamin D levels were not given in ng/mL.[43] Combined mean averages and standard deviations were calculated for cases and controls using data provided in three studies[1, 35, 55] as these were initially reported according to groups (age, sex, BPPV recurrence).

Standard deviations were recalculated for one study[42] after consultation with a statistician as reported standard deviations were not congruent with reported means or confidence intervals. All included studies had 'uncertain' or 'high' risk of bias in at least one domain according to RoBANs, whilst two[36, 37] were high risk in two domains. These were not excluded as the results of both were in accordance with the rest of the studies, and neither the trend or significance of the analysis was affected on removal of one or both studies from the analysis. Pooled standardised mean difference (SMD) indicated that serum vitamin D was on average lower in cases with BPPV compared to controls (SMD -2.07; 95% confidence interval -3.32, -0.81; $p = 0.001$ random effects model; **Figure 2**). There was substantial heterogeneity across included studies ($I^2 = 92\%$; $P < 0.00001$).

Seven studies reported mean and standard deviation for serum 25-hydroxyvitamin D levels in patient groups with recurrent and non-recurrent BPPV and were included in the meta-analysis (Supplementary 2, Table 3). These described a total of 622 participants (255 cases; 367 controls), ranging in age from 20 to 89 years and sex distribution from 0 to 35% male. Eight studies were excluded

For peer review only - http://bmjopen.bmj.com/site/about/guidelines.xhtml

from the meta-analysis for the following reasons: three because no standard deviation was reported,[28, 47, 56] three because serum vitamin D levels were not reported and two because vitamin D supplements were utilised.[36, 49] All included studies were of 'Good' quality according to the NoS or had 'uncertain' or 'high' risk of bias in at least one domain according to RoBANs. Pooled standardised mean difference (SMD) indicated that serum vitamin D is no different in individuals with recurrent BPPV compared to those with non-recurrent disease (SMD -1.90; 95% confidence interval -3.80, 0.00; p = 0.05 random effects model; **Figure 3**). There was moderate heterogeneity across included studies (I^2 = 66%; P = 0.007).

For peer review only

***Table 1*. Characteristics of included studies.** NOS = Newcastle Ottawa Scale (Wells et al 2018).

Author and year	Sample size	Study design	Country	Age range	Case Mean age (SD)	Case % male	Case 25(OH)D (ng/ml) mean (SD)	Quality
Bi *et al.* 2021	52 (27 case, 25 control)	Prospective Case-control	China	not reported	51.6 ± 15.8	33.33	14.64 ± 6.94*	n/a RoBANS
Califano *et al.* 2019	227 (127 case, 100 control)	Retro- + Pro-spective Case-control	Italy	not reported	60 ± 11.5**	40.2	24.4 ± 10.69	n/a RoBANS
Carneiro de Sousa *et al.* 2019	10 (5 case, 5 control)	Prospective Case-control	Portugal	52-82	64 ± 9.3 (case + control)	0	13.6 ± 5.7**	n/a RoBANS
Çelik *et al.* 2021	339 (190 case, 149 control)	Prospective Case-control	Turkey	not reported	43.7 ± 16	57.4	15.64 ± 8.4*	n/a RoBANS
Ceylan and Kanmaz 2020	197 (97 case, 100 control)	Retrospective Case-control	Turkey	not reported	57.07 ± 12.6	34	15.87 ± 8.6*	n/a RoBANS
Cheng et al. 2021	640 (320 case, 320 control	Prospective Case-control	China	not reported	68.2±6.02	45	23.2±4.09	n/a RoBANS
Çıkrıkçı Işık *et al.* 2017	127 (64 case, 63 control)	Retrospective Case-control	Turkey	not reported	56 ± 13.1	26.6	9.51 ± 5.49*	n/a RoBANS
Ding *et al.* 2019	522 (174 case, 348 control)	Prospective Case-control	China	not reported	Median reported	41.4	18.2 (n/a)*	n/a RoBANS
Elmoursey and Abbas 2021	60	Prospective Case-control	Egypt	24-70	46 ± 12.6	40	26.1 ± 11.7	n/a RoBANS
Goldschagg *et al.* 2021	459 (158 cases, 301 control)	Retrospective Case-control	Germany	not reported	61 ± 14	44.9	23.4 ± 9.4	n/a RoBANS
Gu, Dong and Gu 2018	100 (50 case, 50 control)	Prospective Case-control	China	not reported	51.36 ± 10.58	40	17.62 ± 9.0	n/a RoBANS
Han *et al.* 2018	165 (85 case, 80 control)	Retrospective Case-control	China	44-88	63.5 ± 9.72	0	19.1 ± 5.2	n/a RoBANS
Han *et al.* 2020	117	Retrospective Cohort	Republic of Korea	25-78	55 ± 11	22.2	24.47 ± 11.6**	Good
Han *et al.* 2021	201	Retrospective Cohort	China	not reported	65.14 ± 13.35**	0	19.74 ± 6.4**	Fair
Inan *et al.* 2021	104 (52 case, 52 control)	Retrospective Case-control	Turkey	18-80	55.6 (n/a)	44	15.3 ± 9.8	n/a RoBANS
Kahraman *et al.* 2016	37	Retrospective Cohort	Turkey	23–75	49.8 (n/a)	35.5	9.73 ± 8.8 (initial visit)*	Good
Karataş et al 2017	156 (78 case, 78 control)	Retrospective Case-control	Turkey	22-85	51.4 ± 12.2	37.2	23.0 ± 14.4	n/a RoBANS
Lee *et al.* 2017	132	Retrospective Cohort	Republic of Korea	49-81	63 ± 10.0	0	29.43 ± 14.2**	Fair
Maslovara *et al.* 2018	40	Prospective Cohort	Croatia	not reported	64.2 ± 12.1	28	21 ± 7.4	Good
Melis *et al.* 2020	73	Prospective Cohort	Italy	not reported	62.99 ± 14.6**	27.4**	22.13 ± 15.1**	Good
Nakada *et al.* 2019	35	Retrospective Cohort	Japan	not reported	76.36 ± 6.4**	34.3**	16.29 ± 3.9**	Poor
Parham *et al.* 2013	29 (16 case, 13 control)	Prospective Case-Control	USA	49-81	68.5 (n/a)	0	reported as nM/L	n/a RoBANS
Pecci *et al.* 2022	50 (26 case, 24 control)	Prospective Case-control	Italy	39-79	61.85 (n/a)	23	20.18 (SD not given)	n/a RoBANS
Sarsitthithim *et al.* 2021	137 (69 case, 68 control)	Prospective Case-Control	Thailand	39-89	61.4 ± 11.5	14.5	21.5 ± 5.3	n/a RoBANS
Sen, Padiyar and Arora 2018	200 (100 case, 100 control)	Prospective Case-control	India	18-60	48.6 ± 10.2	33	20.3 ± 12.2	n/a RoBANS
Shu *et al.* 2019	877	Retrospective Cohort	China	not reported	Median reported	not reported	18.03 (n/a)**	n/a RoBANS
Shin *et al.* 2023	50	Retrospective Cohort	Republic of Korea	not reported	50.5 ± 14.6**	28**	Reported by group	Good
Song *et al.* 2020	3505 (380 case, 3125 control)	Prospective Case-Control	China	19-85	50.7 ± 13.6**	25.5	14.24 ± 6.6**	Good
Talaat *et al.* 2015	180 (80 case, 100 control)	Prospective Case-Control	Kuwait	31-71	47.6 ± 9.1	35	14.19 ± 9.3**	n/a RoBANS
Thomas *et al.* 2021	98 (49 cases, 49 controls)	Prospective Case-control	India	21-71	44.39 (n/a)	32.7	21.3 ± 9.567***	n/a RoBANS
Wang *et al.* 2020	183 (103 case, 80 control)	Prospective Case-Control	China	50-86	63.0 ± 9.3	0	17.2 ± 2.0	n/a RoBANS
Wu *et al.* 2018	152 (60 case, 92 control)	Retrospective Case-control	China	not reported	59.35 ± 13.24	100	23.0 ± 6.8	n/a RoBANS
Wu *et al.* 2022	123 (51 case; 72 control)	Prospective Case-control	China	not reported	58.5 ± 12.6**	32.5	Median reported	n/a RoBANS
Yang *et al.* 2017	260 (130 case, 130 control)	Retrospective Case-control	Republic of Korea	20-87	54.9 ± 12.2	23.1	18.2 ± 10.3	n/a RoBANS
Zhang *et al.* 2022	206 (156 case, 50 control)	Prospective Case-control	China	46-75	59.5 ± 7.4	0	18.8 ± 2.5**	n/a RoBANS

Abbreviations: 25(OH)D = 25hydroxyvitamin D, BPPV= benign paroxysmal positional vertigo, SD= standard deviation, *= vitamin deficiency (≤20 ng/ml)
** Calculated combined average/SD. Combines groups of means and Standard Deviations into a Single Group by Repeating Cochrane's formula; *** SD recalculated using reported mean and confidence intervals

For peer review only - http://bmjopen.bmj.com/site/about/guidelines.xhtml

Discussion

Our systematic review and meta-analyses were based on a total of 35 articles; 26 case-control studies and nine cohort studies. It provides up-to-date information regarding the evidence base for vitamin D and benign paroxysmal positional vertigo (BPPV). The included studies described 9843 participants, of which 7510 were included in the meta-analyses. The studies included a broad range of populations from thirteen different countries in the Northern Hemisphere.

Study quality was variable, and the majority of included studies were identified as having a high risk of bias in at least one domain after application of the NOS or ROBANS tools, most commonly due to a lack of blinding of outcome assessment. Some of the identified studies in this systematic review were also from journals with unclear peer-review processes or non-impact journals. Bias and potential bias was also identified relating to participant selection and unknown confounding variables, for example participant occupation, unmatched case and control selection and incomplete data relating to relevant comorbidities including renal failure. Whilst it was not possible to complete meta-analysis using only high-quality studies, the authors are reassured that the findings from this review are comparable to previously published literature,[7, 8] and did not find inclusion of studies with higher identified bias had any effect on analysis trend or significance.

The main findings of the meta-analysis were that individuals with BPPV had lower average serum 25-hydroxyvitamin D levels compared to controls, a finding which was statistically significant ($P = 0.001$). Examining the published literature to date, it is clear that the evidence base regarding the link between vitamin D and BPPV is mixed- with a number of studies included in this review reporting no significant difference between cases with BPPV and controls and others reporting lower serum vitamin D levels in patients with BPPV. These mixed results may reflect that the strength of the relationship between vitamin D and BPPV is relatively weak. Another possibility is that rather than vitamin D having a direct physiological role in the development of BPPV it is an indirect marker of another related biomarker, for example calcium; as calcitriol, manufactured from 25-hydroxyvitamin D, plays a role in regulating intestinal absorption of dietary calcium and bone mineralisation both directly and through interaction with parathyroid hormone.[57]

The effect size in our meta-analysis was relatively small (SMD -2.07), which aligns with other non-geographically restricted systematic reviews performed previously which have established an association between low serum vitamin D and BPPV incidence.[8, 9] It remains unclear however, whether the small absolute difference in serum vitamin D concentration reported between patients with BPPV and those without is physiologically relevant.

The results of our meta-analysis into the association between BPPV recurrence and serum vitamin D levels indicated a similar (SMD -1.90), albeit non- significant association between BPPV recurrence and lower serum vitamin D levels. Previously published, non-geographically restricted systematic reviews have demonstrated a mixed picture relating to the relationship between serum vitamin D and BPPV recurrence with both a negative association,[8, 10] or no impact on recurrence[11] reported.

It might have been expected that restricting our review to include only studies conducted in the northern hemisphere would result in a larger effect size, as more northerly latitudes have previously been shown to be a risk factor for vitamin D deficiency.[58, 59] However, as vitamin D deficiency is more prevalent in such countries during the winter,[60] this may have been confounded by the fact that most of the included studies did not include temporal data relating to the month and/or seasons when vitamin D levels were measured or BPPV occurred. Some of the articles included in this review indicated mixed evidence for a potential relationship between serum vitamin D levels and sub-types of BPPV. Two articles[47, 52] found lower serum vitamin D levels in canalithiasis compared to

For peer review only - http://bmjopen.bmj.com/site/about/guidelines.xhtml

cupulolithiasis, whilst a further two found no significant difference in vitamin D levels between the BPPV sub-types.[29, 53] It was not possible to perform a meta-analysis on this effect due to the small number of studies investigating this relationship, however this could be a parameter for future studies investigating BPPV and vitamin D to explore further. Similarly, the potential association between BPPV, vitamin D and bone mineral density identified by some of the articles[2, 43] could not be further analysed with meta-analysis but could be an area of interest for future research.

It was not possible to perform a meta-analysis examining the seasonality of both BPPV and vitamin D as only two studies reported vitamin D levels according to month or season.[16, 36] This is an important potential confounding factor, as vitamin D levels have been observed to be at their lowest during months when the incidence of BPPV is greatest.[18] One hypothesis is that higher levels of vitamin D may offer a protective effect against BPPV during the summer months, and that the decrease in vitamin D levels over the winter months results in the increased incidence of BPPV seen during these months. Future research should separate individuals by month or season of admission (summer and winter-spring) and examine the difference in serum vitamin D levels between patients and controls during these time periods.

In conclusion, our results indicate that low serum vitamin D levels are correlated with the incidence of BPPV, whilst a non-significant association was reported in recurrent BPPV compared to non-recurrent disease. It was not possible to further investigate the seasonal relationships of BPPV and vitamin D deficiency as most studies do not include data relating to when patients are seen, and this is an area of interest for future research. Investigating if serum vitamin D levels relate to sub-types of BPPV and whether restoring vitamin D levels to sufficient levels via supplementation offers protection against BPPV are other topics which would benefit from further studies. As a large proportion of the population of countries in northern hemisphere countries are at a greater risk of vitamin D deficiency, patients developing BPPV in these countries may benefit from opportunistic serum vitamin D testing and/or supplementation.

For peer review only

Figure Legends:

Figure 1. *Flow diagram detailing results of searches and study selection process*

Figure 2. Relationship between serum 25-hydroxyvitamin D and Benign Paroxysmal Positional Vertigo incidence.

Figure 3. Relationship between serum 25-hydroxyvitamin D and Benign Paroxysmal Positional Vertigo recurrence.

For peer review only

Funding: KK was supported by the NIHR Manchester Biomedical Research Centre, UK IS-BRC-1215-20007, NIHR203308 and MRC Programme Grants MR/L003589/1, MR/V01272X/1.

Competing interests: None to declare

Data Sharing: No additional data.

Contributorship statement: HW, KK and GB conceptualised, planned, and designed this Systematic Review and Meta-Analyses. HW conducted the searches and initial screening of titles and abstracts. HW and GB independently reviewed the full texts and conducted the assessment of bias. HW extracted the data, and this was verified by GB, disagreements at any stage were resolved by discussion and by involvement of KK. HW and GB interpreted the results, and this was reviewed by KK. HW wrote the original manuscript. GB and KK were responsible for supervision, administration, reviewing, and editing the manuscript. All authors reviewed the results and approved the final version of the manuscript.

For peer review only

References

1. Talaat HS, Abuhadied G, Talaat AS, et al. Low bone mineral density and vitamin D deficiency in patients with benign positional paroxysmal vertigo. *European archives of oto-rhino-laryngology : official journal of the European Federation of Oto-Rhino-Laryngological Societies (EUFOS) : affiliated with the German Society for Oto-Rhino-Laryngology - Head and Neck Surgery* 2015;272(9):2249-53. doi: 10.1007/s00405-014-3175-3 [published Online First: 2014/06/30]

2. Lee SB, Lee CH, Kim YJ, et al. Biochemical markers of bone turnover in benign paroxysmal positional vertigo. *PloS one* 2017;12(5):e0176011. doi: 10.1371/journal.pone.0176011 [published Online First: 2017/05/04]

3. Büki B, Jünger H, Zhang Y, et al. The Price of Immune Responses and the Role of Vitamin D in the Inner Ear. *Otology & neurotology : official publication of the American Otological Society, American Neurotology Society [and] European Academy of Otology and Neurotology* 2019;40(6):701-09. doi: 10.1097/mao.0000000000002258 [published Online First: 2019/06/14]

4. Borel P, Caillaud D, Cano NJ. Vitamin D Bioavailability: State of the Art. *Critical Reviews in Food Science and Nutrition* 2015;55(9):1193-205. doi: 10.1080/10408398.2012.688897

5. Alshahrani F, Aljohani N. Vitamin D: deficiency, sufficiency and toxicity. *Nutrients* 2013;5(9):3605-16. doi: 10.3390/nu5093605 [published Online First: 20130913]

6. You P, Instrum R, Parnes L. Benign paroxysmal positional vertigo. *Laryngoscope investigative otolaryngology* 2019;4(1):116-23. doi: 10.1002/lio2.230 [published Online First: 2019/03/05]

7. Chen J, Zhao W, Yue X, et al. Risk Factors for the Occurrence of Benign Paroxysmal Positional Vertigo: A Systematic Review and Meta-Analysis. *Frontiers in neurology* 2020;11:506. doi: 10.3389/fneur.2020.00506 [published Online First: 2020/07/14]

8. Yang B, Lu Y, Xing D, et al. Association between serum vitamin D levels and benign paroxysmal positional vertigo: a systematic review and meta-analysis of observational studies. *European archives of oto-rhino-laryngology : official journal of the European Federation of Oto-Rhino-Laryngological Societies (EUFOS) : affiliated with the German Society for Oto-Rhino-Laryngology - Head and Neck Surgery* 2020;277(1):169-77. doi: 10.1007/s00405-019-05694-0 [published Online First: 2019/10/21]

9. AlGarni MA, Mirza AA, Althobaiti AA, et al. Association of benign paroxysmal positional vertigo with vitamin D deficiency: a systematic review and meta-analysis. *European archives of oto-rhino-laryngology : official journal of the European Federation of Oto-Rhino-Laryngological Societies (EUFOS) : affiliated with the German Society for Oto-Rhino-Laryngology - Head and Neck Surgery* 2018;275(11):2705-11. doi: 10.1007/s00405-018-5146-6 [published Online First: 2018/10/12]

10. Chen J, Zhang S, Cui K, et al. Risk factors for benign paroxysmal positional vertigo recurrence: a systematic review and meta-analysis. *Journal of neurology* 2020 doi: 10.1007/s00415-020-10175-0 [published Online First: 2020/08/26]

11. Li S, Wang Z, Liu Y, et al. Risk Factors for the Recurrence of Benign Paroxysmal Positional Vertigo: A Systematic Review and Meta-Analysis. *Ear, nose, & throat journal* 2022;101(3):NP112-NP34. doi: 10.1177/0145561320943362 [published Online First: 2020/08/11]

12. Sheikhzadeh M, Lotfi Y, Mousavi A, et al. Influence of supplemental vitamin D on intensity of benign paroxysmal positional vertigo: A longitudinal clinical study. *Caspian journal of internal medicine* 2016;7(2):93-8. [published Online First: 2016/07/08]

13. Yang Z, Li J, Zhu Z, et al. Effect of vitamin D supplementation on benign paroxysmal positional vertigo recurrence: A meta-analysis. *Science progress* 2021;104(2):368504211024569. doi: 10.1177/00368504211024569 [published Online First: 2021/06/16]

14. Cashman KD, Dowling KG, Škrabáková Z, et al. Vitamin D deficiency in Europe: pandemic? *The American journal of clinical nutrition* 2016;103(4):1033-44. doi: 10.3945/ajcn.115.120873 [published Online First: 2016/02/13]

15. Manios Y, Moschonis G, Lambrinou CP, et al. A systematic review of vitamin D status in southern European countries. *European journal of nutrition* 2018;57(6):2001-36. doi: 10.1007/s00394-017-1564-2 [published Online First: 2017/11/02]

16. Shu L, Wu J, Jiang CY, et al. Seasonal variation of idiopathic benign paroxysmal positional vertigo correlates with serum 25-hydroxyvitamin D levels: a six-year registry study in Shanghai, China. *Scientific reports* 2019;9(1):16230. doi: 10.1038/s41598-019-52803-4 [published Online First: 2019/11/09]

17. Sideris G, Sapountzi MC, Malamas V, et al. Climatic Variations as Indicators of Vitamin D Levels and Benign Paroxysmal Positional Vertigo. *Cureus* 2021;13(10):e18811. doi: 10.7759/cureus.18811 [published Online First: 2021/11/23]

18. Whitman GT, Baloh RW. Seasonality of benign paroxysmal positional vertigo. *JAMA otolaryngology-- head & neck surgery* 2015;141(2):188-9. doi: 10.1001/jamaoto.2014.2941 [published Online First: 2014/11/21]

19. Jeong J, Eo TS, Oh J, et al. Monthly and seasonal variations in benign paroxysmal positional vertigo. *Journal of vestibular research : equilibrium & orientation* 2021;31(2):101-07. doi: 10.3233/ves-200030 [published Online First: 2021/02/09]

20. Seidel DU, Park JJ, Sesterhenn AM, et al. Demographic data and seasonal variation in peripheral vestibular disorders in ENT practices in Germany. *Journal of vestibular research : equilibrium & orientation* 2019;29(4):181-90. doi: 10.3233/ves-190668 [published Online First: 2019/07/01]

21. Higgins JPT, Thomas J, Chandler J, et al. Cochrane Handbook for Systematic Reviews of Interventions version 6.4 (updated August 2023) 2023 [Available from: Available from www.training.cochrane.org/handbook.2023.

For peer review only - http://bmjopen.bmj.com/site/about/guidelines.xhtml

22. Page MJ, McKenzie JE, Bossuyt PM, et al. The PRISMA 2020 statement: an updated guideline for reporting systematic reviews. *BMJ* 2021;372:n71. doi: 10.1136/bmj.n71
23. Kim SY, Park JE, Lee YJ, et al. Testing a tool for assessing the risk of bias for nonrandomized studies showed moderate reliability and promising validity. *Journal of clinical epidemiology* 2013;66(4):408-14. doi: 10.1016/j.jclinepi.2012.09.016 [published Online First: 2013/01/23]
24. Wells GA, Shea B, O'Connell D, et al. The Newcastle-Ottawa Scale (NOS) for assessing the quality of nonrandomised studies in meta-analyses 2018 [Available from: http://www.ohri.ca/programs/clinical_epidemiology/oxford.asp accessed 21.05.2021 2021.
25. Holick MF. Vitamin D deficiency. *The New England journal of medicine* 2007;357(3):266-81. doi: 10.1056/NEJMra070553 [published Online First: 2007/07/20]
26. Bi J, Liu B, Zhang Y, et al. Study on the Bone Metabolism Indices and Otoconin-90 in Benign Paroxysmal Positional Vertigo. *Otology & neurotology : official publication of the American Otological Society, American Neurotology Society [and] European Academy of Otology and Neurotology* 2021;42(6):e744-e49. doi: 10.1097/mao.0000000000003087 [published Online First: 2021/02/20]
27. Çelik H, Yardim A, Ertaş A, et al. Evaluation of Serum 25-Hydroxyvitamin Vitamin D, Vitamin B12, and Folate Levels in Patients with Benign Paroxysmal Positional Vertigo. . *Eurasian Journal of Emergency Medicine* 2021;20(1):19-24. doi: 10.4274/eajem.galenos.2020.88942
28. Ding J, Liu L, Kong WK, et al. Serum levels of 25-hydroxy vitamin D correlate with idiopathic benign paroxysmal positional vertigo. *Bioscience reports* 2019;39(4) doi: 10.1042/bsr20190142 [published Online First: 2019/04/10]
29. Han K, Yun YM, Moon SG, et al. Bone mineral density and serum 25-hydroxyvitamin D in subtypes of idiopathic benign paroxysmal positional vertigo. *American journal of otolaryngology* 2020;41(1):102313. doi: 10.1016/j.amjoto.2019.102313 [published Online First: 2019/11/17]
30. Inan HC, Mertoğlu C, Erdur ZB. Investigation of Serum Calcium and 25-Hydroxy Vitamin D Levels in Benign Paroxysmal Positional Vertigo Patients. *Ear, nose, & throat journal* 2021;100(9):643-46. doi: 10.1177/0145561321989451 [published Online First: 2021/01/26]
31. Sarsitthithum K, Wisupagan T, Kiatthanabumrung S, et al. The Association Between Serum Vitamin D Levels and Benign Paroxysmal Positional Vertigo. *Ear, nose, & throat journal* 2021:1455613211008561. doi: 10.1177/01455613211008561 [published Online First: 2021/04/20]
32. Sen K, Padiyar BV, Arora G. Association of Benign Paroxysmal Positional Vertigo with Osteoporosis and Vitamin D Deficiency – A Case-Control Study. *Dubai Medical Journal* 2018;1:2-5. doi: 10.1159/000492756
33. Wang Z, Yao G, Tao X, et al. Evaluation of bone mineral density and 25-(OH) vitamin D levels in middle-aged and elderly women with recurrent benign paroxysmal positional vertigo. *Acta oto-laryngologica* 2020;140(2):89-93. doi: 10.1080/00016489.2019.1692146 [published Online First: 2019/12/27]
34. Wu Y, Fan Z, Jin H, et al. Assessment of Bone Metabolism in Male Patients With Benign Paroxysmal Positional Vertigo. *Frontiers in neurology* 2018;9:742. doi: 10.3389/fneur.2018.00742 [published Online First: 2018/09/21]
35. Zhang X, Zhang Z, Lv X. Predictive values of serum estradiol, calcium, and 25-hydroxyvitamin D levels for recurrence of benign paroxysmal positional vertigo in postmenopausal women. *Turk J Phys Med Rehabil* 2022;68(1):30-36. doi: 10.5606/tftrd.2022.5964 [published Online First: 20220301]
36. Califano L, Salafia F, Melillo MG, et al. Is hypovitaminosis D a risk factor for either the onset or the recurrence of Benign Paroxysmal Positional Vertigo? . *Frontiera ORL* 2019
37. Ceylan SM, Kanmaz MA. Vitamin D Influence on the Clinical Course of Benign Paroxysmal Positional Vertigo: A Retrospective Cohort Study. *Otorhinolaryngology Clinics: An International Journal* 2020;12(3):43-47.
38. Çikrikçi Işik G, Cevik Y, Emektar E, et al. Analysis of Vitamin D and Calcium Levels in Benign Paroxysmal Positional Vertigo. *Eurasian Journal of Emergency Medicine* 2017;16:128-32. doi: 10.5152/eajem.2017.58077
39. Goldschagg N, Teupser D, Feil K, et al. No evidence for a specific vitamin D deficit in benign paroxysmal positional vertigo. *European journal of neurology* 2021;28(9):3182-86. doi: 10.1111/ene.14980 [published Online First: 2021/06/17]
40. Karataş A, Acar Yuceant G, Yuce T, et al. Association of Benign Paroxysmal Positional Vertigo with Osteoporosis and Vitamin D Deficiency: A Case Controlled Study. *The journal of international advanced otology* 2017;13(2):259-65. doi: 10.5152/iao.2016.2640 [published Online First: 2017/03/10]
41. Pecci R, Mandalà M, Marcari A, et al. Vitamin D Insufficiency/Deficiency in Patients with Recurrent Benign Paroxysmal Positional Vertigo. *The journal of international advanced otology* 2022;18(2):158-66. doi: 10.5152/iao.2022.21269
42. Thomas RJ, Goutham MK, Bhat VS, et al. Association of Serum Calcium and Vitamin D with Benign Paroxysmal Positional Vertigo. *Int Arch Otorhinolaryngol* 2022;26(3):e365-e69. doi: 10.1055/s-0041-1724093 [published Online First: 20211103]
43. Parham K, Leonard G, Feinn RS, et al. Prospective clinical investigation of the relationship between idiopathic benign paroxysmal positional vertigo and bone turnover: a pilot study. *The Laryngoscope* 2013;123(11):2834-9. doi: 10.1002/lary.24162 [published Online First: 2013/05/16]
44. Wu Y, Han K, Han W, et al. Low 25-Hydroxyvitamin D Levels Are Associated With Residual Dizziness After Successful Treatment of Benign Paroxysmal Positional Vertigo. *Frontiers in neurology* 2022;13:915239. doi: 10.3389/fneur.2022.915239 [published Online First: 20220622]

For peer review only - http://bmjopen.bmj.com/site/about/guidelines.xhtml

45. Melis A, Rizzo D, Gallus R, et al. Relationship between calcium metabolism and benign paroxysmal positional vertigo in north Sardinia population. *Journal of vestibular research : equilibrium & orientation* 2020;30(6):375-82. doi: 10.3233/ves-200025
46. Shin HI, Park Y, Lee HJ, et al. Correlation between serum vitamin D level and benign paroxysmal positional vertigo recurrence. *Auris Nasus Larynx* 2023 doi: 10.1016/j.anl.2022.12.017 [published Online First: 20230123]
47. Maslovara S, Butkovic Soldo S, Sestak A, et al. 25 (OH) D3 levels, incidence and recurrence of different clinical forms of benig paroxysmal positional vertigo. *Brazilian journal of otorhinolaryngology* 2018;84(4):453-59. doi: 10.1016/j.bjorl.2017.05.007 [published Online First: 2017/06/29]
48. Yang CJ, Kim Y, Lee HS, et al. Bone mineral density and serum 25-hydroxyvitamin D in patients with idiopathic benign paroxysmal positional vertigo. *Journal of vestibular research : equilibrium & orientation* 2018;27(5-6):287-94. doi: 10.3233/ves-170625 [published Online First: 2018/02/06]
49. Carneiro de Sousa PJM, Abreu Pereira DM, Carneiro Melo Pereira de Magalhães P, et al. Vitamin D deficiency and benign paroxysmal positioning vertigo. *Hearing, Balance and Communication* 2019;17(2):179-81. doi: 10.1080/21695717.2019.1590988
50. Elmoursy MM, Abbas AS. The role of low levels of vitamin D as a co-factor in the relapse of benign paroxysmal positional vertigo (BPPV). *American journal of otolaryngology* 2021;42(6):103134. doi: 10.1016/j.amjoto.2021.103134 [published Online First: 2021/06/25]
51. Gu X, Dong F, Gu J. Analysis of effect of 1α-hydroxyvitamin D3 on benign paroxysmal positional vertigo and risk factors. *Experimental and therapeutic medicine* 2018;15(3):2321-26. doi: 10.3892/etm.2018.5699 [published Online First: 2018/02/20]
52. Nakada T, Sugiura S, Uchida Y, et al. Difference in Serum Levels of Vitamin D Between Canalolithiasis and Cupulolithiasis of the Horizontal Semicircular Canal in Benign Paroxysmal Positional Vertigo. *Frontiers in neurology* 2019;10:176. doi: 10.3389/fneur.2019.00176 [published Online First: 2019/03/19]
53. Han WW, Wu YQ, Fan ZY, et al. Characteristics of bone metabolism in postmenopausal female patients with different types of idiopathic benign paroxysmal positional vertigo: A single-centre retrospective study. *American journal of otolaryngology* 2021;42(6):103149. doi: 10.1016/j.amjoto.2021.103149 [published Online First: 2021/07/10]
54. Kahraman SS, Ozcan O, Arli C, et al. Calcium Homeostasis During Attack and Remission in Patients With Idiopathic Benign Paroxysmal Positional Vertigo. *Otology & neurotology : official publication of the American Otological Society, American Neurotology Society [and] European Academy of Otology and Neurotology* 2016;37(9):1388-92. doi: 10.1097/mao.0000000000001167 [published Online First: 2016/08/16]
55. Song P, Zhao X, Xu Y, et al. Correlation Between Benign Paroxysmal Positional Vertigo and 25-hydroxyvitamin D. *Frontiers in neurology* 2020;11:576. doi: 10.3389/fneur.2020.00576 [published Online First: 2020/07/14]
56. Rhim GI. Serum Vitamin D and Long-term Outcomes of Benign Paroxysmal Positional Vertigo. *Clin Exp Otorhinolaryngol* 2019;12(3):273-78. doi: 10.21053/ceo.2018.00381 [published Online First: 20190301]
57. Fleet JC. The role of vitamin D in the endocrinology controlling calcium homeostasis. *Molecular and cellular endocrinology* 2017;453:36-45. doi: 10.1016/j.mce.2017.04.008 [published Online First: 2017/04/13]
58. Leary PF, Zamfirova I, Au J, et al. Effect of Latitude on Vitamin D Levels. *J Am Osteopath Assoc* 2017;117(7):433-39. doi: 10.7556/jaoa.2017.089
59. Oskarsson V, Eliasson M, Salomaa V, et al. Influence of geographical latitude on vitamin D status: cross-sectional results from the BiomarCaRE consortium. *British Journal of Nutrition* 2022;128(11):2208-18. doi: 10.1017/S0007114521005080 [published Online First: 2021/12/22]
60. Huotari A, Herzig KH. Vitamin D and living in northern latitudes--an endemic risk area for vitamin D deficiency. *International journal of circumpolar health* 2008;67(2-3):164-78. doi: 10.3402/ijch.v67i2-3.18258 [published Online First: 2008/09/05]

For peer review only - http://bmjopen.bmj.com/site/about/guidelines.xhtml

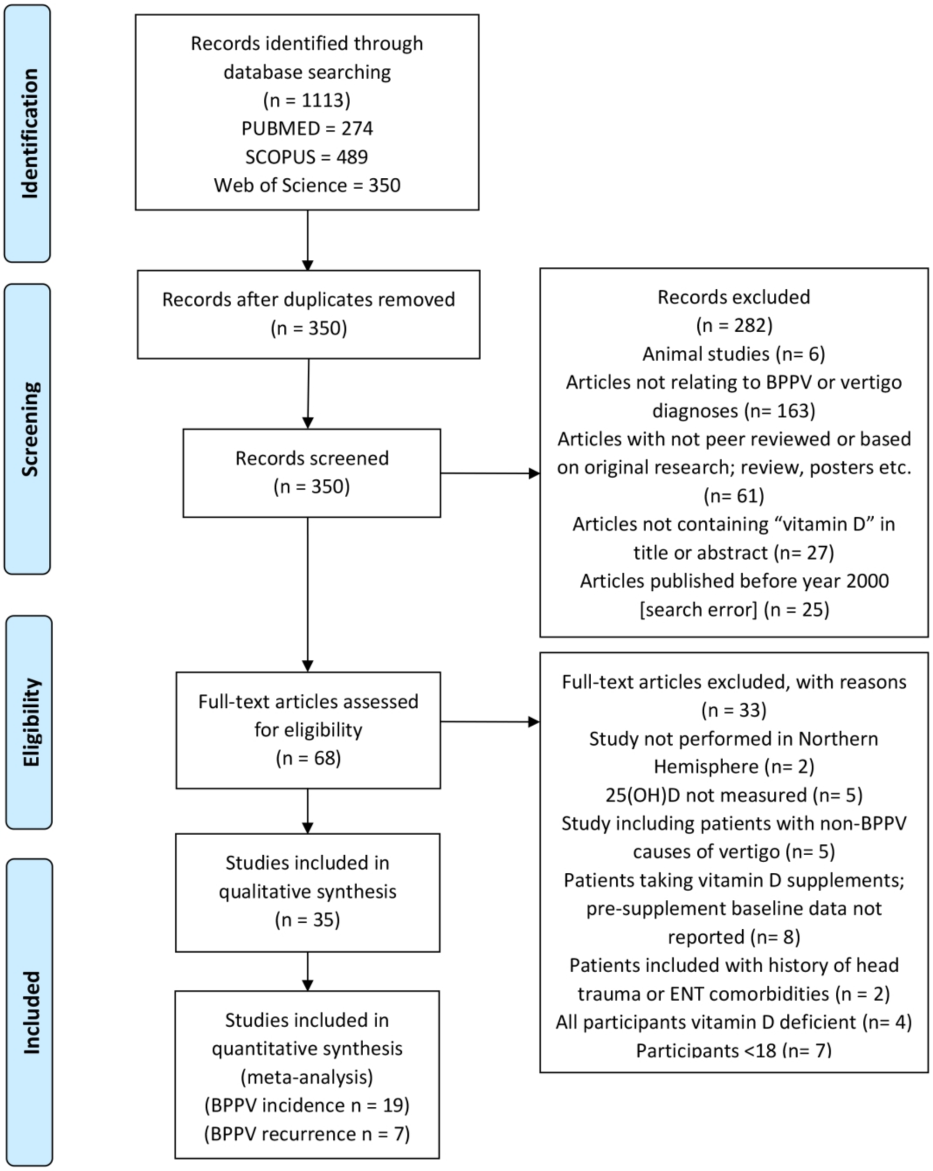

Figure 1. Flow diagram detailing results of searches and study selection process

171x216mm (300 x 300 DPI)

Study or Subgroup	BPPV Mean	BPPV SD	BPPV Total	Control Mean	Control SD	Control Total	Weight	Mean Difference IV, Random, 95% CI
Bi et al 2021	14.64	6.94	27	19.56	6.55	25	4.2%	-4.92 [-8.59, -1.25]
Califano et al 2019	21.08	11	127	25.6	10.5	100	4.9%	-4.52 [-7.33, -1.71]
Çelik et al 2021	15.64	8.4	190	16.29	6.7	149	5.9%	-0.65 [-2.26, 0.96]
Ceylan and Kanmaz 2020	15.87	8.61	97	15.82	10.23	100	5.1%	0.05 [-2.59, 2.69]
Cheng et al 2021	23.2	4.09	320	25.8	3.43	320	6.5%	-2.60 [-3.18, -2.02]
Çıkrıkçı Işık et al 2017	9.51	5.49	64	11.02	9.62	63	5.0%	-1.51 [-4.24, 1.22]
Goldschagg et al 2021	23.4	9.4	158	21.5	10.6	301	5.7%	1.90 [0.01, 3.79]
Han et al 2018	19.1	5.2	85	22.5	5.8	80	5.9%	-3.40 [-5.08, -1.72]
Inan et al 2021	15.3	9.8	52	20.2	14.3	52	3.4%	-4.90 [-9.61, -0.19]
Karatas et al 2017	23	14.4	78	17	12.3	78	3.8%	6.00 [1.80, 10.20]
Sarsitthithim et al 2021	21.5	5.3	69	26.3	6.8	68	5.6%	-4.80 [-6.84, -2.76]
Sen, Padiyar and Arora 2018	20.3	12.2	100	18	10.1	100	4.7%	2.30 [-0.80, 5.40]
Song et al 2020	14.24	6.6	380	15.45	7.7	3125	6.4%	-1.21 [-1.93, -0.49]
Talaat et al 2015	14.19	9.3	80	19.53	8.45	100	5.1%	-5.34 [-7.97, -2.71]
Thomas et al 2021	21.3	9.567128	49	17.6	8.061036	49	4.3%	3.70 [0.20, 7.20]
Wang et al 2020	17.15	2.03	103	23.85	3.13	80	6.4%	-6.70 [-7.49, -5.91]
Wu et al 2018	20.99	6.76	60	23.17	6.49	92	5.5%	-2.18 [-4.34, -0.02]
Yang et al 2017	18.21	10.3	130	20	8.1	130	5.4%	-1.79 [-4.04, 0.46]
Zhang et al 2022	18.8	2.5	156	24	2.2	50	6.4%	-5.20 [-5.93, -4.47]
**Total (95% CI)**			**2325**			**5062**	**100.0%**	**-2.07 [-3.32, -0.81]**

Heterogeneity: Tau² = 6.22; Chi² = 238.02, df = 18 (P < 0.00001); I² = 92%

Test for overall effect: Z = 3.24 (P = 0.001)

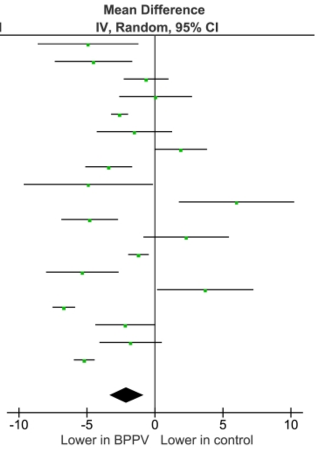

Figure 2. Relationship between serum 25-hydroxyvitamin D and Benign Paroxysmal Positional Vertigo incidence.

321x146mm (300 x 300 DPI)

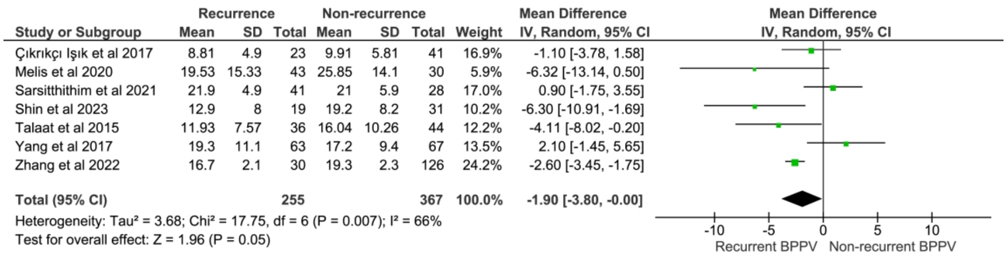

Figure 3. Relationship between serum 25-hydroxyvitamin D and Benign Paroxysmal Positional Vertigo recurrence.

303x78mm (300 x 300 DPI)

NIHR | National Institute for Health Research

PROSPERO
International prospective register of systematic reviews

Association between vitamin D deficiency and benign paroxysmal positional vertigo: a systematic review and meta-analysis

Citation

Heather Wood, Ghada BinKhamis, Karolina Kluk De-Kort. Association between vitamin D deficiency and benign paroxysmal positional vertigo: a systematic review and meta-analysis. PROSPERO 2021 CRD42021271840 Available from: https://www.crd.york.ac.uk/prospero/display_record.php?ID=CRD42021271840

For peer review only

Review question

How do serum vitamin D levels affect the incidence and recurrence rates of benign paroxysmal positional vertigo (BPPV) in adult patients in the Northern Hemisphere?

Searches

We will search the following electronic bibliographic databases: PubMed, NICE, Scopus, Web of Science using the following search terms: "Vitamin D* OR 25-hydroxyvitamin D OR ergocalciferol OR cholecalciferol AND benign paroxysmal positional vertigo", "Vitamin D* OR 25-hydroxyvitamin D OR ergocalciferol OR cholecalciferol AND BPPV" "Vitamin D* OR 25-hydroxyvitamin D OR ergocalciferol OR cholecalciferol AND vertigo"

Research papers published between 2000 and 2021 will be reviewed and language is restricted with English.

- Searches will be re-run prior to the final analysis to identify any further studies.

- Unpublished studies will not be sought

Types of study to be included

Randomised controlled trial, cohort study or case-controlled study

Condition or domain being studied

Benign Paroxysmal positional vertigo (BPPV) and serum vitamin D levels

Participants/population [2 changes]

Inclusion criteria:

- Participants over the age of 18 with at least one episode of diagnosed benign paroxysmal positional vertigo (BPPV).

- Serum 25-hydroxyvitamin D levels measured and reported.

- Geographical location of study participants in Northern hemisphere.

- Participants not taking vitamin D supplements,

- No other comorbid diagnoses.

Exclusion criteria:

- Participants below the age of 18 with at least one episode of BPPV.

- Serum 25-hydroxyvitamin D levels not measured or reported.

- Geographical location of study participants not in Northern hemisphere.

- Participants taking vitamin D supplements,

- Participants with other comorbid diagnoses.

Intervention(s), exposure(s)

Serum 25-hydroxyvitamin D levels

Comparator(s)/control

Normal controls over the age of 18 with no past medical history of benign paroxysmal positional vertigo

Context

Studies in hospital ENT outpatient departments. Research including participants in southern hemisphere countries will be excluded

Main outcome(s)

Incidence of benign paroxysmal positional vertigo. Recurrence of benign paroxysmal positional vertigo

Measures of effect

Difference in average serum vitamin D levels between patients with new or recurrent benign paroxysmal positional vertigo compared to controls.

Additional outcome(s)

Not applicable

Data extraction (selection and coding)

Study selection: one author will select and this selection will be reviewed by two authors.

The final decision will be recorded in an excel spreadsheet.

Any disagreements will be resolved by discussion between the authors.

Extracted data will include:

Study design; country; author and published year; mean age of participants; range of participant age; sample size; disease diagnosis; percentage male participants and serum vitamin D level for cases and controls.

Risk of bias (quality) assessment

For peer review only

Any randomised controlled trials will be assessed using the Cochrane Collaboration's tool for assessing risk of bias in randomised trials.

Case-control studies will be assessed using the Risk of Bias Assessment Tool for Nonrandomized Studies (RoBANS) tool.

Cohort studies will be assessed according to the Newcastle Ottawa Scale (NOS).

Records of risk of bias will be recorded in Microsoft Word and Microsoft Excel files.

Strategy for data synthesis

Standardised mean difference (SMD) with a 95% confidence interval will be used to calculate the level of vitamin D in case and control groups. Plasma vitamin D levels of greater than 20 ng/ml will be considered to be sufficient. The Q statistic will be used to examine heterogeneity between studies, with P<0.10 regarded as significant. The random-effects model will be employed for the meta-analyses if P<0.10, otherwise the fixed-effect model will be used instead.

Statistical analysis will be completed using RevMan software version 5.4.1

Analysis of subgroups or subsets

Vitamin D status will be categorised into "sufficient" if serum levels are >20ng/l; or "insufficient" if serum levels <20ng/L

There may be differences between patients experiencing recurrent BPPV and those experiencing single episodes. Recurrent BPPV will be classified as symptom recurrence within 3 years of initial diagnosis.

Contact details for further information

Heather Wood

hmw998@student.bham.ac.uk

Organisational affiliation of the review

University of Birmingham

Review team members and their organisational affiliations [1 change]

Ms Heather Wood. University of Birmingham, Birmingham, UK

Dr Ghada BinKhamis. University of Manchester, Manchester, UK. King Fahad Medical City, Riyadh, Saudi Arabia

Dr Karolina Kluk De-Kort. University of Manchester, Manchester, UK

Type and method of review

Meta-analysis, Systematic review

Anticipated or actual start date

09 August 2021

For peer review only

Anticipated completion date [1 change]

01 September 2023

Funding sources/sponsors

None

Conflicts of interest

Language

English

Country

England

Stage of review

Review Ongoing

Subject index terms status

Subject indexing assigned by CRD

Subject index terms

Benign Paroxysmal Positional Vertigo; Humans; Vitamin D Deficiency

Date of registration in PROSPERO

06 August 2021

Date of first submission

04 August 2021

Stage of review at time of this submission [3 changes]

For peer review only

NIHR | National Institute for Health Research

PROSPERO
International prospective register of systematic reviews

Stage	Started	Completed
Preliminary searches	Yes	Yes
Piloting of the study selection process	Yes	Yes
Formal screening of search results against eligibility criteria	Yes	Yes
Data extraction	Yes	No
Risk of bias (quality) assessment	Yes	Yes
Data analysis	Yes	No

Revision note

Updating progress to date - checked boxes to indicate that screening and assessment of bias are complete and that data extraction and analysis have been started.

The record owner confirms that the information they have supplied for this submission is accurate and complete and they understand that deliberate provision of inaccurate information or omission of data may be construed as scientific misconduct.

The record owner confirms that they will update the status of the review when it is completed and will add publication details in due course.

Versions

06 August 2021

09 August 2021

14 September 2021

27 October 2021

21 February 2023

01 April 2023

For peer review only

Supplementary 2

Supplementary 2, Table 1. Systematic review searches performed and results.

Search	*Search terms*	*Database*	*Results*	*Filters*
**1**	(Vitamin D OR 25-hydroxyvitamin D OR ergocalciferol OR cholecalciferol) AND BPPV	PUBMED	86	Years 2000 - 2023, English Language
**2**	(Vitamin D OR 25-hydroxyvitamin D OR ergocalciferol OR cholecalciferol) AND "Benign paroxysmal positional vertigo"	PUBMED	86	Years 2000 - 2023, English Language
**3**	(Vitamin D OR 25-hydroxyvitamin D OR ergocalciferol OR cholecalciferol) AND vertigo	PUBMED	102	Years 2000 - 2023, English Language
**4**	(Vitamin D* OR 25-hydroxyvitamin D OR ergocalciferol OR cholecalciferol) AND BPPV	SCOPUS	85	Year >1999, English Language
**5**	(Vitamin D* OR 25-hydroxyvitamin D OR ergocalciferol OR cholecalciferol) AND "Benign paroxysmal positional vertigo"	SCOPUS	104	Year >1999, English Language
**6**	(Vitamin D* OR 25-hydroxyvitamin D OR ergocalciferol OR cholecalciferol) AND "vertigo"	SCOPUS	300	Year >1999, English Language
**7**	(ALL=(Vitamin D OR 25-hydroxyvitamin D OR ergocalciferol OR cholecalciferol)) AND ALL=(BPPV)	Web of Science	101	Timespan: 2000-01-01 to2023-02-18, English Language
**8**	(ALL=(Vitamin D OR 25-hydroxyvitamin D OR ergocalciferol OR cholecalciferol)) AND ALL=(Benign Paroxysmal Positional Vertigo)	Web of Science	108	Timespan: 2000-01-01 to 2023-02-18, English Language
**9**	(ALL=(Vitamin D OR 25-hydroxyvitamin D OR ergocalciferol OR cholecalciferol)) AND ALL=(Vertigo)	Web of Science	141	Timespan: 2000-01-01 to2023-02-18, English Language

For peer review only

Supplementary 2, Table 2. Data extracted from included studies for the Meta-analysis exploring the relationship between Vitamin D deficiency and BPPV occurrence.

	Participants with BPPV			Control participants (without BPPV)		
**Study**	**Number of Participants**	**Vitamin D (Mean: ng/mL)**	**Vitamin D (SD: ng/mL)**	**Number of Participants**	**Vitamin D (Mean: ng/mL)**	**Vitamin D (SD: ng/mL)**
Bi et al. 2021	27	14.64	6.94	25	19.56	6.55
Califano et al. 2019	127	21.08	11	100	25.6	10.5
Çelik et al. 2021	190	15.64	8.4	149	16.29	6.7
Ceylan and Kanmaz 2020	97	15.87	8.61	100	15.82	10.23
Cheng et al. 2021	320	23.2	4.09	320	25.8	3.43
Çıkrıkçı Işık et al. 2017	64	9.51	5.49	63	11.02	9.62
Goldschagg et al. 2021	158	23.4	9.4	301	21.5	10.6
Han et al. 2018	85	19.1	5.2	80	22.5	5.8
Inan et al. 2021	52	15.3	9.8	52	20.2	14.3
Karataş et al. 2017	78	23	14.4	78	17	12.3
Sarsitthithim et al. 2021	69	21.5	5.3	68	26.3	6.8
Sen, Padiyar and Arora 2018	100	20.3	12.2	100	18	10.1
Song et al. 2020	380	14.24	6.6	3125	15.45	7.7
Talaat et al. 2015	80	14.19	9.3	100	19.53	8.45
Thomas et al. 2021	49	21.3	9.567	49	17.6	8.061
Wang et al. 2020	103	17.15	2.03	80	23.85	3.13
Wu et al. 2018	60	20.99	6.76	92	23.17	6.49
Yang et al. 2017	130	18.21	10.3	130	20	8.1
Zhang et al. 2022	156	18.8	2.5	50	24	2.2

For peer review only

Supplementary 2, Table 3. Data extracted from included studies for the Meta-analysis exploring the relationship between Vitamin D deficiency and BPPV recurrence.

	Participants with BPPV Recurrence			Participants without BPPV Recurrence		
**Study**	**Number of Participants**	**Vitamin D (Mean: ng/mL)**	**Vitamin D (SD: ng/mL)**	**Number of Participants**	**Vitamin D (Mean: ng/mL)**	**Vitamin D (SD: ng/mL)**
Çıkrıkçı Işık et al. 2017	23	8.81	4.9	41	9.91	5.81
Melis et al. 2020	43	19.53	15.33	30	25.85	14.1
Sarsitthithim et al. 2021	41	21.9	4.9	28	21	5.9
Shin *et al.* 2023	19	12.9	8	31	19.2	8.2
Talaat et al. 2015	36	11.93	7.57	44	16.04	10.26
Yang et al. 2017	63	19.3	11.1	67	17.2	9.4
Zhang *et al.* 2022	30	16.7	2.1	126	19.3	2.3

For peer review only

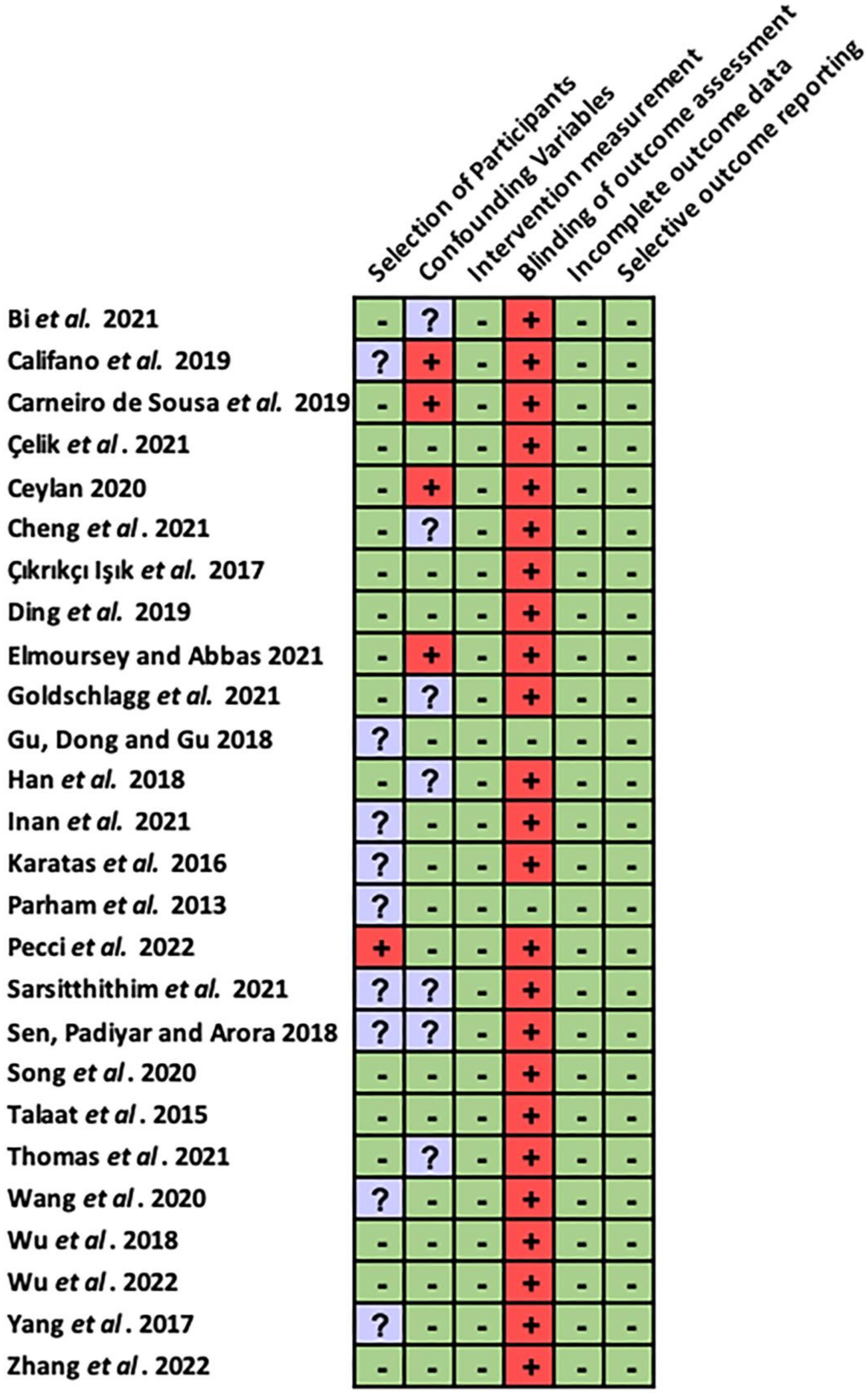

	Selection of Participants	Confounding Variables	Intervention measurement	Blinding of outcome assessment	Incomplete outcome data	Selective outcome reporting
Bi *et al.* 2021	-	?	-	+	-	-
Califano *et al.* 2019	?	+	-	+	-	-
Carneiro de Sousa *et al.* 2019	-	+	-	+	-	-
Çelik *et al*. 2021	-	-	-	+	-	-
Ceylan 2020	-	+	-	+	-	-
Cheng *et al*. 2021	-	?	-	+	-	-
Çıkrıkçı Işık *et al.* 2017	-	-	-	+	-	-
Ding *et al.* 2019	-	-	-	+	-	-
Elmoursey and Abbas 2021	-	+	-	+	-	-
Goldschlagg *et al.* 2021	-	?	-	+	-	-
Gu, Dong and Gu 2018	?	-	-	-	-	-
Han *et al.* 2018	-	?	-	+	-	-
Inan *et al.* 2021	?	-	-	+	-	-
Karatas *et al.* 2016	?	-	-	+	-	-
Parham *et al.* 2013	?	-	-	-	-	-
Pecci *et al.* 2022	+	-	-	+	-	-
Sarsitthithim *et al.* 2021	?	?	-	+	-	-
Sen, Padiyar and Arora 2018	?	?	-	+	-	-
Song *et al*. 2020	-	-	-	+	-	-
Talaat *et al*. 2015	-	-	-	+	-	-
Thomas *et al*. 2021	-	?	-	+	-	-
Wang *et al*. 2020	?	-	-	+	-	-
Wu *et al*. 2018	-	-	-	+	-	-
Wu *et al*. 2022	-	-	-	+	-	-
Yang *et al*. 2017	?	-	-	+	-	-
Zhang *et al*. 2022	-	-	-	+	-	-

Supplementary 2, Figure 1. Assessment of bias for case-control studies according to RoBANs.
Green '-' symbols= low risk of bias, red '+' symbols= high risk of bias and purple '?' symbols = unclear risk of bias.

	Representativeness of exposed cohort	Selection of non-exposed cohort	Ascertainment of exposure	Outcome of interest not present at start	Comparability of cohorts/confounders	Assessment of outcome	Follow-up long enough?	Adequacy of follow-up of cohorts	Study Quality
Han *et al.* 2020	*	*	*	-	**	*	*	n/a	Good
Han *et al*. 2021	-	*	*	-	**	*	*	n/a	Fair
Kahraman *et al.* 2016	*	*	*	-	*	*	*	*	Good
Lee *et al.* 2017	-	*	*	-	**	*	*	n/a	Fair
Maslovara *et al.* 2018	*	*	*	*	**	*	*	*	Good
Melis *et al.* 2020	*	*	*	*	**	*	-	*	Good
Nakada *et al*. 2019	*	*	*	-	-	*	*	n/a	Poor
Shin *et al*. 2023	*	*	*	-	*	*	*	*	Good
Shu *et al*. 2019	*	*	*	-	**	*	*	n/a	Good

Supplementary 2, Figure 2. Assessment of bias of cohort studies using the Newcastle-Ottawa Quality Assessment form for cohort studies.
'*' symbols indicate low risk of bias for a given criteria, '-' symbols indicate the study does not meet criteria for low risk of bias and 'n/a' indicates that the criterion does not apply to a given study (for example retrospective studies do not have a follow-up period by design).

For peer review only - http://bmjopen.bmj.com/site/about/guidelines.xhtml

Supplementary 2, Table 4. PRISMA Checklist

Section and Topic	Item #	Checklist item	Location where item is reported
**TITLE**			
Title	1	Identify the report as a systematic review.	Title page (p1)
**ABSTRACT**			
Abstract	2	See the PRISMA 2020 for Abstracts checklist.	Abstract (p2)
**INTRODUCTION**			
Rationale	3	Describe the rationale for the review in the context of existing knowledge.	Introduction (p4)
Objectives	4	Provide an explicit statement of the objective(s) or question(s) the review addresses.	Introduction (p4)
**METHODS**			
Eligibility criteria	5	Specify the inclusion and exclusion criteria for the review and how studies were grouped for the syntheses.	Methods: Inclusion and Exclusion criteria (p5)
Information sources	6	Specify all databases, registers, websites, organisations, reference lists and other sources searched or consulted to identify studies. Specify the date when each source was last searched or consulted.	Methods: Data sources and search strategy (p5)
Search strategy	7	Present the full search strategies for all databases, registers and websites, including any filters and limits used.	Methods: Data sources and search strategy (p5)
Selection process	8	Specify the methods used to decide whether a study met the inclusion criteria of the review, including how many reviewers screened each record and each report retrieved, whether they worked independently, and if applicable, details of automation tools used in the process.	Methods: Inclusion and Exculsion criteria (p5)
Data collection process	9	Specify the methods used to collect data from reports, including how many reviewers collected data from each report, whether they worked independently, any processes for obtaining or confirming data from study investigators, and if applicable, details of automation tools used in the process.	Methods: Data Extraction (p6)
Data items	10a	List and define all outcomes for which data were sought. Specify whether all results that were compatible with each outcome domain in each study were sought (e.g. for all measures, time points, analyses), and if not, the methods used to decide which results to collect.	Methods: Data Extraction (p6)
	10b	List and define all other variables for which data were sought (e.g. participant and intervention characteristics, funding sources). Describe any assumptions made about any missing or unclear information.	Methods: Data Extraction (p6)
Study risk of bias assessment	11	Specify the methods used to assess risk of bias in the included studies, including details of the tool(s) used, how many reviewers assessed each study and whether they worked independently, and if applicable, details of automation tools used in the process.	Methods: Assessment of bias (p6)
Effect measures	12	Specify for each outcome the effect measure(s) (e.g. risk ratio, mean difference) used in the synthesis or presentation of results.	Methods: Synthesis methods (p6)
Synthesis methods	13a	Describe the processes used to decide which studies were eligible for each synthesis (e.g. tabulating the study intervention characteristics and comparing against the planned groups for each synthesis (item #5)).	Methods: Synthesis methods (p6)
	13b	Describe any methods required to prepare the data for presentation or synthesis, such as handling of missing summary statistics, or data conversions.	Methods: Synthesis methods (p6)

For peer review only - http://bmjopen.bmj.com/site/about/guidelines.xhtml

Section and Topic	Item #	Checklist item	Location where item is reported
	13c	Describe any methods used to tabulate or visually display results of individual studies and syntheses.	Methods: Synthesis methods (p6)
	13d	Describe any methods used to synthesize results and provide a rationale for the choice(s). If meta-analysis was performed, describe the model(s), method(s) to identify the presence and extent of statistical heterogeneity, and software package(s) used.	Methods: Synthesis methods (p6)
	13e	Describe any methods used to explore possible causes of heterogeneity among study results (e.g. subgroup analysis, meta-regression).	Not applicable
	13f	Describe any sensitivity analyses conducted to assess robustness of the synthesized results.	Not applicable
Reporting bias assessment	14	Describe any methods used to assess risk of bias due to missing results in a synthesis (arising from reporting biases).	Not applicable
Certainty assessment	15	Describe any methods used to assess certainty (or confidence) in the body of evidence for an outcome.	Not applicable
**RESULTS**			
Study selection	16a	Describe the results of the search and selection process, from the number of records identified in the search to the number of studies included in the review, ideally using a flow diagram.	Results: Study selection (p6-7); Figure 1. Flow diagram detailing results of searches and study selection process; Supplementary 2. Systematic review searches performed and results
	16b	Cite studies that might appear to meet the inclusion criteria, but which were excluded, and explain why they were excluded.	Results: Study selection (p6-7); Figure 1. Flow diagram detailing results of searches and study selection process; Supplementary 2. Systematic review searches performed and results
Study characteristics	17	Cite each included study and present its characteristics.	Results: Description of included studies (p7-8)
Risk of bias in studies	18	Present assessments of risk of bias for each included study.	Results: Quality assessment (p7); Supplementary 2 Assessment of bias for case-control studies according to RoBANs; Supplementary 2 Assessment of bias of cohort studies using the Newcastle-Ottawa Quality Assessment form for cohort studies

For peer review only

Section and Topic	Item #	Checklist item	Location where item is reported
Results of individual studies	19	For all outcomes, present, for each study: (a) summary statistics for each group (where appropriate) and (b) an effect estimate and its precision (e.g. confidence/credible interval), ideally using structured tables or plots.	Table 1. Characteristics of included studies
Results of syntheses	20a	For each synthesis, briefly summarise the characteristics and risk of bias among contributing studies.	Table 1. Characteristics of included studies<br>Figure 3. Relationship between serum 25-hydroxyvitamin D and Benign Paroxysmal Positional Vertigo recurrence.
	20b	Present results of all statistical syntheses conducted. If meta-analysis was done, present for each the summary estimate and its precision (e.g. confidence/credible interval) and measures of statistical heterogeneity. If comparing groups, describe the direction of the effect.	Results: Meta analysis (p8-9)<br>Figure 3. Relationship between serum 25-hydroxyvitamin D and Benign Paroxysmal Positional Vertigo recurrence.
	20c	Present results of all investigations of possible causes of heterogeneity among study results.	Not applicable
	20d	Present results of all sensitivity analyses conducted to assess the robustness of the synthesized results.	Not applicable
Reporting biases	21	Present assessments of risk of bias due to missing results (arising from reporting biases) for each synthesis assessed.	Not applicable
Certainty of evidence	22	Present assessments of certainty (or confidence) in the body of evidence for each outcome assessed.	Not applicable
**DISCUSSION**			
Discussion	23a	Provide a general interpretation of the results in the context of other evidence.	Discussion (p11-12)
	23b	Discuss any limitations of the evidence included in the review.	Strengths and limitations of the study (p3); Discussion (p11-12)
	23c	Discuss any limitations of the review processes used.	Strengths and limitations (p3)
	23d	Discuss implications of the results for practice, policy, and future research.	Discussion (p11-12)
**OTHER INFORMATION**			Prospero registration number (p.2)
Registration and protocol	24a	Provide registration information for the review, including register name and registration number, or state that the review was not registered.	Abstract: Trial registration (p2)
	24b	Indicate where the review protocol can be accessed, or state that a protocol was not prepared.	Prospero registration number (p.2)
	24c	Describe and explain any amendments to information provided at registration or in the protocol.	Prospero registration number (p.2)
Support	25	Describe sources of financial or non-financial support for the review, and the role of the funders or sponsors in the review.	Funding sources (p14)

For peer review only - http://bmjopen.bmj.com/site/about/guidelines.xhtml

Section and Topic	Item #	Checklist item	Location where item is reported
Competing interests	26	Declare any competing interests of review authors.	Competing interests statement (p14)
Availability of data, code and other materials	27	Report which of the following are publicly available and where they can be found: template data collection forms; data extracted from included studies; data used for all analyses; analytic code; any other materials used in the review.	Methods: Synthesis methods (p6) and Supplementary 2 tables 2 and 3

From: Page MJ, McKenzie JE, Bossuyt PM, Boutron I, Hoffmann TC, Mulrow CD, et al. The PRISMA 2020 statement: an updated guideline for reporting systematic reviews. BMJ 2021;372:n71. doi: 10.1136/bmj.n71

For more information, visit: http://www.prisma-statement.org/

For peer review only

Supplementary 2, Table 5. PRISMA Abstract Checklist

Section and Topic	Item #	Checklist item	Reported (Yes/No)
**TITLE**			
Title	1	Identify the report as a systematic review.	YES
**BACKGROUND**			
Objectives	2	Provide an explicit statement of the main objective(s) or question(s) the review addresses.	YES
**METHODS**			
Eligibility criteria	3	Specify the inclusion and exclusion criteria for the review.	YES
Information sources	4	Specify the information sources (e.g. databases, registers) used to identify studies and the date when each was last searched.	YES
Risk of bias	5	Specify the methods used to assess risk of bias in the included studies.	YES
Synthesis of results	6	Specify the methods used to present and synthesise results.	YES
**RESULTS**			
Included studies	7	Give the total number of included studies and participants and summarise relevant characteristics of studies.	YES
Synthesis of results	8	Present results for main outcomes, preferably indicating the number of included studies and participants for each. If meta-analysis was done, report the summary estimate and confidence/credible interval. If comparing groups, indicate the direction of the effect (i.e. which group is favoured).	YES
**DISCUSSION**			
Limitations of evidence	9	Provide a brief summary of the limitations of the evidence included in the review (e.g. study risk of bias, inconsistency and imprecision).	YES
Interpretation	10	Provide a general interpretation of the results and important implications.	YES
**OTHER**			
Funding	11	Specify the primary source of funding for the review.	YES
Registration	12	Provide the register name and registration number.	YES

From: Page MJ, McKenzie JE, Bossuyt PM, Boutron I, Hoffmann TC, Mulrow CD, et al. The PRISMA 2020 statement: an updated guideline for reporting systematic reviews. BMJ 2021;372:n71. doi: 10.1136/bmj.n71

For more information, visit: http://www.prisma-statement.org/

For peer review only - http://bmjopen.bmj.com/site/about/guidelines.xhtml

PRISMA Checklist

Section and Topic	Item #	Checklist item	Location where item is reported
**TITLE**			
Title	1	Identify the report as a systematic review.	Title page (p1)
**ABSTRACT**			
Abstract	2	See the PRISMA 2020 for Abstracts checklist.	Abstract (p2)
**INTRODUCTION**			
Rationale	3	Describe the rationale for the review in the context of existing knowledge.	Introduction (p4)
Objectives	4	Provide an explicit statement of the objective(s) or question(s) the review addresses.	Introduction (p4)
**METHODS**			
Eligibility criteria	5	Specify the inclusion and exclusion criteria for the review and how studies were grouped for the syntheses.	Methods: Inclusion and Exclusion criteria (p5)
Information sources	6	Specify all databases, registers, websites, organisations, reference lists and other sources searched or consulted to identify studies. Specify the date when each source was last searched or consulted.	Methods: Data sources and search strategy (p5)
Search strategy	7	Present the full search strategies for all databases, registers and websites, including any filters and limits used.	Methods: Data sources and search strategy (p5)
Selection process	8	Specify the methods used to decide whether a study met the inclusion criteria of the review, including how many reviewers screened each record and each report retrieved, whether they worked independently, and if applicable, details of automation tools used in the process.	Methods: Inclusion and Exclusion criteria (p5)
Data collection process	9	Specify the methods used to collect data from reports, including how many reviewers collected data from each report, whether they worked independently, any processes for obtaining or confirming data from study investigators, and if applicable, details of automation tools used in the process.	Methods: Data Extraction (p6)
Data items	10a	List and define all outcomes for which data were sought. Specify whether all results that were compatible with each outcome domain in each study were sought (e.g. for all measures, time points, analyses), and if not, the methods used to decide which results to collect.	Methods: Data Extraction (p6)
	10b	List and define all other variables for which data were sought (e.g. participant and intervention characteristics, funding sources). Describe any assumptions made about any missing or unclear information.	Methods: Data Extraction (p6)
Study risk of bias assessment	11	Specify the methods used to assess risk of bias in the included studies, including details of the tool(s) used, how many reviewers assessed each study and whether they worked independently, and if applicable, details of automation tools used in the process.	Methods: Assessment of bias (p6)
Effect measures	12	Specify for each outcome the effect measure(s) (e.g. risk ratio, mean difference) used in the synthesis or presentation of results.	Methods: Synthesis methods (p6)
Synthesis methods	13a	Describe the processes used to decide which studies were eligible for each synthesis (e.g. tabulating the study intervention characteristics and comparing against the planned groups for each synthesis (item #5)).	Methods: Synthesis methods (p6)
	13b	Describe any methods required to prepare the data for presentation or synthesis, such as handling of missing	Methods: Synthesis

Section and Topic	Item #	Checklist item	Location where item is reported
		summary statistics, or data conversions.	methods (p6)
	13c	Describe any methods used to tabulate or visually display results of individual studies and syntheses.	Methods: Synthesis methods (p6)
	13d	Describe any methods used to synthesize results and provide a rationale for the choice(s). If meta-analysis was performed, describe the model(s), method(s) to identify the presence and extent of statistical heterogeneity, and software package(s) used.	Methods: Synthesis methods (p6)
	13e	Describe any methods used to explore possible causes of heterogeneity among study results (e.g. subgroup analysis, meta-regression).	Not applicable
	13f	Describe any sensitivity analyses conducted to assess robustness of the synthesized results.	Not applicable
Reporting bias assessment	14	Describe any methods used to assess risk of bias due to missing results in a synthesis (arising from reporting biases).	Not applicable
Certainty assessment	15	Describe any methods used to assess certainty (or confidence) in the body of evidence for an outcome.	Not applicable
**RESULTS**			
Study selection	16a	Describe the results of the search and selection process, from the number of records identified in the search to the number of studies included in the review, ideally using a flow diagram.	Results: Study selection (p6-7); Figure 1. Flow diagram detailing results of searches and study selection process; Supplemental 2. Systematic review searches performed and results
	16b	Cite studies that might appear to meet the inclusion criteria, but which were excluded, and explain why they were excluded.	Results: Study selection (p6-7); Figure 1. Flow diagram detailing results of searches and study selection process; Supplemental 2. Systematic review searches performed and results
Study characteristics	17	Cite each included study and present its characteristics.	Results: Description of included studies (p7-8)
Risk of bias in studies	18	Present assessments of risk of bias for each included study.	Results: Quality assessment (p7); Supplemental 2 Assessment of bias for case-control studies

For peer review only

Section and Topic	Item #	Checklist item	Location where item is reported
			according to RoBANs; Supplemental 2 Assessment of bias of cohort studies using the Newcastle-Ottawa Quality Assessment form for cohort studies
Results of individual studies	19	For all outcomes, present, for each study: (a) summary statistics for each group (where appropriate) and (b) an effect estimate and its precision (e.g. confidence/credible interval), ideally using structured tables or plots.	Table 1. Characteristics of included studies
Results of syntheses	20a	For each synthesis, briefly summarise the characteristics and risk of bias among contributing studies.	Table 1. Characteristics of included studies<br>Figure 3. Relationship between serum 25-hydroxyvitamin D and Benign Paroxysmal Positional Vertigo recurrence.
	20b	Present results of all statistical syntheses conducted. If meta-analysis was done, present for each the summary estimate and its precision (e.g. confidence/credible interval) and measures of statistical heterogeneity. If comparing groups, describe the direction of the effect.	Results: Meta analysis (p8-9)<br>Figure 3. Relationship between serum 25-hydroxyvitamin D and Benign Paroxysmal Positional Vertigo recurrence.
	20c	Present results of all investigations of possible causes of heterogeneity among study results.	Not applicable
	20d	Present results of all sensitivity analyses conducted to assess the robustness of the synthesized results.	Not applicable
Reporting biases	21	Present assessments of risk of bias due to missing results (arising from reporting biases) for each synthesis assessed.	Not applicable
Certainty of evidence	22	Present assessments of certainty (or confidence) in the body of evidence for each outcome assessed.	Not applicable
**DISCUSSION**			
Discussion	23a	Provide a general interpretation of the results in the context of other evidence.	Discussion (p11-12)
	23b	Discuss any limitations of the evidence included in the review.	Strengths and limitations of the study (p3); Discussion (p11-12)

For peer review only

Section and Topic	Item #	Checklist item	Location where item is reported
	23c	Discuss any limitations of the review processes used.	Strengths and limitations (p3)
	23d	Discuss implications of the results for practice, policy, and future research.	Discussion (p11-12)
**OTHER INFORMATION**			Prospero registration number (p.2)
Registration and protocol	24a	Provide registration information for the review, including register name and registration number, or state that the review was not registered.	Abstract: Trial registration (p2)
	24b	Indicate where the review protocol can be accessed, or state that a protocol was not prepared.	Prospero registration number (p.2)
	24c	Describe and explain any amendments to information provided at registration or in the protocol.	Prospero registration number (p.2)
Support	25	Describe sources of financial or non-financial support for the review, and the role of the funders or sponsors in the review.	Funding sources (p14)
Competing interests	26	Declare any competing interests of review authors.	Competing interests statement (p14)
Availability of data, code and other materials	27	Report which of the following are publicly available and where they can be found: template data collection forms; data extracted from included studies; data used for all analyses; analytic code; any other materials used in the review.	Methods: Synthesis methods (p6) and Supplemental 2 tables 2 and 3

From: Page MJ, McKenzie JE, Bossuyt PM, Boutron I, Hoffmann TC, Mulrow CD, et al. The PRISMA 2020 statement: an updated guideline for reporting systematic reviews. BMJ 2021;372:n71. doi: 10.1136/bmj.n71

For more information, visit: http://www.prisma-statement.org/

For peer review only

PRISMA Abstract Checklist

Section and Topic	Item #	Checklist item	Reported (Yes/No)
**TITLE**			
Title	1	Identify the report as a systematic review.	YES
**BACKGROUND**			
Objectives	2	Provide an explicit statement of the main objective(s) or question(s) the review addresses.	YES
**METHODS**			
Eligibility criteria	3	Specify the inclusion and exclusion criteria for the review.	YES
Information sources	4	Specify the information sources (e.g. databases, registers) used to identify studies and the date when each was last searched.	YES
Risk of bias	5	Specify the methods used to assess risk of bias in the included studies.	YES
Synthesis of results	6	Specify the methods used to present and synthesise results.	YES
**RESULTS**			
Included studies	7	Give the total number of included studies and participants and summarise relevant characteristics of studies.	YES
Synthesis of results	8	Present results for main outcomes, preferably indicating the number of included studies and participants for each. If meta-analysis was done, report the summary estimate and confidence/credible interval. If comparing groups, indicate the direction of the effect (i.e. which group is favoured).	YES
**DISCUSSION**			
Limitations of evidence	9	Provide a brief summary of the limitations of the evidence included in the review (e.g. study risk of bias, inconsistency and imprecision).	YES
Interpretation	10	Provide a general interpretation of the results and important implications.	YES
**OTHER**			
Funding	11	Specify the primary source of funding for the review.	YES
Registration	12	Provide the register name and registration number.	YES

From: Page MJ, McKenzie JE, Bossuyt PM, Boutron I, Hoffmann TC, Mulrow CD, et al. The PRISMA 2020 statement: an updated guideline for reporting systematic reviews. BMJ 2021;372:n71. doi: 10.1136/bmj.n71

For more information, visit: http://www.prisma-statement.org/
